# Cryptochrome PtCPF1 regulates high temperature acclimation of marine diatoms through coordination of iron and phosphorus uptake

**DOI:** 10.1093/ismejo/wrad019

**Published:** 2024-01-10

**Authors:** Shan Gao, Wenting Yang, Xin Li, Lu Zhou, Xuehua Liu, Songcui Wu, Lijun Wang, Guangce Wang

**Affiliations:** CAS and Shandong Province Key Laboratory of Experimental Marine Biology, Center for Ocean Mega-Science, Institute of Oceanology, Chinese Academy of Sciences, Qingdao 266071, China; Laboratory for Marine Biology and Biotechnology, Qingdao National Laboratory for Marine Science and Technology, Qingdao 266237, China; Key Laboratory of Breeding Biotechnology and Sustainable Aquaculture, Chinese Academy of Sciences, Qingdao 266071, China; CAS and Shandong Province Key Laboratory of Experimental Marine Biology, Center for Ocean Mega-Science, Institute of Oceanology, Chinese Academy of Sciences, Qingdao 266071, China; Laboratory for Marine Biology and Biotechnology, Qingdao National Laboratory for Marine Science and Technology, Qingdao 266237, China; Key Laboratory of Breeding Biotechnology and Sustainable Aquaculture, Chinese Academy of Sciences, Qingdao 266071, China; CAS and Shandong Province Key Laboratory of Experimental Marine Biology, Center for Ocean Mega-Science, Institute of Oceanology, Chinese Academy of Sciences, Qingdao 266071, China; Laboratory for Marine Biology and Biotechnology, Qingdao National Laboratory for Marine Science and Technology, Qingdao 266237, China; Key Laboratory of Breeding Biotechnology and Sustainable Aquaculture, Chinese Academy of Sciences, Qingdao 266071, China; College of Earth Sciences, University of Chinese Academy of Sciences, Beijing 100049, China; CAS and Shandong Province Key Laboratory of Experimental Marine Biology, Center for Ocean Mega-Science, Institute of Oceanology, Chinese Academy of Sciences, Qingdao 266071, China; Laboratory for Marine Biology and Biotechnology, Qingdao National Laboratory for Marine Science and Technology, Qingdao 266237, China; Key Laboratory of Breeding Biotechnology and Sustainable Aquaculture, Chinese Academy of Sciences, Qingdao 266071, China; CAS and Shandong Province Key Laboratory of Experimental Marine Biology, Center for Ocean Mega-Science, Institute of Oceanology, Chinese Academy of Sciences, Qingdao 266071, China; Laboratory for Marine Biology and Biotechnology, Qingdao National Laboratory for Marine Science and Technology, Qingdao 266237, China; Key Laboratory of Breeding Biotechnology and Sustainable Aquaculture, Chinese Academy of Sciences, Qingdao 266071, China; CAS and Shandong Province Key Laboratory of Experimental Marine Biology, Center for Ocean Mega-Science, Institute of Oceanology, Chinese Academy of Sciences, Qingdao 266071, China; Laboratory for Marine Biology and Biotechnology, Qingdao National Laboratory for Marine Science and Technology, Qingdao 266237, China; Key Laboratory of Breeding Biotechnology and Sustainable Aquaculture, Chinese Academy of Sciences, Qingdao 266071, China; CAS and Shandong Province Key Laboratory of Experimental Marine Biology, Center for Ocean Mega-Science, Institute of Oceanology, Chinese Academy of Sciences, Qingdao 266071, China; Laboratory for Marine Biology and Biotechnology, Qingdao National Laboratory for Marine Science and Technology, Qingdao 266237, China; Key Laboratory of Breeding Biotechnology and Sustainable Aquaculture, Chinese Academy of Sciences, Qingdao 266071, China; CAS and Shandong Province Key Laboratory of Experimental Marine Biology, Center for Ocean Mega-Science, Institute of Oceanology, Chinese Academy of Sciences, Qingdao 266071, China; Laboratory for Marine Biology and Biotechnology, Qingdao National Laboratory for Marine Science and Technology, Qingdao 266237, China; Key Laboratory of Breeding Biotechnology and Sustainable Aquaculture, Chinese Academy of Sciences, Qingdao 266071, China

**Keywords:** cryptochrome, diatoms, high temperature, iron and phosphorus uptake

## Abstract

Increasing ocean temperatures threaten the productivity and species composition of marine diatoms. High temperature response and regulation are important for the acclimation of marine diatoms to such environments. However, the molecular mechanisms behind their acclimation to high temperature are still largely unknown. In this study, the abundance of *PtCPF1* homologs (a member of the cryptochrome-photolyase family in the model diatom *Phaeodactylum tricornutum*) transcripts in marine phytoplankton is shown to increase with rising temperature based on *Tara* Oceans datasets. Moreover, the expression of *PtCPF1* in *P. tricornutum* at high temperature (26 °C) was much higher than that at optimum temperature (20 °C). Deletion of *PtCPF1* in *P. tricornutum* disrupted the expression of genes encoding two phytotransferrins (ISIP2A and ISIP1) and two Na^+^/P co-transporters (PHATRDRAFT_47667 and PHATRDRAFT_40433) at 26 °C. This further impacted the uptake of Fe and P, and eventually caused the arrest of cell division. Gene expression, Fe and P uptake, and cell division were restored by rescue with the native *PtCPF1* gene. Furthermore, PtCPF1 interacts with two putative transcription factors (BolA and TF IIA) that potentially regulate the expression of genes encoding phytotransferrins and Na^+^/P co-transporters. To the best of our knowledge, this is the first study to reveal PtCPF1 as an essential regulator in the acclimation of marine diatoms to high temperature through the coordination of Fe and P uptake. Therefore, these findings help elucidate how marine diatoms acclimate to high temperature.

## Introduction

Ocean warming including marine heatwaves is projected to continue over the coming century, with serious implications for marine ecosystems [1, 2]. Increasing ocean temperatures threaten the productivity and species composition of marine diatoms. Diatoms are ubiquitous phytoplankton in the ocean responsible for ~20% of global primary productivity, playing essential roles in food webs and biogeochemical cycling [3-6]. Evidence suggests that they show a high plasticity in adapting to dynamic environmental conditions [7-9]; however, they are sensitive to temperature changes, particularly high temperature [10, 11]. For example, *Phaeodactylum tricornutum*, a model marine diatom, is sensitive to high temperature. Although it can tolerate low temperature (10 °C), it has a slower growth rate and with potential mortality at high temperature conditions (>26 °C) [12]. Although the effects of temperatures on the growth rate and physiological characteristics of marine diatoms are well understood [4, 12, 13], studies on the molecular mechanisms of their acclimation to high temperature are scarce.

Recently, the blue light photoreceptor cryptochromes in *Arabidopsis* were reported to play important regulatory roles in response to high temperature [14-16]. These conserved photolyase-like photoreceptors are found in almost all species including marine diatoms [17]. *Arabidopsis* cryptochrome (Cry) 1 represses high temperature-induced hypocotyl elongation [16], and Cry2 regulates thermosensory flowering in a blue light-dependent manner [15]. In diatoms, all types of cryptochromes and photolyases have been predicted from genome sequences, and seven genes for proteins of the cryptochrome-photolyase family (CPF) have been identified in *P. tricornutum* [7, 18]. To date, only two cryptochromes (PtCPF1 and CryP) in *P. tricornutum* have been analyzed for their biochemical functions [19-22]; however, their regulatory roles, especially the downstream signal transduction from these two photoreceptors under different environments, remain largely unknown. Although Coesel *et al*. found that PtCPF1 is a new CPF family member with transcription regulation and DNA repair activity, they suggested that generation of *PtCPF1* knockdown or knockout (KO) mutants was required to investigate its novel biological functions in marine diatoms. Considering the specific functions of cryptochromes in *Arabidopsis*, this study addressed the question of whether PtCPF1 functioned in the high temperature acclimation of marine diatoms.

By analyzing existing transcriptomic data from *Tara* Oceans datasets, this study reports a wide occurrence and expression of homologous *PtCPF1* (PHATRDRAFT_27429, NCBI Gene ID: 7201137) genes in phytoplankton in the global ocean, and strong responses of diatom *PtCPF1* to high temperature stimuli. By generating *PtCPF1* KO mutants in *P. tricornutum* through CRISPR/Cas9 and rescuing the KO mutants with the native *PtCPF1* gene, along with the interaction with putative transcription factors (TFs; BolA and TF IIA), PtCPF1 is shown to regulate the expression of two phytotransferrins (ISIP2A and ISIP1) and two Na^+^/P co-transporters (PHATRDRAFT_47667 and PHATRDRAFT_40433), especially at high temperature. This further impacts the uptake of Fe and P and eventually affected cell division. These results reveal a new mechanism in which PtCPF1 in marine diatoms regulated Fe and P uptake to acclimate to high temperature.

## Results

### Widespread occurrence and responses of *PtCPF1* to high temperature

**Figure 1:**
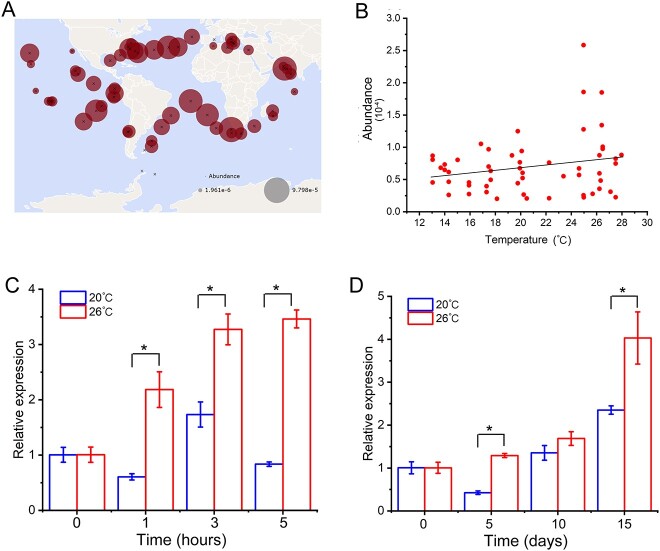
Widespread occurrence and temperature responses of *PtCPF1* in marine phytoplankton; (A) wide geographic distribution of *PtCPF1* in marine phytoplankton found in the *Tara* oceans dataset; the circle size represents the *PtCPF1* mRNA abundance based on the 20–180 μm size fraction from the surface layer and a temperature range of 10–30 °C from *Tara* Ocean datasets; the circle sizes indicate the *PtCPF1* homolog abundance at the sampling stations; the unit for abundance is percent of total reads; (B) analysis of the relationship between the abundance of *PtCPF1* mRNA and temperature; the mRNA abundance of *PtCPF1* homologs in phytoplankton that meet the set parameters (0.8–2000 μm, 10–30 °C) and their sampling stations from *Tara* Ocean datasets; the unit for abundance is percent of total reads; the linear relationship between temperature and transcript abundance of PtCPF1 homologs were evaluated by regression analysis and Durbin–Watson test (*P* < .05); relative expression of *PtPCF1* in *P. tricornutum* under short-term treatment (0, 1, 3, and 5 h) of 20 and 26 °C (C) and long-term treatment (15 days) of 20 and 26 °C (D) determined by quantitative RT-PCR; data are presented as the mean ± SD (*n* = 3 biologically independent experiments); asterisks indicate a significant difference between two groups; independent samples *t*-tests were used to compare the two groups (*P* < .05).

To assess whether *PtCPF1* broadly exists in marine phytoplankton, the occurrence of *PtCPF1* and temperature stimuli regulating its expression was investigated based on *Tara* Oceans unigene and metatranscriptome datasets. Analyses were restricted to the 5–20 and 20–180 μm size fractions from the surface layer and a temperature range of 10–30 °C. From *Tara* Oceans and metatranscriptomes datasets, *PtCPF1* homologs were found at major sampling stations worldwide (Fig. 1A and Supplementary Fig. S1). It revealed that *PtCPF1* was geographically distributed in marine phytoplankton across different temperature conditions. Moreover, higher temperature could cause the expression of *PtCPF1* transcripts in marine phytoplankton (Fig. 1B and Supplementary Fig. S2). To make a comparison, the effects of other environmental variables, including photosynthetic active radiation, salinity, and NO_3_^−^, on the abundance of *PtCPF1* homologs transcripts were also analyzed, which were evaluated by Pearson and Spearman coefficients (Supplementary Tables S1 and S2), suggesting that only temperature has a stable positive correlation with *PtCPF1* homologs transcripts abundance. This demonstrated that PtCPF1 may be important in the adaptation of marine phytoplankton to high temperature conditions (20–30 °C).

To further explore the expression of *PtCPF1* at different temperatures, experiments were conducted on the model diatom *P. tricornutum*. Based on quantitative reverse transcription polymerase chain reaction (qRT-PCR), under light conditions, *PtCPF1* expression increased over the short- (from 1 to 5 h) and long-term (from 5 to 15 days) high temperature treatments (26 °C) compared with the optimal temperature treatment (20 °C) (Fig. 1C and D). Moreover, even under the dark condition, *PtCPF1* expression also showed an increasing trend after 3 and 5 h of high temperature (Supplementary Fig. S3A). These results suggested that there was a significant difference between the samples for most time points, independent of light conditions. Hence, it is postulated that PtCPF1 had an important function in the response of phytoplankton to high temperature.

### Involvement of PtCPF1 in the high temperature response

**Figure 2:**
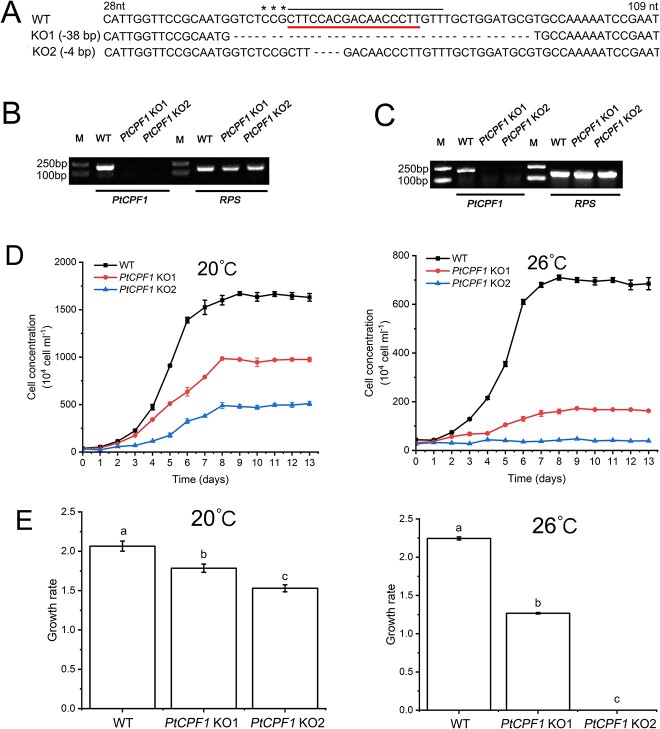
Generations and characteristics of *PtCPF1* mutants in *P. Tricornutum*; (A) Alignment of partial *PtCPF1* sequences of the CRISPR/Cas9-generated mutants showing frameshift indels compared with the WT; the reverse complement counterparts of gRNA and PAM are marked by topline and asterisks, respectively; the deleted bases are marked by a dashed line; a pair of primers with one located in the region of deletion indicated by underline and the other outside it corresponding to the nonediting region (between 253 and 269 nt); primers shown by red underline in (A) were used to detect the deletions in the two *PtCPF1*-KO mutants with PCR using genomic DNA (B) and cDNA (C) as templates; DNA controls amplified a fragment of the gene encoded 30S ribosomal protein subunit; (D) growth curves of the two *PtCPF1* KO mutants and WT at 20 and 26 °C; samples were taken daily for cell counting; data are presented as the mean ± SD (*n* = 3 biological independent experiments); starting cell density was 4 × 10^5^ cells ml^−1^; (E) growth rates of the two *PtCPF1* KO mutants and WT at 20 and 26 °C; the growth rate was estimated from cell concentration using the equation indicated in the Materials and Methods section; different lowercase letters indicate statistically significant differences, as determined by one-way ANOVA with Tukey’s multiple comparisons test (*P* < .05).

To investigate the function of PtCPF1 in high temperature responses, using an optimized efficient CRISPR/Cas9 gene editing system (Supplementary Table S3), CRISPR/Cas9 KO mutants of *PtCPF1* in *P. tricornutum* were generated. Three heterozygous *PtCPF1* KO mutants with different mutant characteristics (Supplementary Fig. S4) were initially obtained, all of which were sensitive to high temperature (Supplementary Fig. S5). After several rounds of screening by plating heterozygous clones onto plates, two homozygous *PtCPF1* KO mutants with different mutant characteristics (named *PtCPF1* KO1 and *PtCPF1* KO2) were obtained. Thirty-eight and four bases were deleted in *PtCPF1* KO1 and *PtCPF1* KO2, respectively (Fig. 2A and Supplementary Fig. S6). To confirm the absence of the wild-type (WT) gene (*PtCPF1*) using PCR with genomic DNA prepared from the two mutants, a pair of primers was designed with one located in the region of deletion and the other outside it corresponding to the nonediting region indicated by red underline (Fig. 2A). The expected DNA band was observed in the WT, whereas there was no band in *PtCPF1* KO1 and *PtCPF1* KO2 mutants (Fig. 2B).

The above-mentioned primers were further used to detect transcripts of *PtCPF1* using PCR with cDNA prepared from the two *PtCPF1* KO mutants; no expected band was observed in the two *PtCPF1* KO mutants (Fig. 2C). The physiology of the two *PtCPF1* KO mutants was further analyzed at different temperature conditions. At 20 °C, the growth rates of *PtCPF1* KO1 and *PtCPF1* KO2 greatly decreased in comparison with that of the WT, which decreased more obviously at 26 °C (Fig. 2D and E). The growth of *PtCPF1* KO2 stagnated at 26 °C (Fig. 2E). Moreover, the photosynthetic activity of the two *PtCPF1* KO mutants at 20 °C and 26 °C changed in comparison with that of the WT (Supplementary Fig. S7). Particularly, the Y(II) [the effective photochemical quantum yield of Photosystem II (PSII)] of the two *PtCPF1* KO mutants at 20 °C and 26 °C decreased significantly in comparison with that of the WT (Supplementary Fig. S7A and B), but there was no significant changes in Y(I) [the effective photochemical quantum yield of Photosystem I (PSI)] between the WT and the two *PtCPF1* KO mutants at 20 and 26 °C (Supplementary Fig. S7C and D).

**Figure 3:**
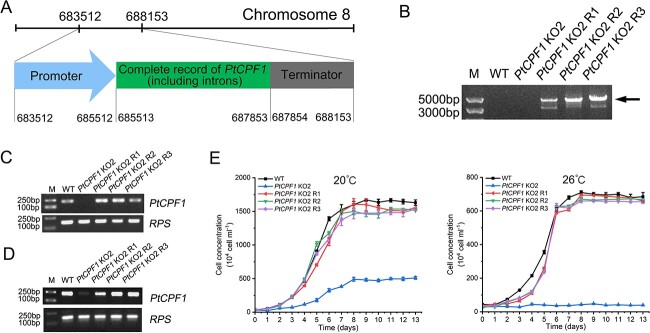
Rescue of *PtCPF1* KO2 mutant with native *PtCPF1* and characteristics of the rescued lines; (A) scheme showing the native *PtCPF1* including the promoter, complete record (including introns), and terminator in *P. tricornutum*; the length of the promoter is 2000 bp upstream the ATG codon; the length of the terminator is 300 bp downstream the stop codon; (B) agarose gel of PCR products from genomic DNA in the WT, *PtCPF1* KO2, and the rescued lines (*PtCPF1* KO2 R1, R2, and R3) using the primers located in the vectors; agarose gel of PCR products from genomic DNA (C) and cDNA (D) in the WT, *PtCPF1* KO2, and the rescued lines (*PtCPF1* KO2 R1, R2, and R3) using the primers shown by underline in Fig. 2A; DNA controls amplified a fragment of the gene encoded 30S ribosomal protein subunit; (E) growth curves of the WT, *PtCPF1* KO2, and the rescued lines (*PtCPF1* KO2 R1, R2, and R3) at 20 and 26 °C; samples were taken daily for cell counting; data are presented as the mean ± SD (*n* = 3 biological independent experiments); starting cell density was 4 × 10^5^ cells ml^−1^.

To further confirm the function of PtCPF1 at different temperature conditions, *PtCPF1* KO2 was rescued with the native *PtCPF1*; the genomic sequence, including the promoter and terminator, was PCR amplified from *P. tricornutum* genomic DNA (Fig. 3A). Three rescued lines of *PtCPF1* KO2 (named *PtCPF1* KO2 R1, R2, and R3) were obtained. To confirm the presence of the complemented *PtCPF1* in the three *PtCPF1* KO2 rescued lines, PCR was conducted with genomic DNA from the WT, *PtCPF1* KO2, and rescued lines (*PtCPF1* KO2 R1, R2, and R3) to amplify the complemented *PtCPF1* using the primers located in the vectors (Supplementary Table S4). There were expected bands, indicated by the arrow in the three rescued lines, whereas there was no band observed in the WT and *PtCPF1* KO2 (Fig. 3B). The above-mentioned primers shown in Fig. 2A were used to detect the presence of the complemented *PtCPF1* using PCR with genomic DNA and cDNA prepared from the WT, *PtCPF1* KO2, and the three rescued lines, and the expected bands were observed in the three rescued lines (Fig. 3C and D). Furthermore, the three rescued lines demonstrated similar growth curves to that of the wild type at 20 and 26 °C (Fig. 3E). Based on the above results, it was concluded that PtCPF1 participated in the acclimation of *P. tricornutum* to high temperature.

### PtCPF1 positively regulates Fe and P uptake, and N metabolism especially at high temperature

**Figure 4:**
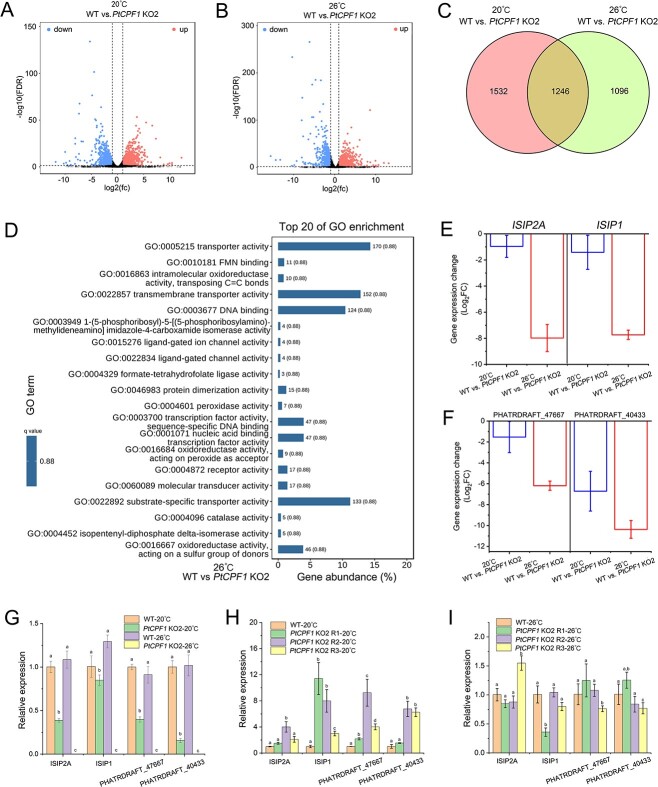
PtCPF1 is required for the expression of genes involved in the uptake of Fe and P at high temperature; volcano plot of DEGs in *PtCPF1* KO2 compared with the WT at 20 °C (control) (A) or 26 °C (B) determined by mRNA sequencing; WT and *PtCPF1* KO2 were grown for 7 days at 20 and 26 °C and were harvested for mRNA-sequencing; (C) Venn diagram showing the overlap of DEGs shown in (A) and (B); (D) GO enrichment analysis (molecular functions) of the wild type versus *PtCPF1* KO2 at 26 °C; the numbers behind the bars mean the gene counts in the specific GO term; the q-value is identical for each GO term; (E) expression patterns of genes encoding two phytotransferrins (ISIP2A, PHATRDRAFT_54465, and ISIP1, PHATRDRAFT_55031) of *PtCPF1* KO2 mutant at 20 and 26 °C. FC, fold-change; data are presented as the mean ± SD (*n* = 3 biological independent experiments); (F) expression patterns of genes encoding two Na^+^/P co-transporters (PHATRDRAFT_47667 and PHATRDRAFT_40433) of *PtCPF1* KO2 mutant at 20 and 26 °C; data are presented as the mean ± SD (*n* = 3 biological independent experiments); relative expression of *ISIP2A* and *ISIP1*, and PHATRDRAFT_47667 and PHATRDRAFT_40433 in the WT and *PtCPF1* KO2 at 20 (G) and 26 °C (H), as determined by qRT-PCR; asterisks indicate a significant difference between two groups; independent sample *t*-tests were used to compare two groups (*P* < .05); (I) relative expression of *ISIP2A* and *ISIP1*, and PHATRDRAFT_47667 and PHATRDRAFT_40433 in the WT and rescued lines (*PtCPF1* KO2 R1, R2, and R3) at 26 °C as determined by qRT-PCR; error bars represent the mean ± SD (*n* = 3 biologically independent experiments).

To dissect the role of PtCPF1 at high temperature, transcriptomic profiling of the WT and *PtCPF1* KO2 cells was performed at 20 and 26 °C (Fig. 4, Supplementary Figs S8 and S9A). Differentially expressed genes (DEGs) were identified in *PtCPF1* KO2 compared with the WT at 20 and 26 °C, which were indicated by volcano plot and Venn diagram (Fig. 4A–C). The results of the Gene Ontology (GO) enrichment analysis suggested that many transporters were significantly decreased in *PtCPF1* KO2 compared with the WT at 26 °C (Fig. 4D). According to the transcriptomic data, we sorted the DEGs between WT and *PtCPF1* KO2 at 20 and 26 °C. We found that the expression of two phytotransferrins (ISIP2A and ISIP1, iron starvation induced protein) and two Na^+^/P co-transporters (PHATRDRAFT_47667 and PHATRDRAFT_40433) were significant in in *PtCPF1* KO2 compared with the WT, especially at 26 °C (Fig. 4E and F). Moreover, at 26 °C, the transcripts of the two phytotransferrins and two Na^+^/P co-transporters were not detected (Supplementary Fig. S9B), suggesting that the expression of these genes was disrupted at this temperature. These results were further confirmed by qRT-PCR (Fig. 4G and Supplementary Table S5). In addition, the expressions of these genes in the rescued lines (*PtCPF1* KO2 R1, R2, and R3) were restored at 20 and 26 °C (Fig. 4H and I). Therefore, it was concluded that PtCPF1 positively regulated the expression of genes encoding the two phytotransferrins (ISIP2A and ISIP1) and Na^+^/P co-transporters, especially at high temperature.

**Figure 5:**
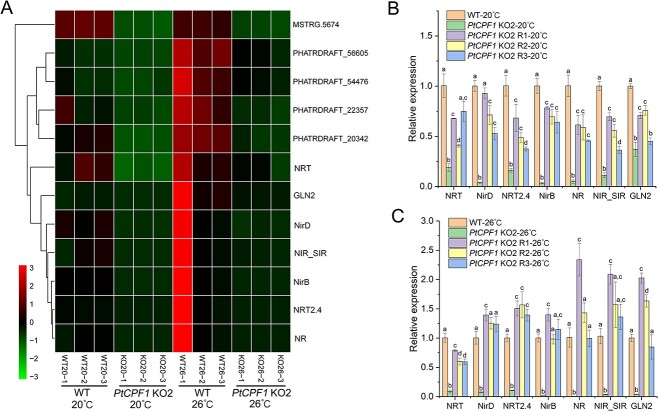
N assimilation is also affected by PtCPF1; (A) heat maps showing the relative expression changes of N assimilation genes under indicated conditions; (B) relative expression of major NO_3−_ -uptake and N assimilation genes in the WT, *PtCPF1* KO2, rescued lines (*PtCPF1* KO2 R1, R2, and R3) under indicated conditions as determined by qRT-PCR; (C) relative expression of N assimilation genes in the WT and rescued lines (*PtCPF1* KO2 R1, R2, and R3) under indicated conditions as determined by qRT-PCR; data are presented as mean ± SD (*n* = 3 biologically independent experiments); NRT, nitrate transporter (PHATRDRAFT_54101); NirD, nitrite reductase D (PHATRDRAFT_8155); NRT2.4, nitrate transporter 2.4 (PHATRDRAFT_26029); NirB, nitrite reductase B (PHATRDRAFT_13154); NR, nitrate reductase (PHATRDRAFT_54983); NIR_SIR, nitrite and sulfite reductase (PHATRDRAFT_27757); and GLN2, glutamine synthetase 2 (PHATRDRAFT_51092).

In addition, the Kyoto Encyclopedia of Genes and Genomes enrichment analysis results suggested that N metabolism was significantly altered in *PtCPF1* KO2 compared with the WT at 20 and 26 °C (Fig. 5 and Supplementary Fig. S8C and D). Furthermore, among the DEGs, most of the N metabolism genes [mainly encoding nitrate transporters (NRT), nitrate reductase (NR), nitrite reductases (NiR), and glutamine synthetase (GLN), among others] especially at 26 °C were significantly downregulated in *PtCPF1* KO2 compared with the WT (Fig. 5A). The expression of these genes in *PtCPF1* KO2 at 20 and 26 °C was further confirmed based on qRT-PCR (Fig. 5B and C, and Supplementary Table S5), which was consistent with the transcriptomic data. The expression of the genes involving N metabolism in the *PtCPF1* KO2 rescued lines (*PtCPF1* KO2 R1, R2, and R3) was restored at 20 and 26 °C (Fig. 5B and C), further indicating that PtCPF1 positively regulated N metabolism, especially at 26 °C. Additionally, based on the transcriptomic data, the expression of ascorbate peroxidases (APX) (PHATRDRAFT_13174 and PHATRDRAFT_46616), peroxidase (POD) domain-containing protein (PHATRDRAFT_bd1645), and catalase (CAT) (PHATR_13244), especially at 26 °C was significantly downregulated in *PtCPF1* KO2 compared with the WT (Supplementary Fig. S10), while the expression of the genes encoded heat shock protein (HSP) and heat shock factor (HSF) at 26 °C was significantly upregulated in *PtCPF1* KO2 compared with the WT (Supplementary Fig. S11).

**Figure 6:**
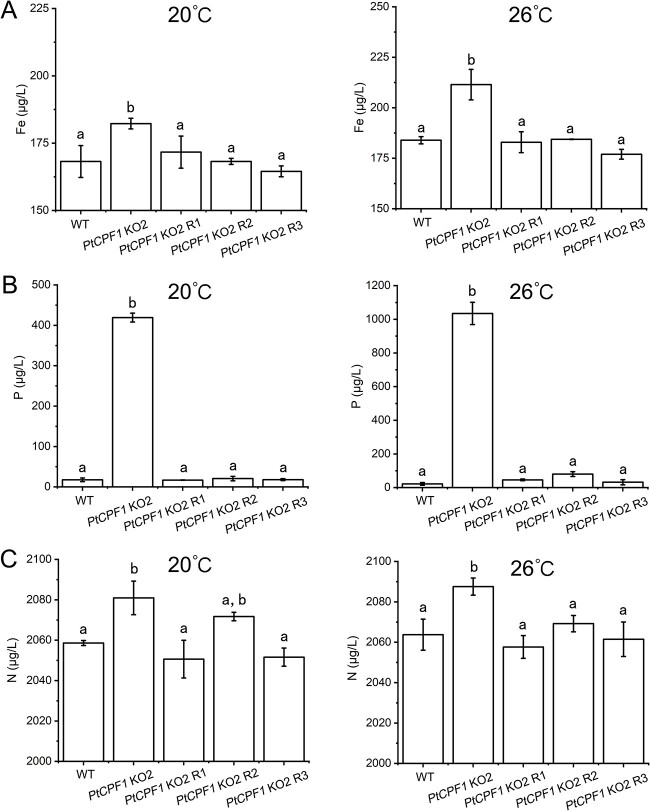
Contents of Fe, P, and N in the cultures of the WT, *PtCPF1* KO2, and the rescued lines (*PtCPF1* KO2 R1, R2, and R3), in which cells were removed; (A) Fe content in the cultures of *PtCPF1* KO2 especially at 26 °C was higher than that of the WT and rescued lines; (B) P content in the cultures of *PtCPF1* KO2 at 20 and 26 °C was much higher than that that of the WT and the rescued lines, indicating that the activity of P uptake was lost once PtCPF1 was knocked-out, especially at 26 °C; (C) N contents in the cultures of *PtCPF1* KO2 were also higher than that those of the WT and rescued lines; data are presented as the mean ± SD (*n* = 3 biological independent experiments); different lowercase letters indicate statistically significant differences, as determined by one-way ANOVA with Tukey’s multiple comparisons test (*P* < .05).

The contents of Fe, P, and N were determined in the cultures in which cells had been removed through centrifugation. The Fe content in the cultures of *PtCPF1* KO2 especially at 26 °C was higher than that of the WT and the rescued lines, indicating that the activity of Fe uptake was affected by PtCPF1, especially at high temperature (Fig. 6A). Of note, the P contents in the cultures of *PtCPF1* KO2 at 20 and 26 °C were > 400 and >1000 μg/l, respectively, which were significantly higher than those of the WT and the rescued lines (<100 μg/l) (Fig. 6B and Supplementary Fig. S12). These results suggested that P uptake activity was lost once PtCPF1 was knocked-out, especially at 26 °C, consistent with the transcriptomic data and qRT-PCR results. In addition, the N contents in the culture medium of *PtCPF1* KO2 were also higher than those of the WT and the rescued lines (Fig. 6C). These results suggested that the effect of PtCPF1 on P uptake was much greater than that on Fe and N uptake. Moreover, the content of P, Fe, and N significantly correlated with the growth rate of WT and *PtCPF1* KO2 at 26 °C (Supplementary Fig. S13). It was therefore proposed that PtCPF1 regulated the uptake of Fe and P, and N metabolism, especially at high temperature.

### Identification of PtCPF1 interaction partners

**Figure 7:**
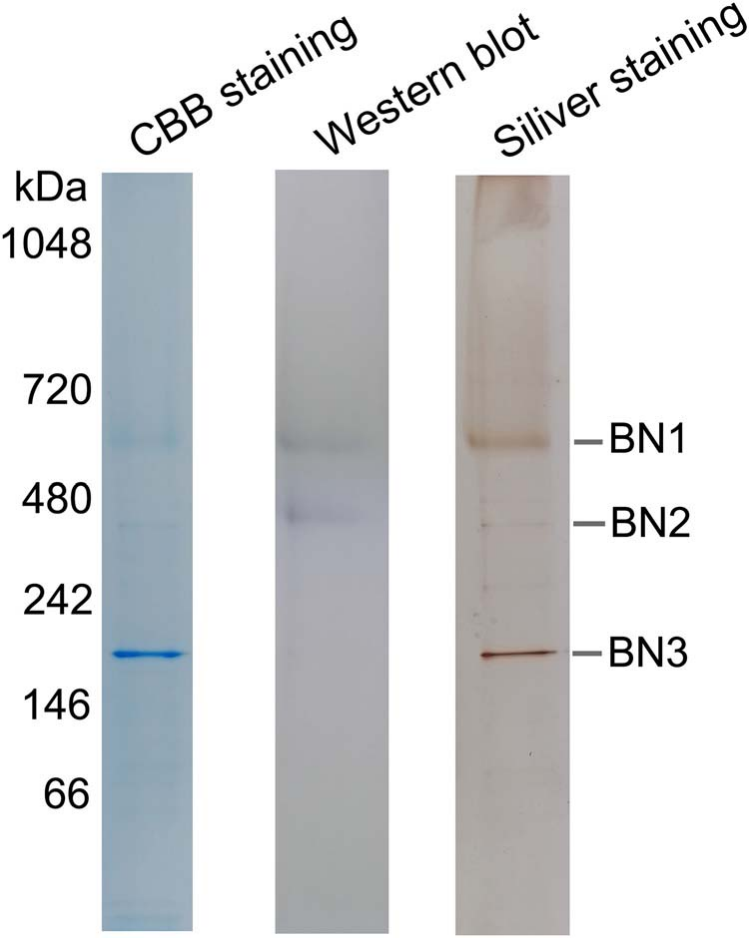
Identification of PtCPF1 interaction partners; *PtCPF1*-His_6_ lines were adapted in the dark for 60 h and then exposed to the light (450 nm, 20 μmol photons m^−2^ s^−1^) for 1 h; subsequently, the cultures were collected; the whole-cell extracts of *PtCPF1*-His_6_ lines were added to a nickel column; elution fractions were run on blue native-PAGE; the BN gel was followed by CBB staining, silver staining, and immunoblotting analysis; three bands (BN1, BN2, and BN3) are indicated.

As mentioned above, PtCPF1 regulates transcription, either via direct interaction with DNA or via protein interaction partners. Although PtCPF1 can bind DNA and show photolyase activity, its transcription regulation activity is still unclear. To identify the interaction partners of PtCPF1, pull-down experiments were carried out. *PtCPF1*-His_6_ lines were generated, which were further treated. Protein cell extract from *PtCPF1*-His_6_ lines was then added to a nickel-column. The elution proteins were separated by Blue Native Polyacrylamide Gel Electrophoresis (BN-PAGE), followed by Coomassie Brilliant Blue (CBB) staining, silver staining, and immunoblotting analysis (Fig. 7). Three Blue Native bands (indicated as BN1, BN2, and BN3) each with a different kDa, were observed; they were excised from the BN gel with CBB staining and assessed by liquid chromatography electrospray ionization tandem mass spectrometric (LC-ESI-MS/MS) analysis. Only proteins that were mainly implicated in signal transduction were listed, and unique peptides and total peptides were shown (for more details, see Supplementary Table S6) including ubiquitin extension protein 1/2, Eps15 (epidermal growth factor receptor pathway substrate 15) homology domain, the nucleotide-binding domain of the sugar kinase/HSP70/actin superfamily, two HSP70 (HSP70_2 and HSP70A), and two TFs (BolA type and TF IIA type) (Supplementary Table S7). Since PtCPF1 possesses potentially transcription regulation activity (Figs 4 and 5), we subsequently focused on the analysis of the two TF (BolA and TF IIA). Bioinformatic analysis of BolA (PHATRDRAFT_14849) suggested that it was similar to the two TF families of Trihelix and GRAS (named after the characteristic letters of the first discovered members GAI, RGA, and SCR) (Supplementary Fig. S14A). Moreover, TF IIA (PHATRDRAFT_42776) was similar to the two TF families of G2-like and bHLH (basic Helix–Loop–Helix) (Supplementary Fig. S14B). Bioinformatic predication analysis of the target genes of BolA and TF IIA suggested that BolA could not bind any regions in the promoters of two Na^+^/P co-transporters and phytotransferrins, whereas predication analysis suggested that TF IIA could bind the two conserved regions in the promoters of the two Na^+^/P co-transporters and ISIP2A (Supplementary Table S8).

## Discussion

Increasing ocean temperature negatively impacts the productivity and species composition of marine diatoms [3]. High temperature responses and regulation are important in the adaptation of marine diatoms to these environments. In this study, PtCPF1 was found to regulate high temperature acclimation in marine diatoms through the coordination of Fe and P uptake. PtCPF1 is a member of CPF family in diatoms, demonstrating DNA repair and transcription regulation activity [19]. This is different from the cryptochromes (Cry1 and 2) in higher plants, which only possess transcription regulation activity without the function of DNA repair [17]. Although the expression of PtCPF1 is regulated by blue light [19], based on the *Tara* Oceans and metatranscriptomes dataset, we found that the expression of homologous *PtCPF1* gene in marine phytoplankton was also shown to be modulated by different environmental variables (Fig. 1A and B, and Supplementary Tables S1 and S2). Based on the Pearson’s *r* value and the corresponding *P* value (*P* < .01), temperature and NO_3_^−^ with *PtCPF1* homologs transcripts abundance were positive correlation, while salinity negatively correlated with the abundance of *PtCPF1* homologs transcripts (Supplementary Tables S1 and S9). In comparison, based on the Spearman’s *r* value and the corresponding *P* value (*P* < .01), only temperature and salinity were correlated with *PtCPF1* homolog transcripts abundance, nevertheless they were positive and negative correlation, respectively. However, there was no correlation of NO_3_^−^ with *PtCPF1* homologs transcripts abundance (Supplementary Tables S2 and S9). Therefore, the correlation analysis based on Pearson and Spearman coefficients indicated that only temperature has a stable positive correlation with the abundance of *PtCPF1* homologs transcripts. More importantly, in the laboratory, the expression of *PtCPF1* in *P. tricornutum* demonstrated significant difference between the samples for most time points at high temperature condition (Fig. 1C and D, and Supplementary Fig. S3A), although its expression fluctuate at 20 °C which might cause by circadian clock regulation (Supplementary Fig. S3B) [23].

Once *PtCPF1* was knocked-out, the growth of *PtCPF1* KO mutants was much slower than that of the WT, and even stagnated at 26 °C (Fig. 2D and E), but it was restored in the rescued lines (Fig. 3E). These results demonstrated that PtCPF1 played a critical role in the acclimation of *P. tricornutum* to high temperature. Furthermore, *PtCPF1* KO2 demonstrated a significantly diminished expression of ISIP2A and ISIP1 (Fig. 4E), both of which were not expressed at 26 °C (Fig. 4G and Supplementary Fig. S9B). ISIP2A and ISIP1 are two phytotransferrins that play important roles in the uptake of Fe in marine diatoms [24-26]. In fact, the acquisition and homeostasis of Fe are critical for photosynthetic organisms to resist abiotic stresses including high temperature, and Fe is a key component of enzymatic antioxidants such as CAT, POD, and APX [27]. According to the transcriptomic analysis, the expression of the genes encoding these antioxidant enzymes in *PtCPF1* KO2 decreased significantly at 20 and 26 °C (Supplementary Fig. S10). In addition, at 26 °C, two Na^+^/P co-transporters (PHATRDRAFT_47667 and PHATRDRAFT_40433) in *PtCPF1* KO2 were not expressed (Fig. 4F and G and Supplementary Fig. S9C). Under P deficiency, the transcripts encoding the two Na^+^/P co-transporters in marine diatoms were upregulated significantly, suggesting that they played important roles in P uptake [28, 29]. P is an essential macronutrient, and a key component of nucleic acids and phospholipids; it plays a critical role in many biological processes [30]. In nutrient-starved *P. tricornutum* (including P, Fe, and N deficiency), the rate of cell division decreases significantly and the cell cycle is mainly arrested in the late G1 phase [31]. Therefore, it was concluded that at 26 °C, PtCPF1 was required for the expression of ISIP2A and ISIP1, and two Na^+^/P co-transporters in *P. tricornutum*.

Under Fe limitation, marine diatom genes for N assimilation, such as NR, NiR, and NRT, were downregulated, suggesting a reduced capacity for NO_3_^−^ assimilation [26, 32]. The expression of genes involving N assimilation (NRT, NR, NiR, and GLN) was downregulated in *PtCPF1* KO2 at 20 and 26 °C (Fig. 5), suggesting that N assimilation was positively affected by PtCPF1. Furthermore, N assimilation is also significantly correlated with the P level in diatoms [33]. Of note, the expressions of genes encoding HSP and HSFs in *PtCPF1* KO2 at 20 and 26 °C increased significantly in comparison with the WT (Supplementary Fig. S11A and B) and were restored in the rescued lines (Supplementary Fig. S11C). Actually, N deficiency results in the upregulation of HSF and HSP in marine diatoms, which might play a role in sensing and/or responding to N stress [34, 35]. Furthermore, Fe and P contents in the cultures further suggested that PtCPF1 regulated their uptake (Fig. 6). Therefore, it is proposed that at high temperature, the KO of *PtCPF1* in *P. tricornutum* caused the expression failure of the genes encoding Fe and P transporters. Therefore, we speculated that Fe and P deficiency in the *PtCPF1* KO2 cells led to the downregulation of N assimilation that further caused the upregulation of HSP and HSF.

**Figure 8:**
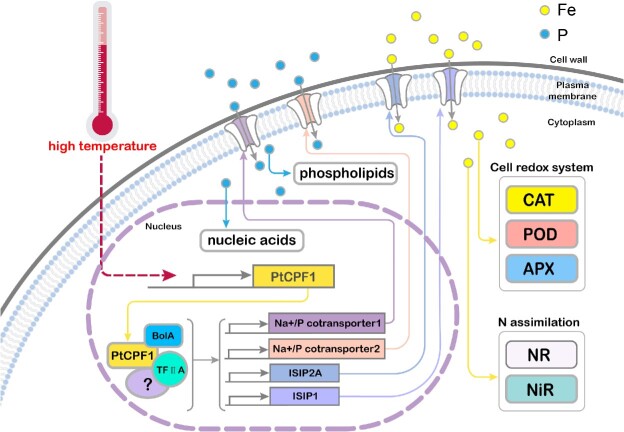
A hypothetical model for high temperature acclimation in marine diatoms via the uptake of Fe and P regulated by PtCPF1; at high temperature, the expression of *PtCPF1* increased, which interacted with BolA, TF IIA, and other proteins, and regulated the expression of genes encoding two phytotransferrins (ISIP2A and ISIP1) and two Na^+^/P co-transporters (PHATRDRAFT_47667 and PHATRDRAFT_40433), which are indicated as Na^+^/P co-transporter 1 and 2, respectively. Phytotransferrins and Na^+^/P co-transporters were required for the uptake of Fe and P, respectively. Fe was a chief component of the cell redox systems and acted as a cofactor of various antioxidant enzymes such as CA, POD, and APX, and was also required for NR and NiR; P was an essential component of key molecules such as phospholipids and nucleic acids that were required for cell division.

To date, the downstream signal transduction from PtCPF1 in *P. tricornutum* is unknown. Land plants respond to variable light and temperature conditions using common signal elements including phytochrome interacting factor 4 (PIF4, a TF) and constitutive photomorphogenic 1 (COP1, an E3 ubiquitin ligase) to coordinate their acclimation [14]. However, homologs of COP1 and PIF4 are absent from the genome of diatoms [36]. Recently, it has been reported that CryP, a cryptochrome in marine diatoms, interacted with BolA [36]. BolA-like proteins are widely conserved from prokaryotes to eukaryotes, and were first identified in *Escherichia coli* as morphogenes and later as TF [37, 38]. In *P. tricornutum*, five genes encoding proteins of the BolA-like family have been discovered [39]. In this study, PtCPF1 not only interacted with BolA, but also with TF IIA (Supplementary Table S7), which is also a general TF [40]. Bioinformatic predication analysis of target genes of BolA and TF IIA suggested that TF IIA could bind the two conserved regions in the promoters of the two Na^+^/P co-transporters and ISIP2A, whereas BolA could not (Supplementary Table S7). In fact, BolA in higher plants mainly functions as redox-regulated transcriptional regulators and factors regulating Fe–S cluster biogenesis, which may not bind directly to the promoters [41, 42]. Therefore, in marine diatoms, the possible role of the interaction between cryptochromes (PtCPF1 and CryP) and BolA was to regulate the redox state of cryptochromes. Based on the present results, a model (Fig. 8) was proposed in which, at high temperature, the expression of *PtCPF1* in *P. tricornutum* increases, which further regulates the expression of genes encoding two phytotransferrins (ISIP2A and ISIP1) and two Na^+^/P co-transporters (indicated as Na^+^/P co-transporter 1 and 2) by interacting with BolA, TF IIA, and other proteins. Phytotransferrins and Na^+^/P co-transporters are required for the uptake of Fe and P, respectively. As mentioned above, Fe is required for NR and NiR, and also acts as a cofactor of various antioxidant enzymes such as CAT, POD, and APX. Moreover, P is an essential component of key molecules such as phospholipids and nucleic acids, which are required for cell division.

In summary, PtCPF1 was found to regulate high temperature acclimation in marine diatoms through the coordination of Fe and P uptake, which played an irreplaceable role in alleviating stress. These findings not only explained the ability of marine diatoms to dominate in upwelling systems where the temperature is dynamic, but also demonstrated that the acquisition of Fe and P was essential for the acclimation of diatoms to marine environments, especially high temperature conditions.

## Materials and methods

### Cell culture conditions

Axenic *P. tricornutum* cells were obtained from the Institute of Hydrobiology, Chinese Academy of Sciences. As detailed in a subsequent section, *PtCPF1*-KO lines were generated. Additionally, a *PtCPF1* KO2-rescue and a His-tagged *PtCPF1* transgenic clone were also generated. The WT and manipulated lines of *P. tricornutum* cells were grown in F/2 medium comprising artificial seawater. Cells were grown at 20 °C in a 12 h light/12 h dark cycle under 80 μmol photons m^−2^ s^−1^. To investigate the responses of the WT and manipulated lines to high temperature, 26 °C was compared against a control of 20 °C. Each condition was set up in triplicate. Samples were taken daily for cell counting. The growth rate was estimated from cell concentration measurements using the equation μ = ln(N_t_–N_0_)/t from published paper [43] where μ is the growth rate, N_0_ is the starting cell concentration, N_t_ is the cell concentration at the end day of the exponential growth phase, and t is the end day of the exponential growth phase.

### Detection of *PtCPF1* in the *Tara* oceans dataset

A search for *PtCPF1* and *PtCPF1*-like genes was performed in the Marine Atlas of Tara Oceans Unigenes and eukaryote metatranscriptomes (MATOUva+metaT) datasets. The BLASTP-algorithm-based search was conducted using *P. tricornutum PtCPF1* (PHATRDRAFT_27429, NCBI Gene ID: 7201137) amino acid sequences from the National Center for Biotechnology Information as queries with an e-value ≤1e-10 as the threshold. The Marine Atlas of Tara Oceans Unigenes is a catalog of 116 million unigenes obtained from the eukaryote cDNA sequencing of samples with different size fractions from different water layers at various sampling stations; it is available on the Ocean Gene Atlas website (http://tara-oceans.mio.osupytheas.fr/ocean-gene-atlas/) [44].

The original data including *PtCPF1* homolog abundance in a geographic distribution map were downloaded, and two Excel files were obtained. The abundance matrix was the correspondence between the mRNA abundance of all *PtCPF1* homologs species that met the set parameters (0.8–2000 μm size fraction, 10–30 °C temperate range) and their sampling stations. To ensure the accuracy of the data, marine zooplankton data were removed, only marine phytoplankton data were selected. The information in the environmental parameters file was the correspondence between each sample and each environmental parameter at the sampling stations. Each sample ID and the corresponding temperature information were retained. A corresponding relationship between species, mRNA abundance, and temperature was established. The mRNA abundance of all species with homology to *PtCPF1* at the same temperature was summed-up to obtain the correspondence between temperature and mRNA abundance. Origin software (OriginLab, Massachusetts) was used for data visualization, resulting in a schematic diagram showing the variation of the target gene mRNA abundance with temperature.

### Generation of *PtCPF1* knockout mutants by CRISPR/Cas9

*PtCPF1* KO strains were constructed according to a previous method in which the Cas9 system was delivered into *P. tricornutum* via conjugation of plasmids from a bacterial donor cell [45, 46]. Briefly, the Cas9 target site of *PtCPF1* with the PAM signal (Supplementary Table S3) was designed using an online program (http://crispor.tefor.net/). The designed oligonucleotide corresponding to the predicted gRNA binding site and the complementary oligonucleotide were synthesized, and then phosphorylated and annealed. Using Golden Gate assembly (New England Biolabs, Massachusetts), cloned gRNA was inserted into the pPtGE35 plasmid obtained from Addgene (107999). The product from the Golden Gate reaction was transformed into Epi300 *E. coli* cells using heat shock. The properly cloned gRNA plasmid was then transformed into DH10B *E. coli* cells containing the pTA-Mob plasmid obtained from Dr Rahmi Lale (Norwegian University of Science and Technology, Trondheim, Norway).

Subsequently, according to a published protocol [46], the pPtGE35 plasmid containing gRNA inserts was transferred into *P. tricornutum* via conjugation from *E. coli*. Screening for *PtCPF1* KO in *P. tricornutum* induced by Cas9 was performed as previously described [46] with minor modifications. After 2–3 weeks, 10 *P. tricornutum* exconjugants were randomly selected, inoculated into liquid F/2 medium with 50 μg ml^−1^ zeocin (Invitrogen, Carlsbad, CA), and grown for 5 days. The *P. tricornutum* exconjugant cells were lysed and used as the template for PCR amplification of the gRNA target site using the specific primers shown in Supplementary Table S4. The PCR products were sent for Sanger sequencing to verify the deletion length. Subsequently, liquid culture medium of *P. tricornutum* exconjugants identified to be edited was diluted to 10^−4^, plated onto F/2 medium plates containing 50 μg ml^−1^ zeocin, and grown for 10–14 days to obtain subclones. Additionally, to obtain homozygous *PtCPF1* KO mutants, several rounds of screening by plating heterozygous clones onto plates containing 50 μg ml^−1^ zeocin were carried out. Subsequently, clones were transferred to liquid culture medium. Moreover, cells were lysed and used as the template for PCR amplification of the gRNA target site using the specific primers shown in Supplementary Table S4. The PCR products were sent for Sanger sequencing to verify the homozygous or heterozygous. In addition, curing of the pPtGE35 plasmid from the *PtCPF1* KO mutants was performed according to a previous method [46].

### Generation of *PtCPF1*-His_6_ line and rescue of *PtCPF1* knockout with native *PtCPF1*

To generate the *PtCPF1*-His_6_ line with a terminal His6-tag, the coding sequence of *PtCPF1* was amplified from cDNA of the WT using the primers (PtCPF1inoeF and PtCPF1inHisoeR) shown Supplementary Table S4. In addition, for *PtCPF1* KO mutant complementation with the native *PtCPF1*, the genomic sequence including the promoter and terminator of *PtCPF1* was PCR amplified from *P. tricornutum* genomic DNA using the primers (PtCPF1ptF and PtCPF1ptR) shown in Supplementary Table S4. The length of the promoter is 2000 bp upstream the ATG codon. The length of the terminator is 300 bp downstream the stop codon. The products were ligated into the p0521s expression vector obtained from Addgene (62862) by *pEASY*-Uni Seamless Cloning and Assembly Kit (TransGen Biotech Co., Ltd, Beijing). The properly cloned PtCPF1-His_6_ plasmid was then transformed into DH10B *E. coli* cells containing the pTA-Mob plasmid obtained from Dr Rahmi Lale. The subsequent protocols were performed according to the published protocol [46] mentioned above.

### Measurement of chlorophyll fluorescence and P700

In vivo chlorophyll *a* fluorescence and P700 oxidation signals were measured simultaneously at 20 °C in *P. tricornutum* WT and manipulated strains of *P. tricornutum* cells using a Dual-PAM-100 (Heinz Walz, Germany). Before measurements, cells were centrifuged (1500 × *g*, at 20 °C, 3 min) and concentrated 40-fold, and then dark-acclimated for 10 min. The detailed protocols were in accordance to a previous method [21]. The main photosynthetic parameters were Y(II), Y(I), rETR(II), and rETR(I). Y(II) and Y(I) mean the effective photochemical quantum yield of PSII and PSI, respectively. Moreover, rETR(II) and rETR(I) mean the relative electron transport rate of PSII and PSI, respectively.

### Measurement of *PtCPF1* expression in *P. tricornutum* using quantitative reverse transcription polymerase chain reaction

Cells were cultured under 12-h light/12-h dark periods, which were collected at different time points under light condition. Cells were harvested by centrifugation at 5000 × *g* for 3 min. Pellets were frozen in liquid nitrogen and ground in a pestle. Powdered cells were treated with the RNAprep Pure Plant Kit (Tiangen, Beijing, China). Reverse transcription of RNA was performed with the PrimeScript RT reagent kit (TaKaRa, Kyoto, Japan) following the recommended protocol. The qRT-PCR was performed as previously described [19]. The 30S ribosomal protein subunit was used as an internal control. The expression of the *PtCPF1* gene at different time points and temperatures was normalized to the starting point (0 h or 0 days) at 20 °C. The expression of *PtCPF1* gene in mutant at 20 °C or 26 °C was normalized to the WT at 20or 26 °C, respectively. One-way analysis of variance (ANOVA) was performed using SPSS 18 software (IBM, New York) to evaluate the significance of the estimated relative expression ratios. The forward and reverse primers are listed in Supplemental Table S5.

### Transcriptome profiling using mRNA-sequencing

WT and *PtCPF1* KO2 with a starting cell density of 3.5 × 10^5^ cells ml^−1^ were grown for 7 days at 20 and 26 °C. Because *PtCPF1* KO2 does not grow at 26 °C, the inoculum volume of *PtCPF1* KO2 was 2 l to obtain sufficient biomass for RNA extraction. Cells were harvested at 7 days for mRNA-sequencing. Three biological replicates of the WT and *PtCPF1* KO2 for each condition were processed for total RNA extraction using the TRIzol reagent kit (Invitrogen), according to the manufacturer’s protocol. The enriched mRNA was fragmented into short fragments and reverse transcribed into cDNA. The cDNA fragments were purified, end repaired, and a poly (A) tail was added. The fragments were ligated to Illumina sequencing adapters. The ligation products were size-selected using electrophoresis, PCR-amplified, and then sequenced using the Novaseq 6000 (Illumina) (Gene Denovo Biotechnology Co. Ltd, Guangzhou, China).

### Measurement of N, P, and Fe contents in the cultures

The N (NO_3_^−^) and P (PO_4_^3−^) contents in the cultures were measured using an *in situ* nutrient analyzer (KRL-Nutrients; Qingdao KenRuiLong Technology Co., Qingdao, China). In brief, after 7 days at 20 and 26 °C, 100 ml cultures from the WT and different mutants were centrifugated at 5000 × *g* for 3 min, and the supernatant was used to measure N and P with the model of NO_3_^−^ and PO_4_^3−^ in the analyzer. In addition, Fe contents in the cultures were measured using inductively coupled plasma mass spectrometry (Thermo Fisher Scientific, MA) according to a previous method [47]. The initial concentration of N, P, and Fe in F/2 culture medium was 2090 , 1149 , 578 μg/l, respectively, all of which were presented as the mean ± SD (*n* = 3 biologically independent experiments). The P uptake of different samples was calculated based on the published paper [43].

### Identification of PtCPF1 interaction proteins

For identification of the PtCPF1 interaction partner, a 1-l *PtCPF1*-His_6_ line culture grown for 7 days at 20 °C in 12-h light/12-h dark cycle under 80 μmol photons m^−2^ s^−1^ was dark-adapted for 60 h and then exposed for 1 h to 20 μmol photons m^−2^ s^−1^ of blue light. Cells were collected by centrifugation (5000 × *g*, 5 min, 4 °C) and ultrasonicated three times in phosphate-buffered saline. The solution was centrifuged (12 000 × *g*, 10 min, 4 °C), and the supernatant (total water-soluble protein) was applied to the following experiments. About 10 mg of the total water-soluble protein was applied to the immobilized metal chelate affinity chromatography Sepharose 6 Fast Flow (Sweden) for 2 h at 4 °C in darkness. After washing with three column volumes of washing buffer (20 mM KH_2_PO_4_, 500 mM NaCl, 20 mM imidazole, 5% glycerol, pH 7.0), an elution was carried out with 40% elution buffer (20 mM KH_2_PO_4_, 500 mM NaCl, 500 mM imidazole, 5% glycerol, pH 7.0). The elution proteins were ultrafiltrate with Amicon Ultra-15 (Millipore, 10 kDa) (4000 × *g*, 30 min, 4 °C).

To identify the PtCPF1 complex, the elution was separated by BN-PAGE (4%–16% Bis-Tris Gel) (Invitrogen) according to a previous method [48]. After electrophoresis, the BN gel was transferred to a polyvinylidene difluoride membrane (Millipore), and protein detection was carried out with His antibody. Additionally, the gels were stained with CBB G250 and silver. Three BN bands (BN1, BN2, and BN3) were indicated. To identify the components, the three bands were excised from the BN gel with CBB staining, and de-stained, washed, and in-gel digested with trypsin as described previously [48]. Subsequently, peptides were subjected to nano-LC-ESI-MS/MS using an Easy nLC 1200 nano HPLC apparatus (Thermo Fisher Scientific) with a Rx-C18 column (75 μm × 25 cm C18–2 μm 100 Å) coupled online with a Nano Flex ESI Q Exactive mass spectrometer (Thermo Fisher Scientific). Data were analyzed using PEAKS Studio 8.5 (Bioinformatics Solutions Inc., Waterloo, Canada). The *P. tricornutum* database downloaded from Uniprot was used.

### Statistical analysis

All statistical tests are described in the figure legends. Results were expressed as the mean ± standard deviation. The linear relationship between temperature and transcript abundance of PtCPF1 homologs were evaluated by regression analysis and Durbin–Watson test (*P* < .05) using SPSS 18.0 software. The comparisons between the mean of the two groups were evaluated using an independent sample *t*-test. Multiple comparisons between different groups were evaluated by ANOVA with Tukey’s multiple comparisons tests using SPSS 18.0 software. Correlation analysis was evaluated by Pearson and Spearman correlation using SPSS 18.0 software.

## Supplementary Material

20231201_Supplementary_figures_S1_wrad019

20231201_Supplementary_figures_S2_wrad019

20231201_Supplementary_figures_S3_wrad019

20231201_Supplementary_figures_S4_wrad019

20231201_Supplementary_figures_S5_wrad019

20231201_Supplementary_figures_S6_wrad019

20231201_Supplementary_figures_S7_wrad019

20231201_Supplementary_figures_S8_wrad019

20231201_Supplementary_figures_S9_wrad019

20231201_Supplementary_figures_S10_wrad019

20231201_Supplementary_figures_S11_wrad019

20231201_Supplementary_figures_S12_wrad019

20231201_Supplementary_figures_S13_wrad019

20231201_Supplementary_figures_S14_wrad019

20231201_Supplementary_tables_S1_wrad019

20231201_Supplementary_tables_S2_wrad019

20231201_Supplementary_tables_S3_wrad019

20231201_Supplementary_tables_S4_wrad019

20231201_Supplementary_tables_S5_wrad019

20231201_Supplementary_tables_S6_wrad019

20231201_Supplementary_tables_S7_wrad019

20231201_Supplementary_tables_S8_wrad019

20231201_Supplementary_tables_S9_wrad019

## Data Availability

Raw data of our transcriptome have been deposited in the China National Center for Bioinformation (BioProject accession number PRJCA021738). Raw data of LC–MS/MS have been deposited in the supplementary data.
