# Supplementary material for: Cryptochrome PtCPF1 regulates high temperature acclimation of marine diatoms through coordination of iron and phosphorus uptake
Source: ISME J. 2024 Jan 10;18(1):wrad019. doi: 10.1093/ismejo/wrad019 (PMC10837835; doi:10.1093/ismejo/wrad019)
Supplement: 20231201_Supplementary_figures_S1_wrad019 [file 20231201_supplementary_figures_s1_wrad019.pdf]

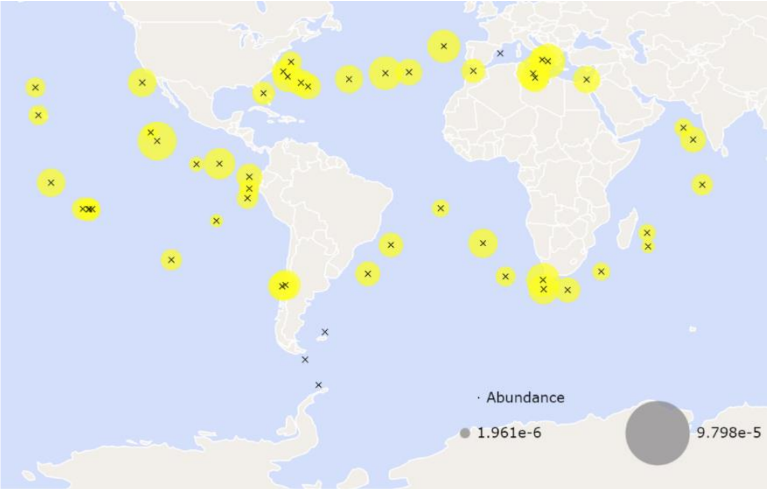

Figure S1 Wide geographic distribution of mRNA abundance of *PtCPF1* homologs in marine phytoplankton found in *Tara* Oceans. The size of cycles represents *PtCPF1* mRNA abundance. The abundance based on the 5-20 μm size fractions from surface layer and the range of temperature (10-30 ℃) from *Tara* Ocean datasets.
