# Supplementary material for: Cryptochrome PtCPF1 regulates high temperature acclimation of marine diatoms through coordination of iron and phosphorus uptake
Source: ISME J. 2024 Jan 10;18(1):wrad019. doi: 10.1093/ismejo/wrad019 (PMC10837835; doi:10.1093/ismejo/wrad019)
Supplement: 20231201_Supplementary_figures_S2_wrad019 [file 20231201_supplementary_figures_s2_wrad019.pdf]

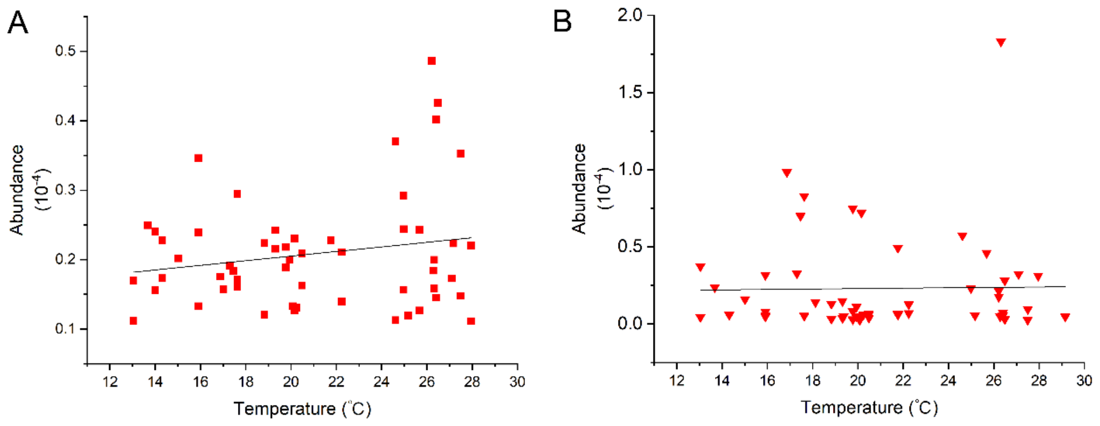

Figure S2 Analysis of the relationship between the abundance of *PtCPF1* mRNA and temperature in dinophyceae (A) and stramenopiles (B). The mRNA abundance of *PtCPF1* homologs in phytoplankton that meet the set parameters (0.8–2,000 μm, 10–30℃) and their sampling stations from *Tara* Ocean datasets. The unit for abundance is percent of total reads. The linear relationship between temperature and transcript abundance of PtCPF1 homologs were evaluated by regression analysis and Durbin-Watson test (p<0.05) in A.
