# Supplementary material for: Cryptochrome PtCPF1 regulates high temperature acclimation of marine diatoms through coordination of iron and phosphorus uptake
Source: ISME J. 2024 Jan 10;18(1):wrad019. doi: 10.1093/ismejo/wrad019 (PMC10837835; doi:10.1093/ismejo/wrad019)
Supplement: 20231201_Supplementary_figures_S3_wrad019 [file 20231201_supplementary_figures_s3_wrad019.pdf]

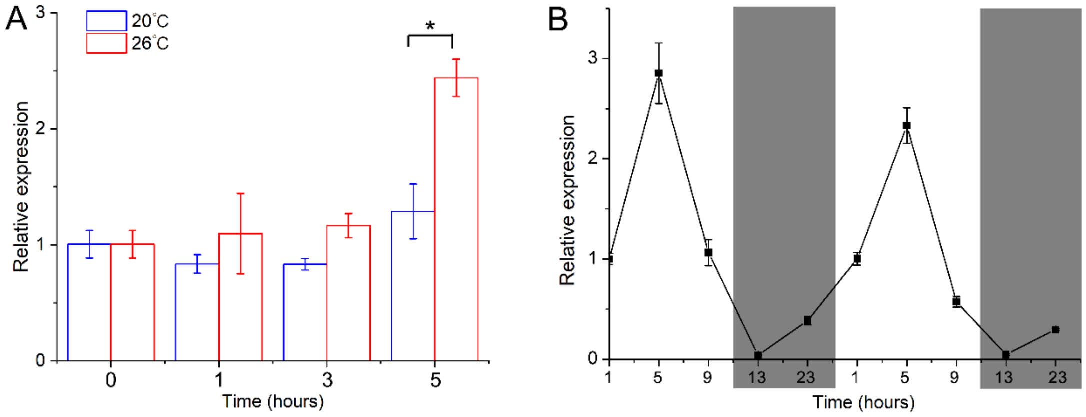

Figure S3 (A) The relative expression of *PtPCF1* in *Phaeodactylun tricornutum* with short-term treatment (0, 1, 3, and 5 h) of 20 and 26 ℃ under dark conditions determined by quantitative PCR with reverse transcription (quantitative RT-PCR). Error bars represent mean values ±SD (n=3 biologically independent experiments). Asterisk indicate that there was significant difference between the two groups. Independent samples T Test were used to compare the two groups ($p<$ 0.05). (B) The relative expression of *PtPCF1* in *P. tricornutum* under 12 h light/12 h dark photoperiod. Samples were collected 1, 5, 9, 13, 23 h after illumination over two days. qRT-PCR analysis of the PtCFP1 mRNAs in cells grown in 12 h light / 12 h dark for 2 days. Expression levels were normalized against *RPS*. The grey background represent dark period.
