# Supplementary material for: Cryptochrome PtCPF1 regulates high temperature acclimation of marine diatoms through coordination of iron and phosphorus uptake
Source: ISME J. 2024 Jan 10;18(1):wrad019. doi: 10.1093/ismejo/wrad019 (PMC10837835; doi:10.1093/ismejo/wrad019)
Supplement: 20231201_Supplementary_figures_S4_wrad019 [file 20231201_supplementary_figures_s4_wrad019.pdf]

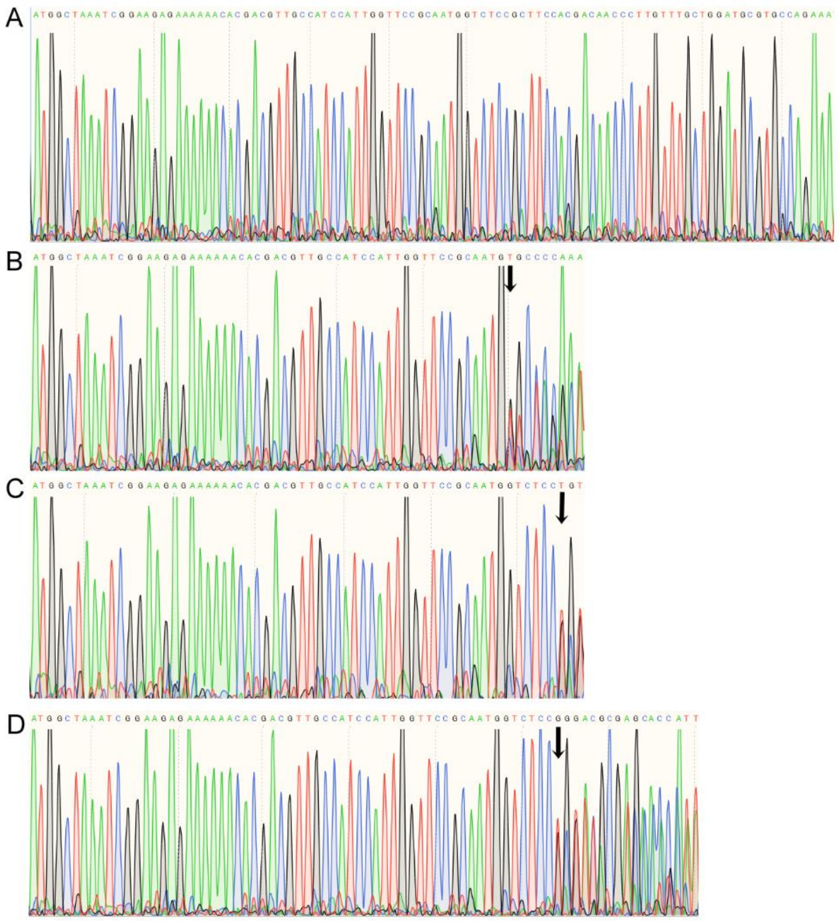

Figure S4 The Sanger sequencing of wild-type (A) and three heterozygous *PtCPF1* mutants [*PtCPF1* KO1 He (B), *PtCPF1* KO2 He (C) and *PtCPF1* KO3 He (D)]. 'He' represents heterozygous.
