# Supplementary material for: Cryptochrome PtCPF1 regulates high temperature acclimation of marine diatoms through coordination of iron and phosphorus uptake
Source: ISME J. 2024 Jan 10;18(1):wrad019. doi: 10.1093/ismejo/wrad019 (PMC10837835; doi:10.1093/ismejo/wrad019)
Supplement: 20231201_Supplementary_figures_S5_wrad019 [file 20231201_supplementary_figures_s5_wrad019.pdf]

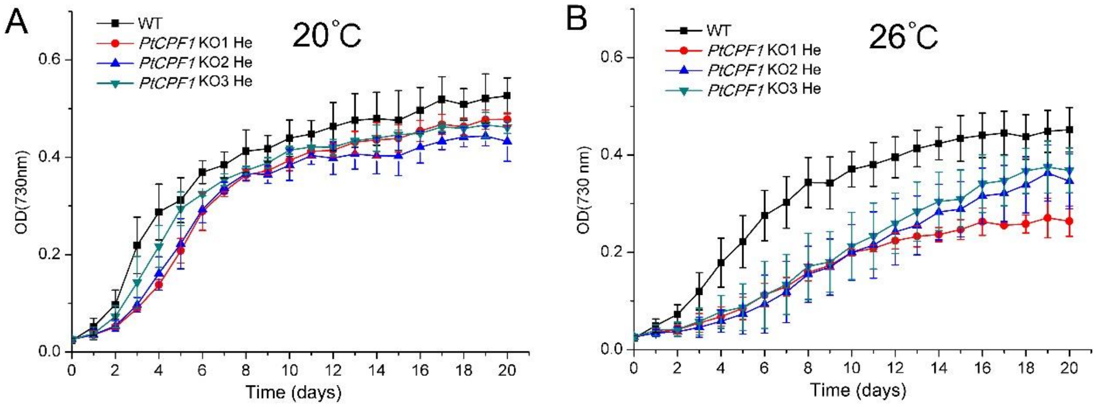

Figure S5 Growth curves of the three heterozygous *PtCPF1* mutants (*PtCPF1* KO1 He, *PtCPF1* KO2 He and *PtCPF1* KO3 He) and wild-type at 20 (A) and 26 ℃ (B) conditions. 'He' represents heterozygous. Samples were taken daily for cell counting. Data are presented as mean values ±SD (*n*= 3 biological independent experiments).
