# Supplementary material for: Cryptochrome PtCPF1 regulates high temperature acclimation of marine diatoms through coordination of iron and phosphorus uptake
Source: ISME J. 2024 Jan 10;18(1):wrad019. doi: 10.1093/ismejo/wrad019 (PMC10837835; doi:10.1093/ismejo/wrad019)
Supplement: 20231201_Supplementary_figures_S7_wrad019 [file 20231201_supplementary_figures_s7_wrad019.pdf]

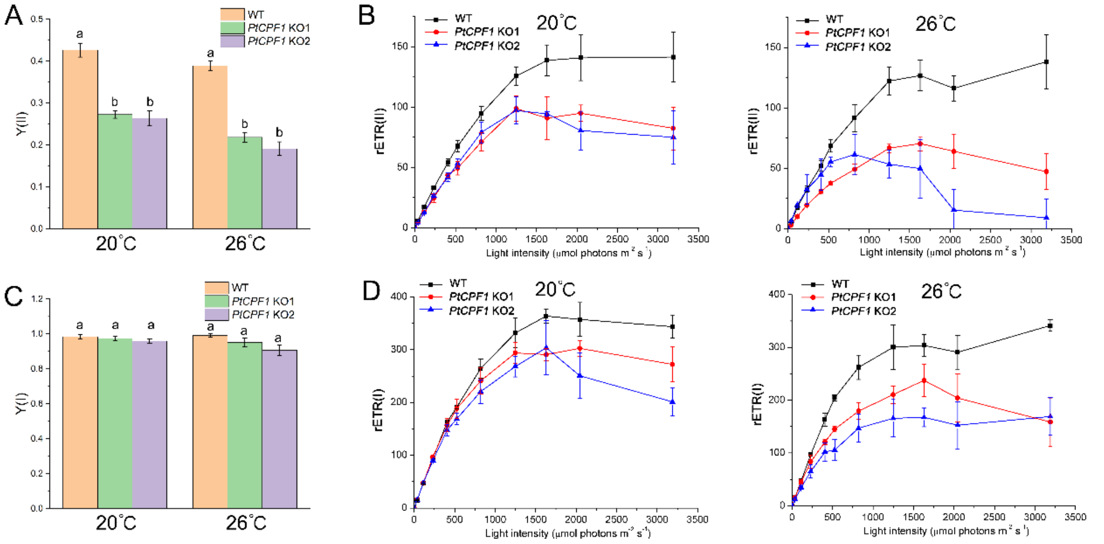

Figure S7 Photosynthetic activity of wild-type and two homozygous *PtCPF1* mutants (*PtCPF1* KO1 and *PtCPF1* KO2) at 20 and 26 ℃ conditions. Y(II) (A) and rETR(II) (B) of WT, *PtCPF1* KO1, and *PtCPF1* KO2 at different temperature conditions. Y(I) (C) and rETR(I) (D) of WT, *PtCPF1* KO1, and *PtCPF1* KO2 at different temperature conditions. Data are presented as the mean ±SD (n=3 biological independent experiments). Different lowercase letters indicate statistically significant differences, as determined by one-way ANOVA with Tukey’s multiple comparisons test (p<0.05).
