# Supplementary material for: Cryptochrome PtCPF1 regulates high temperature acclimation of marine diatoms through coordination of iron and phosphorus uptake
Source: ISME J. 2024 Jan 10;18(1):wrad019. doi: 10.1093/ismejo/wrad019 (PMC10837835; doi:10.1093/ismejo/wrad019)
Supplement: 20231201_Supplementary_figures_S8_wrad019 [file 20231201_supplementary_figures_s8_wrad019.pdf]

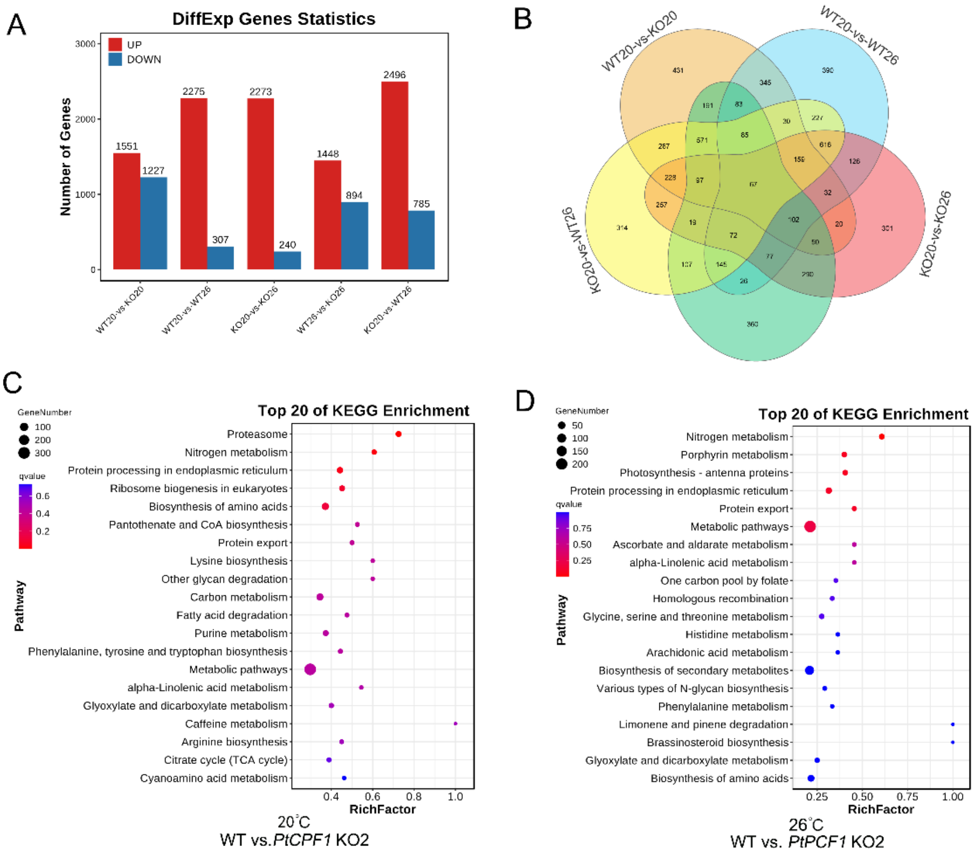

Figure S8 Transcriptomic profiling of wild-type and *PtCPF1* KO2 cells at 20 and 26 ℃ conditions. DEGs statistic (A) and venn diagram (B) between different comparisons. Top 20 of KEGG enrichment analysis of wild-type vs. *PtCPF1* KO2 at 20 (C) and 26 ℃ (D) conditions.
