# Supplementary material for: Cryptochrome PtCPF1 regulates high temperature acclimation of marine diatoms through coordination of iron and phosphorus uptake
Source: ISME J. 2024 Jan 10;18(1):wrad019. doi: 10.1093/ismejo/wrad019 (PMC10837835; doi:10.1093/ismejo/wrad019)
Supplement: 20231201_Supplementary_figures_S9_wrad019 [file 20231201_supplementary_figures_s9_wrad019.pdf]

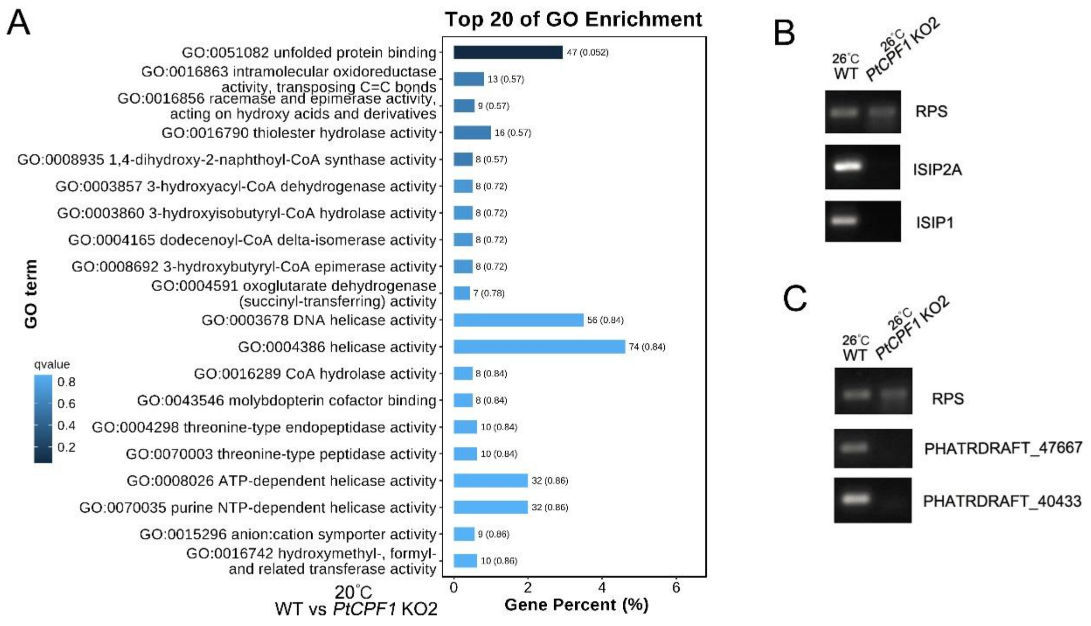

Figure S9 A GO enrichment analysis (molecular functions) of the wild type (WT)-vs-*PtCPF1* KO2 at 20 ℃. B and C, Agarose gel of PCR products from genomic DNA in wild-type and *PtCPF1* KO2 at 26 ℃ condition using the primers of qRT-PCR of the indicated genes with 28 cycles.
