# Supplementary material for: Cryptochrome PtCPF1 regulates high temperature acclimation of marine diatoms through coordination of iron and phosphorus uptake
Source: ISME J. 2024 Jan 10;18(1):wrad019. doi: 10.1093/ismejo/wrad019 (PMC10837835; doi:10.1093/ismejo/wrad019)
Supplement: 20231201_Supplementary_figures_S10_wrad019 [file 20231201_supplementary_figures_s10_wrad019.pdf]

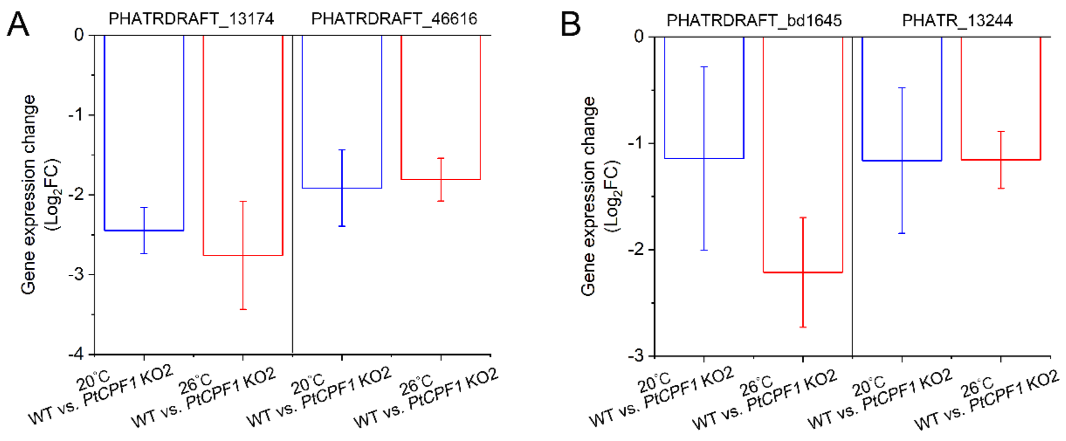

Figure S10

Expression patterns of genes encoding two ascorbate peroxidases (PHATRDRAFT_13174 and PHATRDRAFT_46616) (A), peroxidase domain-containing protein (PHATRDRAFT_bd1645) and catalase (PHATR_13244) (B) of *PtCPF1* KO2 mutant at 20 ℃ and 26 ℃. FC, fold change. Data are presented as mean values ±SD (*n*= 3 biological independent experiments).
