# Supplementary material for: Cryptochrome PtCPF1 regulates high temperature acclimation of marine diatoms through coordination of iron and phosphorus uptake
Source: ISME J. 2024 Jan 10;18(1):wrad019. doi: 10.1093/ismejo/wrad019 (PMC10837835; doi:10.1093/ismejo/wrad019)
Supplement: 20231201_Supplementary_figures_S11_wrad019 [file 20231201_supplementary_figures_s11_wrad019.pdf]

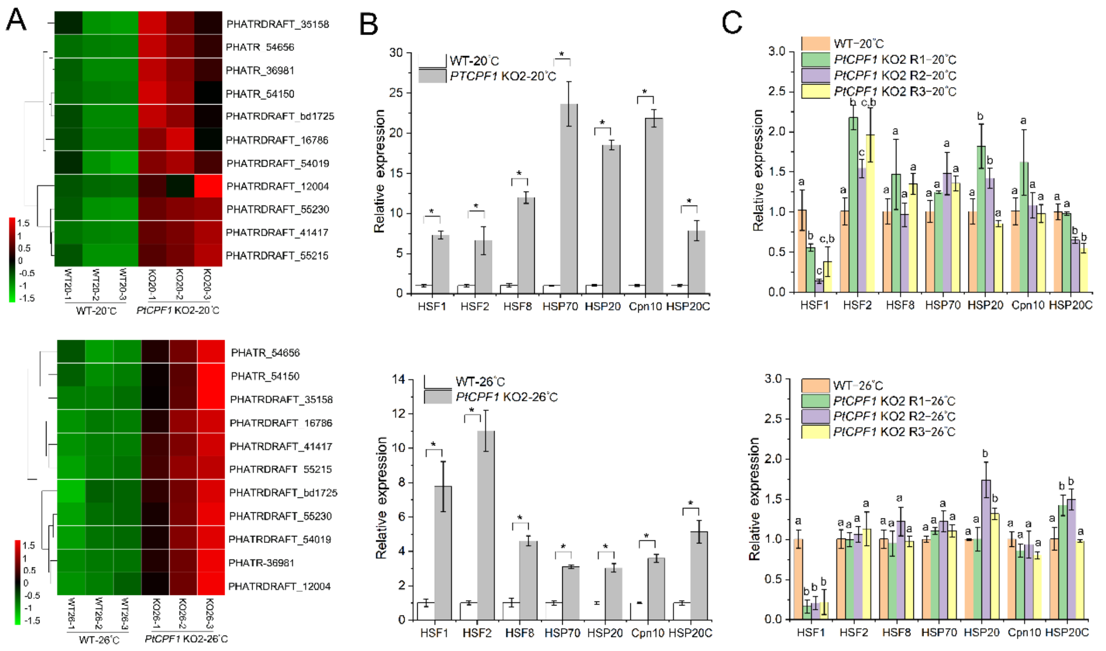

Figure S11 PtCPF1 affects the expression of genes encoded HSP and HSF. A, Heat maps showing the relative expression changes of genes encoded HSP at indicated conditions. B, The relative expression of major HSP (Heat Shock Protein) and HSF (Heat Shock Factor) genes in the wild-type and the *PtCPF1* KO2 at indicated conditions as determined by qRT-PCR. Asterisks indicate a significant difference between two groups. Independent samples *t*-tests were used to compare the two groups (p<0.05). C, The relative expression of major HSP and HSF genes in the wild-type and the three *PtCPF1* KO2 rescued lines under indicated conditions as determined by qRT-PCR. Error bars represent SD (n=3 biologically independent experiments). HSF1, PHATRDRAFT_39785; HSF2, PHATRDRAFT_47952; HSF8, PHATRDRAFT_45206; HSP70, PHATRDRAFT_41417; HSP20, PHATR_54656; Cpn10, PHATRDRAFT_12004; HSP20C, PHATR_36981. Different lowercase letters indicate statistically significant differences, as determined by one-way ANOVA with Tukey's multiple comparisons test (p<0.05).
