# Supplementary material for: Cryptochrome PtCPF1 regulates high temperature acclimation of marine diatoms through coordination of iron and phosphorus uptake
Source: ISME J. 2024 Jan 10;18(1):wrad019. doi: 10.1093/ismejo/wrad019 (PMC10837835; doi:10.1093/ismejo/wrad019)
Supplement: 20231201_Supplementary_figures_S12_wrad019 [file 20231201_supplementary_figures_s12_wrad019.pdf]

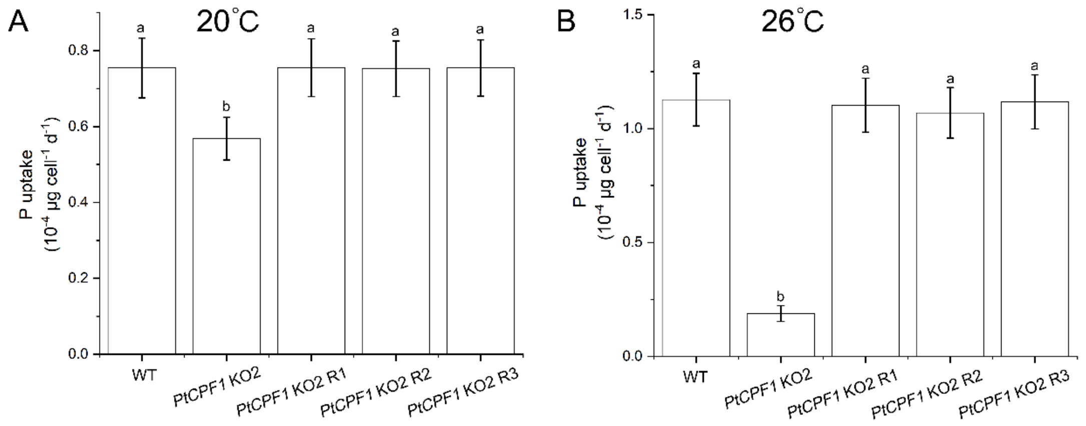

Figure S12 P uptake of the wild-type, *PtCPF1* KO2, and the rescued lines (*PtCPF1* KO2 R1, R2, and R3) at 20 ℃and 26℃. Different lowercase letters indicate statistically significant differences, as determined by one-way ANOVA with Tukey's multiple comparisons test (p<0.05).
