# Supplementary material for: Cryptochrome PtCPF1 regulates high temperature acclimation of marine diatoms through coordination of iron and phosphorus uptake
Source: ISME J. 2024 Jan 10;18(1):wrad019. doi: 10.1093/ismejo/wrad019 (PMC10837835; doi:10.1093/ismejo/wrad019)
Supplement: 20231201_Supplementary_figures_S13_wrad019 [file 20231201_supplementary_figures_s13_wrad019.pdf]

A

Descriptive Statistics

	N	Mean	SD	Sum	Min	Max
"Growth rate-20"	8	1.79595	0.28566	14.36761	1.5253	2.07035
"P-20"	8	218.39375	214.7586	1747.15	14.2338	431.4698

Pearson Correlations

		Growth rate-20	P-20
"Growth rate-20"	Pearson Corr.	1	-0.9991
	p-value	--	1.7983E-9
"P-20"	Pearson Corr.	-0.9991	1
	p-value	1.7983E-9	--

2-tailed test of significance is used

Spearman Correlations

		Growth rate-20	P-20
"Growth rate-20"	Spearman Corr.	1	-0.6386
	p-value	--	0.08833
"P-20"	Spearman Corr.	-0.6386	1
	p-value	0.08833	--

2-tailed test of significance is used

20°C

B

Descriptive Statistics

	N	Mean	SD	Sum	Min	Max
"Growth rate-20"	8	1.79595	0.28566	14.36761	1.5253	2.07035
"Fe-20"	8	175.2275	7.88452	1401.82	164	183.65

Pearson Correlations

		Growth rate-20	Fe-20
"Growth rate-20"	Pearson Corr.	1	-0.94994
	p-value	--	3.01993E-4
"Fe-20"	Pearson Corr.	-0.94994	1
	p-value	3.01993E-4	--

2-tailed test of significance is used

Spearman Correlations

		Growth rate-20	Fe-20
"Growth rate-20"	Spearman Corr.	1	-0.59756
	p-value	--	0.11772
"Fe-20"	Spearman Corr.	-0.59756	1
	p-value	0.11772	--

2-tailed test of significance is used

20°C

C

Descriptive Statistics

	N	Mean	SD	Sum	Min	Max
"Growth rate-20"	8	1.79595	0.28566	14.36761	1.5253	2.07035
"N-20"	8	2023.32618	65.01875	16186.60947	1895.7205	2086.8274

Pearson Correlations

		Growth rate-20	N-20
"Growth rate-20"	Pearson Corr.	1	-0.31715
	p-value	--	0.44402
"N-20"	Pearson Corr.	-0.31715	1
	p-value	0.44402	--

2-tailed test of significance is used

Spearman Correlations

		Growth rate-20	N-20
"Growth rate-20"	Spearman Corr.	1	-0.31328
	p-value	--	0.44991
"N-20"	Spearman Corr.	-0.31328	1
	p-value	0.44991	--

2-tailed test of significance is used

20°C

D

Descriptive Statistics

	N	Mean	SD	Sum	Min	Max
"Growth rate-26"	8	1.12282	1.20035	8.9826	0	2.2477
"P-26"	8	528.45512	542.57118	4227.64093	13.1093	1089.108

Pearson Correlations

		Growth rate-26	P-26
"Growth rate-26"	Pearson Corr.	1	-0.99782
	p-value	--	2.5809E-8
"P-26"	Pearson Corr.	-0.99782	1
	p-value	2.5809E-8	--

2-tailed test of significance is used

Spearman Correlations

		Growth rate-26	P-26
"Growth rate-26"	Spearman Corr.	1	-0.83712
	p-value	--	0.00953
"P-26"	Spearman Corr.	-0.83712	1
	p-value	0.00953	--

2-tailed test of significance is used

26°C

E

Descriptive Statistics

	N	Mean	SD	Sum	Min	Max
"Growth rate-26"	8	1.12282	1.20035	8.9826	0	2.2477
"Fe-26"	8	197.7125	15.00167	1581.7	182.69	216.81

Pearson Correlations

		Growth rate-26	Fe-26
"Growth rate-26"	Pearson Corr.	1	-0.98076
	p-value	--	1.7543E-5
"Fe-26"	Pearson Corr.	-0.98076	1
	p-value	1.7543E-5	--

2-tailed test of significance is used

Spearman Correlations

		Growth rate-26	Fe-26
"Growth rate-26"	Spearman Corr.	1	-0.89862
	p-value	--	0.00241
"Fe-26"	Spearman Corr.	-0.89862	1
	p-value	0.00241	--

2-tailed test of significance is used

26°C

F

Descriptive Statistics

	N	Mean	SD	Sum	Min	Max
"Growth rate-26"	8	1.12282	1.20035	8.9826	0	2.2477
"N-26"	8	2057.71236	35.3292	16461.69887	1982.5909	2090.5676

Pearson Correlations

		Growth rate-26	N-26
"Growth rate-26"	Pearson Corr.	1	-0.71115
	p-value	--	0.04795
"N-26"	Pearson Corr.	-0.71115	1
	p-value	0.04795	--

2-tailed test of significance is used

Spearman Correlations

		Growth rate-26	N-26
"Growth rate-26"	Spearman Corr.	1	-0.83712
	p-value	--	0.00953
"N-26"	Spearman Corr.	-0.83712	1
	p-value	0.00953	--

2-tailed test of significance is used

26°C

Figure S13 Correlation analysis between nutrient concentration and growth rate of WT and *PtCPF1* KO2 at 20 and 26°C. At 20°C (A, B, and C), the correlation between nutrient concentration [A (P), B (Fe), and C (N)] and growth rate of WT and *PtCPF1* KO2 was not significant. At 26°C (D, E, and F), all nutrient concentration [D (P), E (Fe), and F (N)] and growth rate were of WT and *PtCPF1* KO2 significant (p<0.05). The analysis were evaluated by Pearson and Spearman correlation using Origin Pro 2022 software.
