# Supplementary material for: Cryptochrome PtCPF1 regulates high temperature acclimation of marine diatoms through coordination of iron and phosphorus uptake
Source: ISME J. 2024 Jan 10;18(1):wrad019. doi: 10.1093/ismejo/wrad019 (PMC10837835; doi:10.1093/ismejo/wrad019)
Supplement: 20231201_Supplementary_figures_S14_wrad019 [file 20231201_supplementary_figures_s14_wrad019.pdf]

A

Hit ID	Description	Score	E-Value
Mapoly0051s0029.1.p	Marchantia polymorpha Trihelix family protein	133	3e-09
Solyc09g090830.2.1	Solanum lycopersicum GRAS family protein	122	1e-07

B

Hit ID	Description	Score	E-Value
OPUNC06G18860.1	Oryza punctata G2-like family protein	149	1e-08
KN540621.1_FGP002	Oryza longistaminata G2-like family protein	137	4e-07
Kalax.0054s0081.2.p	Kalanchoe marnieriana bHLH family protein	107	0.001
Kalax.0216s0040.4.p	Kalanchoe marnieriana bHLH family protein	106	0.001
Kalax.0041s0137.2.p	Kalanchoe marnieriana bHLH family protein	106	0.001
Kaladp0068s0096.2.p	Kalanchoe laxiflora bHLH family protein	106	0.002
Kaladp0068s0096.5.p	Kalanchoe laxiflora bHLH family protein	106	0.002
Kalax.0216s0040.3.p	Kalanchoe marnieriana bHLH family protein	105	0.002
PH01000845G0410	Phyllostachys heterocycla ARF family protein	106	0.002
Kaladp0068s0096.6.p	Kalanchoe laxiflora bHLH family protein	105	0.002
Kalax.1691s0002.2.p	Kalanchoe marnieriana bHLH family protein	101	0.005

Figure S14 BLAST of BolA (PHATRDRAFT_14849) (A) and TF IIA (PHATRDRAFT_42776) (B) in Plant TF database (http://planttfdb.gao-lab.org/).
