# Supplementary material for: Cryptochrome PtCPF1 regulates high temperature acclimation of marine diatoms through coordination of iron and phosphorus uptake
Source: ISME J. 2024 Jan 10;18(1):wrad019. doi: 10.1093/ismejo/wrad019 (PMC10837835; doi:10.1093/ismejo/wrad019)
Supplement: 20231201_Supplementary_tables_S1_wrad019 [file 20231201_supplementary_tables_s1_wrad019.pdf]

**Table S1**

Effects of environmental variables (temperature, salinity, PAR, and $NO_3^-$) based on *Tara* Ocean dataset on the abundance of *PtCPF1* homologs transcripts. Pairwise comparisons of environmental variables are shown. The Pearson's correlation coefficient and p values are also shown.

**Correlations**

| | | abundance | temperature | salinity | PAR | NO3 |
|---|---|---|---|---|---|---|
| abundance | Pearson Correlation | 1 | .390** | -.416** | .153 | .488** |
| | Sig. (2-tailed) | | .001 | .000 | .215 | .000 |
| | N | 67 | 67 | 67 | 67 | 67 |
| temperature | Pearson Correlation | .390** | 1 | -.156 | .625** | .082 |
| | Sig. (2-tailed) | .001 | | .207 | .000 | .509 |
| | N | 67 | 67 | 67 | 67 | 67 |
| salinity | Pearson Correlation | -.416** | -.156 | 1 | .125 | -.084 |
| | Sig. (2-tailed) | .000 | .207 | | .313 | .499 |
| | N | 67 | 67 | 67 | 67 | 67 |
| PAR | Pearson Correlation | .153 | .625** | .125 | 1 | -.018 |
| | Sig. (2-tailed) | .215 | .000 | .313 | | .886 |
| | N | 67 | 67 | 67 | 67 | 67 |
| NO3 | Pearson Correlation | .488** | .082 | -.084 | -.018 | 1 |
| | Sig. (2-tailed) | .000 | .509 | .499 | .886 | |
| | N | 67 | 67 | 67 | 67 | 67 |

**. Correlation is significant at the 0.01 level (2-tailed).
