# Supplementary material for: Cryptochrome PtCPF1 regulates high temperature acclimation of marine diatoms through coordination of iron and phosphorus uptake
Source: ISME J. 2024 Jan 10;18(1):wrad019. doi: 10.1093/ismejo/wrad019 (PMC10837835; doi:10.1093/ismejo/wrad019)
Supplement: 20231201_Supplementary_tables_S2_wrad019 [file 20231201_supplementary_tables_s2_wrad019.pdf]

Table S2

Effects of environmental variables (temperature, salinity, PAR, and NO_3^-) based on *Tara* Ocean dataset on the abundance of *PtCPF1* homologs transcripts. Pairwise comparisons of environmental variables are shown. The Spearman's correlation coefficient and p values are also shown.

Correlations

			abundance	temperature	salinity	PAR	NO3
Spearman's rho	abundance	Correlation Coefficient	1.000	.366**	-.435**	.272*	.260*
		Sig. (2-tailed)	.	.002	.000	.026	.034
		N	67	67	67	67	67
	temperature	Correlation Coefficient	.366**	1.000	-.012	.637**	-.079
		Sig. (2-tailed)	.002	.	.921	.000	.526
		N	67	67	67	67	67
	salinity	Correlation Coefficient	-.435**	-.012	1.000	-.016	-.211
		Sig. (2-tailed)	.000	.921	.	.900	.087
		N	67	67	67	67	67
	PAR	Correlation Coefficient	.272*	.637**	-.016	1.000	-.221
		Sig. (2-tailed)	.026	.000	.900	.	.072
		N	67	67	67	67	67
	NO3	Correlation Coefficient	.260*	-.079	-.211	-.221	1.000
		Sig. (2-tailed)	.034	.526	.087	.072	.
		N	67	67	67	67	67

**. Correlation is significant at the 0.01 level (2-tailed).

*. Correlation is significant at the 0.05 level (2-tailed).
