# Supplementary material for: Cryptochrome PtCPF1 regulates high temperature acclimation of marine diatoms through coordination of iron and phosphorus uptake
Source: ISME J. 2024 Jan 10;18(1):wrad019. doi: 10.1093/ismejo/wrad019 (PMC10837835; doi:10.1093/ismejo/wrad019)
Supplement: 20231201_Supplementary_tables_S3_wrad019 [file 20231201_supplementary_tables_s3_wrad019.pdf]

Table S3

Sequences of gRNAs targeting the *PtCPF1* gene in *Phaeodactylum tricornutum* in this study. The predicted gRNA binding site is the underlined sequence. The complementary oligonucleotide sequence is also provided in the table. The oligonucleotides were designed with 5'-TCGA-3' as the top strand and 3'-CAAA-5' as the bottom strand. They serve as *Bsa*I restriction cut site overhangs to facilitate cloning into the Cas9 vector.

**gRNA number**	**Annealed oligos for cloning**
PtCPF1gRNA	5'-TCGAAACAAGGGTTGTCGTGGAAG -3'
	3'- TTGTTCCCAACAGCACCTTCCAAA-5'
