# Supplementary material for: Cryptochrome PtCPF1 regulates high temperature acclimation of marine diatoms through coordination of iron and phosphorus uptake
Source: ISME J. 2024 Jan 10;18(1):wrad019. doi: 10.1093/ismejo/wrad019 (PMC10837835; doi:10.1093/ismejo/wrad019)
Supplement: 20231201_Supplementary_tables_S4_wrad019 [file 20231201_supplementary_tables_s4_wrad019.pdf]

Table S4

List of the primers for *PtCPF1* gene and the reference gene, *ribosomal protein subunit* (*RPS*).

**Primer name**	**Sequence**	**Use**
PtCPF1gF1	GTCACAGTCAGCCCAATCCA	Amplifying the on-target site
PtCPF1gR1	CCAGCAAACAAGGGTTGTCG	
PtCPF1qF	CTTCCACGACAACCCTT	RT-PCR
PtCPF1qR	TTCCCGAGAACAACGAC	RT-PCR
RPSqF	GTGCAAGAGACCGGACATACC	RT-PCR
RPSqR	GTGCAAGAGACCGGACATACC	RT-PCR
PtCPF1inoeF	gtctgccgtttcgagaattc ATGGCTAAATCGGAAGAGAAAA	Amplifying the CDS of PtCPF1 with His_6-tag
PtCPF1inHisoeR	atagcacgcttctgaagcttTTA GTGGTGGTGGTGGTGGTG GTTGCGACGTTGTCGCTTT	
PtCPF1ptF	tcctaaggtagcgaaTGCGAATGTTTGTGTT GACTCGC	Amplifying the sequence including the promoter and terminator of native *PtCPF1*
PtCPF1ptR	atccctagcgtaactATTTCTTCTCGGAAAA GGTATTAGGGC	
p0521sF	GACACTTTCAGTGAGGACAAGAAG	Amplifying the sequence of native *PtCPF1* in the p0521s vector
p0521sR	CACCCGCTCGCGGGTGGGCCTACT	
