# Supplementary material for: Cryptochrome PtCPF1 regulates high temperature acclimation of marine diatoms through coordination of iron and phosphorus uptake
Source: ISME J. 2024 Jan 10;18(1):wrad019. doi: 10.1093/ismejo/wrad019 (PMC10837835; doi:10.1093/ismejo/wrad019)
Supplement: 20231201_Supplementary_tables_S5_wrad019 [file 20231201_supplementary_tables_s5_wrad019.pdf]

Table S5

List of *P. tricornutum* genes analyzed in qRT-PCR. ID number in the *P. tricornutum* genome and the primer sequences are indicated

ID	Description	Sequence(5'-3')
54465	ISIP2A	F: TTCGCTGCTGCTGTTCTTCCC<br>R: CAAGCCCGACATTTGCTCCATC
55031	ISIP1	F: TGATGGAAGACGGACTTGGTGG<br>R: CGTTTCCCGAAGCAAGGACAAT
47667	Na^+/P cotransporter	F: GGCGACGACACGAATCCCA<br>R: TCCTTGGCGGACGGAGACA
40433	Na^+/P cotransporter	F: TGGCGTGGGTTCCCGCTTGT<br>R: GACCCTTGCTGCCGTCATCG
54101	nitrate transporter, NRT	F: TGGCATCACCAAACAGGA<br>R: AGGAATAGAAGCGAACGA
8155	nitrite reductase D, NirD	F: AAACGTCGGCTCTTCG<br>R: CGGCTCCGTCGTCTAA
26029	nitrate transporter 2.4, NRT2.4	F: TGGAGTCGAATTGACCATGA<br>R: TGGCATTAGCCTTGTCACTG
13154	nitrite reductase B, NirB	F: CGAGAACCTTCCTGCCTTTA<br>R: TTGGTGCCCGCTTTGT
54983	nitrate reductase, NR	F: CCGTCTCATCATTCCTGGTT<br>R: GGCCGAATTGATGTTGAGTT
27757	nitrite and sulphite reductase, NIR_SIR	F: TTTCGGGATGAATCAGAGC<br>R: TGCCGTCTTGTGGTTGTT
51092	glutamine synthetase 2, GLN2	F: TTTGAATCCAGGGAAGACG<br>R: CGAAGTTATTTTCGGGTCCA
39785	Heat shock factor 1, HSF1	F: ATGGGTGGCAAGAACAATG<br>R: TTGAACCAGTTGGGAAGGAC
47952	Heat shock factor 2, HSF2	F: CGTGGTATTTCGCAAGTC<br>R: AAGAAAGGAGGAGTGATGAG
45206	Heat shock factor 8, HSF8	F: ACTTAGCCAGGCAGGGTT<br>R: ACGACTTGCGTGGGTATT
41417	Heat shock protein 70, HSP70	F: CGCTACCAAACTGAAGGAAT<br>R: TGCTCTGGACACGCTGAC
54656	Heat shock protein 20, HSP20	F: TGCCTTCGCAGTCAAACA<br>R: CAGGGACTTCCATTGTCATTTC
12004	chaperonin 10, Cpn10	F: CGAGGGAGAAGTCGTTGC<br>R: CCAGTTCTTCGTCGTCAATCT
36981	Heat shock protein 20C, HSP20C	F: ACTTGCTATTGCCGTTCC<br>R: ATGTTTGCTCACCGCTTT
