# Supplementary material for: Cryptochrome PtCPF1 regulates high temperature acclimation of marine diatoms through coordination of iron and phosphorus uptake
Source: ISME J. 2024 Jan 10;18(1):wrad019. doi: 10.1093/ismejo/wrad019 (PMC10837835; doi:10.1093/ismejo/wrad019)
Supplement: 20231201_Supplementary_tables_S6_wrad019 [file 20231201_supplementary_tables_s6_wrad019.pdf]

Table S6 The three bands were excised from the BN gel with CBB staining (Figure 7) and analyzed by LC ESI MS/MS analysis.

Accessior	Gene	Descriptic	Mw(kDa)	Length	Protein gr	Peptides I	Unique pe	Sequence
B7GCN5	PHATRDR	Agmatinas	45.337	416		44	44	71.9
Q9TK52	rbcL	Ribulose b	54.007	490		10	10	24.7
B7GBH2	P5CS	Delta-1-pyı	78.903	747	B7FRT7	12	12	17.4
B7G889	PHATRDR	Predicted ţ	101.145	930		22	22	33.8
B7G5H7	UBI1/2	Ubiquitin e	14.454	128	B7FY02;B	9	9	39.8
B7FZ64	CPF1	Cryptochrc	62.963	550		14	14	27.6
B7FTY0	PHATRDR	Predicted ţ	54.535	486		24	24	51.2
B7G5Z8	GDCP	Glycine cle	108.412	1005		20	20	29.4
B7FWA5	PHATRDR	Predicted ţ	16.342	148		6	6	43.2
B7G0H9	OAT	Ornithine a	48.685	448		10	10	27
B7G9N5	PHATRDR	Predicted ţ	38.493	345		13	13	40.6
B7G3E9	PHATRDR	Predicted ţ	40.846	365		14	14	45.5
B7FWM9	PHATRDR	Predicted ţ	37.19	346		11	11	43.9
B7GCX7	PHATRDR	Nicotinate-	31.919	294		2	2	6.1
B7GA60	PHATRDR	Predicted ţ	189.15	1731		1	1	0.5
B7G836	TPS2	Bifunctiona	132.567	1199		15	15	14.4
B7G4T8	OGD1	Oxoglutara	121.13	1073	B7G2I0	6	6	8.3
B7FZK6	PHATRDR	Predicted ţ	36.878	331		10	10	37.8
B7GEG8	PHATRDR	CPS III, ca	160.758	1463		1	1	1
B7FNU0	PHATRDR	Carbonic a	31.065	282		11	8	39.4
B7FXL7	PHATRDR	Predicted ţ	33.632	297		12	12	35.4
B7G7W5	PHATRDR	Urease OS	94.033	878		11	11	13.8
B7FRA9	PHATRDR	Predicted ţ	103.223	980		3	3	4.1
B7FVT3	PHATRDR	Alpha subu	81.461	712		13	13	18.3
B7FXS9	PHATRDR	Predicted ţ	37.79	350	B7GDC2	10	6	28.9
B7G5G4	PHATRDR	Phosphogl	46.654	441		5	5	15.6
B7G7S4	ACC1	Acetyl-Co/	229.514	2092		1	1	0.5
B7GAE9	PHATRDR	Predicted ţ	82.458	743		13	13	22.9
B5Y4Y9	PHATR_1	Catalase-p	82.467	736		17	17	27.6
B5Y589	PHATR_8	Predicted ţ	50.92	467		5	5	15
B7FR80	NDK3	Nucleoside	23.663	217		6	6	33.2
B7FUB7	BiP	ER luminal	72.266	659		12	9	20.8
B7GA21	PHATRDR	Predicted ţ	16.914	154		6	6	45.5
B5Y5B5	PHATR_4	Predicted ţ	28.432	248		7	7	30.6
B7FT40	PHATRDR	Predicted ţ	36.649	330		11	11	41.5
B7S3X2	PHATRDR	Predicted ţ	68.481	607		8	8	14.2
B7FUM7	PHATRDR	Adenylosu	57.143	526		7	7	15.2
B7G3K3	PHATRDR	Predicted ţ	58.717	528		9	9	28.6
B7G961	PK5	Pyruvate k	60.382	554		5	5	11.6
B7GBG6	PHATRDR	Predicted ţ	74.189	686		8	8	14.9
B7FTW1	PHATRDR	Aspartate-	44.288	387		7	7	20.2
B7G878	ACT1	Actin/actin	41.791	377	B7G5C0	8	8	23.3
B7FQ66	SHMT2	Serine hyd	54.921	501		5	5	10.4
B7G2E8	PHATRDR	Predicted ţ	35.236	311		3	3	6.1
B7GB21	PHATRDR	Predicted ţ	41.633	373		5	5	16.1
B7GCL1	PHATRDR	Glutathion	82.097	735		4	4	6.5
A0T0F1	atpA	ATP synth	54.621	505		7	6	20.8
B7FUU0	Tkl	Transketol	77.136	711		8	8	16.7
B7FVS4	PHATRDR	Predicted ţ	75.138	686		1	1	3.1
B7G5X2	CAT2	Carnitine c	77.333	688		5	5	10.8

B7G663	PHATRDR	Predicted p	66.396	625		2	2	4.6
B7GBI0	PHATRDR	Predicted p	68.622	634		7	7	12.1
B7FS46	atpB	ATP synth	53.619	501		7	5	19
B7G012	PHATRDR	Predicted p	34.223	317		4	4	20.2
B7FP49	Sec23	Protein tra	83.944	759		8	8	14.4
B7FQ84	HSP70A	Protein hea	70.962	653		4	4	9.3
B7FUT7	PHATRDR	Predicted p	29.656	281		3	3	13.9
B7FVF4	PHATRDR	Predicted p	74.006	699		2	2	2.9
B7FYJ4	PHATRDR	Predicted p	74.62	686		2	2	5
B7G3Y2	Hsp70_2	Predicted p	79.67	732		9	6	15.2
B7G6J3	PHATRDR	Predicted p	42.622	368		4	4	11.4
B7G7T4	PHATRDR	Predicted p	61.695	552		4	4	9.1
B7GB64	PHATRDR	Alanine gly	50.697	465		6	6	17.2
B5Y4G7	PHATR_4	Vacuolar p	184.114	1712		3	3	1.9
B7FUD1	PHATRDR	Predicted p	38.347	340		3	3	9.1
B7FUP3	PHATRDR	Amino-acid	52.967	473		3	3	7.2
B7G091	PHATRDR	Predicted p	28.449	259		8	8	27
B7G0C3	PHATRDR	Tubulin alp	49.747	451		3	3	10.2
B7G2T9	PHATRDR	Carboxy-ly	88.336	818		9	9	13.6
B7G574	PHATRDR	Predicted p	28.842	253		3	3	12.6
B5Y3W7	PHATR_2	Tubulin be	49.582	447		3	3	8.7
B7FWS8	PHATRDR	Predicted p	20.054	180		3	3	19.4
B7G0V7	PHATRDR	Predicted p	50.457	455		1	1	2.6
B7G532	Atp1	ATP synth	54.505	509	B7G531	3	2	8.3
B7G871	Lhcf13	Protein fuc	21.53	197		2	2	11.2
B7GA85	PHATRDR	Predicted p	37.714	358		5	5	17.9
B7GCT8	PHATRDR	Ferredoxin	37.939	340	B7G0N7;B	3	3	11.2
B7GDL0	GPI_2	Glucose-6-	66.612	601		2	2	3.7
B5Y565	PHATR_4	Predicted p	48.581	446		5	5	13.5
B5Y5L4	Lhcf14	Fucoxanth	20.973	195		1	1	5.6
B7FV66	PHATRDR	Predicted p	33.957	324		1	1	3.1
B7FX68	H4-1b	Histone H4	11.383	103		3	3	22.3
B7FXQ8	HSP20A	Heat shock	18.4	163		1	1	9.8
B7FZB0	GltD	Synthase c	64.707	580		2	2	4.1
B7G134	PHATRDR	Glutamate-	49.542	468		3	3	5.8
B7G2S9	NDK2	Nucleoside	16.448	148	B7FPE8	5	5	31.1
B7G6K6	GapC2a	Glyceralde	35.418	334		4	4	19.5
B7GA81	PHATRDR	Predicted p	39.227	343		4	4	14
B7GCV4	PHATRDR	Predicted p	41.766	378		3	3	9.3
B7GEB5	ACC2	Acetyl-CoA	249.836	2282		3	3	1.5
B7GES3	PHATRDR	Predicted p	80.549	709		3	3	6.3
A0T0E2	rbcS	Multifunctio	16.023	139		2	2	20.1
B7FPI3	PHATRDR	Predicted p	102.547	976		4	4	5.2
B7FPR0	PHATRDR	Predicted p	47.602	430		4	4	10
B7FPX7	PHATRDR	tRNA-guar	42.52	390		2	2	6.9
B7FQE1	Lhcr4	Protein fuc	23.239	215		2	2	10.7
B7FRW2	Lhcf4	Protein fuc	21.328	198	B7FRW5;B	4	3	22.2
B7FS83	CK2_1	Predicted p	40.047	341		3	3	14.1
B7FVQ8	PHATRDR	Methylcrot	64.725	597		2	2	5
B7FVT2	PHATRDR	Predicted p	31.903	286		3	3	12.9
B7FXS8	PHATRDR	Predicted p	37.645	350		8	4	23.7

B7FYL2	ISIP2A	Iron starva	57.049	537		5	5	11.4
B7G0I5	PHATRDR	GMP synth	59.613	538		5	5	14.7
B7G0Q0	PHATRDR	Predicted p	36.328	329		1	1	6.7
B7G3I6	PHATRDR	Fucoxanth	25.439	235		1	1	6
B7G5S7	Lhcf6	Protein fuc	21.891	204		1	1	7.8
B7GAC5	PHATRDR	Predicted p	42.751	377		2	2	6.9
A0T0K6	tufA	Elongation	44.528	409		2	2	4.6
B5Y4M6	PHATR_43	Predicted p	53.391	497		2	2	6.2
B5Y4U7	PHATR_54	Glucosidas	88.95	802		4	4	5
B7FPZ0	PHATRDR	Predicted p	146.766	1343		2	2	2.9
B7FTT1	PHATRDR	Trafficking	16.971	152		2	2	11.8
B7FUA4	PHATRDR	Predicted p	55.136	530		3	3	5.8
B7FUY5	PHATRDR	Predicted p	34.855	323		3	3	13.3
B7G1L9	PHATRDR	Predicted p	22.145	203		4	4	17.2
B7G502	Lhcr13	Protein fuc	22.267	206		1	1	7.8
B7G6Q6	GLNA	GLNA, glut	79.636	716		2	2	4.2
B7G9K6	PHATRDR	Predicted p	53.421	513		2	2	4.7
B7S466	PHATRDR	S-adenosy	42.218	386		1	1	3.9
B7S4A6	PHATRDR	Predicted p	40.767	357		2	2	9
A0T0D2	atpB	ATP synth	51.621	475		4	2	15.2
B7FQE2	PHATRDR	2-oxoisova	38.562	341		1	1	4.1
B7FSP4	Lhcr3	Protein fuc	21.107	199		1	1	4.5
B7FSV5	PHATRDR	Protein xyl	92.356	811		3	3	3.9
B7FXP8	PHATRDR	Carbonic a	30.08	273		5	2	27.8
B7G195	PHATRDR	Predicted p	31.711	281		2	2	12.1
B7G4B9	PHATRDR	Predicted p	29.494	263		1	1	7.6
B7G4N2	PHATRDR	Predicted p	22.735	199		2	2	15.6
B7G503	Lhcr14	Protein fuc	21.335	198		2	2	13.1
B7G585	PPdK	Pyruvate, p	110.112	1011		2	2	2.7
B7G5R3	PHATRDR	Transketol	74.097	684		3	3	5
B7G6X2	PHATRDR	Predicted p	57.371	523		2	2	6.1
B7G8S0	PHATRDR	Predicted p	60.193	542		3	3	9.4
B7G955	Lhcf9	Protein fuc	22.101	205		1	1	5.9
B7GAW0	PHATRDR	Cystathion	42.384	392		2	2	7.7
B7GB55	PHATRDR	Predicted p	30.574	271		3	3	12.5
A0T0M1	psaF	Photosyste	20.557	185		1	1	11.9
B5Y594	SHMT	Serine hyd	52.188	473		1	1	1.5
B5Y5D2	ANXA1	Annexin O	37.464	333		2	2	7.2
B7FSI4	GapC4	Glyceralde	35.791	336		1	1	4.2
B7FTU0	PHATRDR	Predicted p	38.408	363		2	2	6.6
B7FUA8	PHATRDR	Predicted p	52.587	481		2	2	6
B7FUG2	PHATRDR	Malonyl-co	36.845	326		1	1	4.3
B7FY03	PHATRDR	Ribosome	25.094	220		1	1	3.6
B7FY59	PHATRDR	Predicted p	24.728	221		1	1	5.9
B7G0Q5	PHATRDR	Predicted p	37.122	338		1	1	2.4
B7G237	PHATRDR	Proly 4-hyd	25.842	226		1	1	5.8
B7G374	AP4beta	Predicted p	88.032	805		2	2	2.2
B7G3C4	PHATRDR	Elongation	48.056	439	B5Y4J2	1	1	2.5
B7G3W4	PHATRDR	Predicted p	62.723	580		1	1	1.4
B7G5M1	PHATRDR	Predicted p	34.97	317		1	1	4.7
B7G7A1	PHATRDR	Predicted p	54.951	495		1	1	3.6

B7G8E5	PHATRDR	Fucoxanth	21.63	199	B5Y583	3	3	18.6
B7G9P5	PHATRDR	Citrate syn	52.293	471		1	1	2.5
B7G9T6	PHATRDR	Predicted p	23.787	227		1	1	4.8
B7GA01	PHATRDR	Predicted p	61.252	566		2	2	3.2
B7GA49	PHATRDR	Predicted p	40.816	384		1	1	2.3
B7GAS4	Lhcr11	Protein fuc	26.171	246		1	1	5.3
B7GB07	PHATRDR	Predicted p	26.915	241		1	1	5
B7GEM5	PHATRDR	Predicted p	113.381	1008		3	3	4.6
B5Y4I2	PHATR_1(	ATP-deper	21.496	199		1	1	7
B5Y5N6	SDH1	Succinate	69.791	638		1	1	2.2
B7FNX9	PHATRDR	Predicted p	170.321	1528		1	1	0.7
B7FPH5	PF2K1	Bifunctiona	61.084	540		1	1	2
B7FPX0	PHATRDR	Predicted p	30.199	269		1	1	3.3
B7FQ82	PHATRDR	Predicted p	77.82	706		1	1	2.1
B7FRW4	Lhcf2	Protein fuc	21.296	198		1	0	4
B7FV40	PHATRDR	Predicted p	15.303	135		1	1	8.9
B7FVQ1	PHATRDR	Predicted p	35.521	325		2	2	6.8
B7FWB5	PHATRDR	Predicted p	47.607	429		1	1	3.7
B7FWI3	PHATRDR	Predicted p	208.024	1843		2	2	1.1
B7FWT1	PHATRDR	Predicted p	54.752	513		1	1	1.4
B7FXX2	Sar1a	Predicted p	21.824	192		1	1	5.7
B7FZF0	PHATRDR	Predicted p	45.377	405		1	1	3.5
B7FZP1	PHATRDR	Proteasom	21.343	201		1	1	5
B7G128	PHATRDR	Predicted p	22.502	221		1	1	7.7
B7G1G0	PHATRDR	Predicted p	80.345	723		1	1	1.7
B7G1T2	PHATRDR	Predicted p	23.594	211		1	1	5.7
B7G3D4	FABD	[Acyl-carrie	37.437	352		1	1	3.1
B7G3J8	PHATRDR	Predicted p	21.784	199		1	1	4.5
B7G3M4	PHATRDR	Predicted p	28.013	247		1	1	2.4
B7G3Q4	PHATRDR	Predicted p	51.538	467		1	1	2.8
B7G5Q1	GapC1	Glyceralde	40.183	379		1	1	1.8
B7G5Y2	PHATRDR	14-3-3-like	27.066	241		1	1	4.1
B7G660	PHATRDR	Predicted p	13.69	127		1	1	8.7
B7G6H0	PHATRDR	Phosphogl	45.98	436		1	1	2.5
B7G6N9	PHATRDR	Predicted p	60.514	549		1	1	2
B7G7G5	PHATRDR	Predicted p	54.82	486		1	1	1.6
B7G7Q7	PHATRDR	Predicted p	36.884	325		1	1	2.8
B7G7W4	PHATRDR	Predicted p	24.083	226		1	1	6.2
B7G833	PHATRDR	Predicted p	63.702	571		1	1	2.1
B7G857	PHATRDR	Predicted p	59.204	540		1	1	2.2
B7G8F5	PHATRDR	Predicted p	61.964	561		1	1	2.7
B7G9U1	PHATRDR	Predicted p	136.61	1263		1	1	0.7
B7GAX2	PHATRDR	Predicted p	59.933	541		1	1	1.8
B7GBB9	PK6	Pyruvate k	59.775	556		1	1	1.6
B7GCR5	PHATRDR	Predicted p	50.351	456		1	1	2.4
B7S3N8	FbaC1	Fructose-b	43.58	402		1	1	2.5
B7S422	MPDC	Diphospho	45.215	415		1	1	3.1
B7FQ36	PHATRDR	Predicted p	165.432	1474		1	1	0.5
B7FRD6	PSAT	Phosphose	44.934	409		1	1	2.7
B7FTA0	hBRM	Predicted p	113.793	995		1	1	1
B7FVB2	PHATRDR	Predicted p	66.016	592		1	1	1.9

B7FWA9	PHATRDR	Predicted p	48.344	422	1	1	3.3
B7FYY2	PHATRDR	Predicted p	80.042	697	1	1	1.1
B7G218	H3.3	Histone H3	15.327	136	1	1	5.9
B7G6X5	PHATRDR	Predicted p	47.814	432	1	1	3
B7G9W4	PHATRDR	Predicted p	83.562	726	1	1	1.5
B7GBE2	PHATRDR	Predicted p	23.912	218	1	1	6.4

Intensity E	iBAQ Bn3	iBAQ Bn3	LFQ inten	MS/MS co	SAF Bn3	Protein group ID
1.93E+11	8.79E+09	94.14481	6.9E+10	463	1.112981	318
17112000	658140	0.007046	5334200	1	0.002041	54
28214000	829830	0.008884	22708000	3	0.004016	310
4.39E+08	10451000	0.111888	2.04E+08	19	0.02043	269
2.65E+08	26548000	0.284221	1.29E+08	7	0.054688	166
39485000	1128100	0.012077	32594000	3	0.005455	176
1.58E+09	65708000	0.703466	6.41E+08	28	0.057613	126
2.88E+08	6130800	0.065636	1.16E+08	22	0.021891	242
3.14E+08	34907000	0.373712	89624000	7	0.047297	150
1.65E+08	5502700	0.058912	76263000	12	0.026786	187
2.29E+08	11459000	0.122679	1.24E+08	12	0.034783	282
4.64E+08	20166000	0.215896	2.1E+08	15	0.041096	214
2.28E+08	11988000	0.128343	72481000	14	0.040462	155
1891700	105090	0.001125	1325800	0	0	323
976790	11769	0.000126	611450	0	0	292
2.08E+08	3715300	0.039776	88138000	13	0.010842	266
18297000	315470	0.003377	13989000	3	0.002796	228
1.91E+08	12763000	0.13664	81533000	7	0.021148	180
688480	8714.9	9.33E-05	463100	0	0	331
4.78E+08	29868000	0.319765	1.84E+08	12	0.042553	77
3.25E+08	20312000	0.217459	1.54E+08	11	0.037037	159
4.14E+08	8453700	0.090505	87775000	8	0.009112	263
4341200	124030	0.001328	2890500	1	0.00102	106
3.8E+08	10558000	0.113033	1.46E+08	16	0.022472	149
2.17E+08	10865000	0.11632	87575000	10	0.028571	163
34452000	1148400	0.012295	18099000	3	0.006803	235
10552000	82434	0.000883	5614800	0	0	260
9.37E+08	26030000	0.278676	3.99E+08	13	0.017497	296
2.35E+08	5465900	0.058518	97737000	19	0.025815	66
12197000	451740	0.004836	11002000	2	0.004283	68
16566000	1035300	0.011084	11974000	0	0	105
1.89E+08	4841600	0.051834	56428000	13	0.019727	131
1.2E+08	10946000	0.117187	68420000	6	0.038961	289
1.61E+08	12422000	0.132989	62771000	8	0.032258	71
1.02E+08	5386800	0.057671	35508000	10	0.030303	118
55637000	1854600	0.019855	16705000	6	0.009885	335
22278000	856860	0.009173	13294000	2	0.003802	53
1.08E+08	4311900	0.046163	41818000	9	0.017045	219
25636000	801120	0.008577	12374000	2	0.00361	279
1.01E+08	2899300	0.03104	43184000	9	0.01312	308
32070000	1105900	0.01184	12598000	4	0.010336	125
1.7E+08	7095100	0.07596	91152000	5	0.013263	234
26184000	969770	0.010382	10362000	0	0	95
31108000	1829900	0.019591	14643000	2	0.006431	208
1.06E+08	5040300	0.053961	40264000	6	0.016086	301
11573000	361640	0.003872	6872300	1	0.001361	317
16474000	658970	0.007055	8292300	3	0.005941	50
43646000	909280	0.009735	18108000	7	0.009845	137
782540	23713	0.000254	478150	0	0	147
10945000	254530	0.002725	6731200	4	0.005814	240

3629800	125170	0.00134	2115900	0	0	244
65351000	1815300	0.019434	23446000	8	0.012618	311
32260000	1075300	0.011512	12876000	4	0.007984	110
23954000	1497100	0.016028	13769000	2	0.006309	182
48902000	1528200	0.016361	19668000	8	0.01054	79
11467000	301770	0.003231	3241200	2	0.003063	98
3244400	190850	0.002043	2469900	0	0	136
3636300	134680	0.001442	2660700	1	0.001431	142
2191900	121770	0.001304	1786200	0	0	169
51785000	1204300	0.012893	19064000	6	0.008197	223
5752700	239700	0.002566	2283200	0	0	246
11773000	436040	0.004668	6664300	0	0	261
26638000	859290	0.0092	10127000	6	0.012903	303
5611700	79038	0.000846	2926200	1	0.000584	61
6172100	293910	0.003147	3671400	1	0.002941	132
8074100	310540	0.003325	4390500	2	0.004228	135
1.08E+08	6742400	0.072184	48026000	7	0.027027	183
18178000	790360	0.008462	7844000	3	0.006652	184
54291000	1357300	0.014531	23190000	8	0.00978	210
17228000	1325200	0.014188	5962600	2	0.007905	232
7058500	320840	0.003435	4553300	2	0.004474	58
16747000	1395500	0.01494	7013600	3	0.016667	156
1292600	80791	0.000865	1407100	0	0	191
4164400	138810	0.001486	2014300	2	0.003929	231
31688000	4526800	0.048464	12668000	3	0.015228	268
52359000	3739900	0.040039	21532000	7	0.019553	294
20631000	896990	0.009603	8030400	2	0.005882	320
8830200	284850	0.00305	7122700	0	0	325
19204000	914490	0.00979	7761600	4	0.008969	67
2702700	270270	0.002893	2973000	1	0.005128	75
5722100	317900	0.003403	2478900	1	0.003086	140
20169000	3361400	0.035987	8445100	3	0.029126	158
655970	65597	0.000702	581010	0	0	161
15523000	456560	0.004888	8097500	2	0.003448	177
6924000	301040	0.003223	3365100	1	0.002137	195
58619000	7327400	0.078447	23773000	6	0.040541	209
8830700	588710	0.006303	3523500	0	0	247
1.29E+08	7189700	0.076972	54514000	4	0.011662	293
35895000	1889200	0.020226	17380000	2	0.005291	322
11447000	87384	0.000936	5641800	2	0.000876	329
8190500	204760	0.002192	2630800	1	0.00141	333
0	NA	NA	0	0	0	55
20561000	761510	0.008153	8907200	3	0.003074	87
17609000	800390	0.008569	7628300	5	0.011628	89
3513400	167300	0.001791	2054800	0	0	92
7521800	683800	0.007321	3407000	1	0.004651	100
40280000	4475500	0.047914	17571000	2	0.010101	108
10410000	612330	0.006556	3159600	1	0.002933	111
2914000	91062	0.000975	3166000	1	0.001675	146
37759000	2904500	0.031095	15575000	3	0.01049	148
31281000	1360000	0.01456	16660000	4	0.011429	162

30678000	1136200	0.012164	9822300	5	0.009311	171
28524000	864360	0.009254	12508000	5	0.009294	188
1174800	58740	0.000629	475420	0	0	189
1741400	193490	0.002071	1510700	1	0.004255	216
1495200	213600	0.002287	3237000	0	0	239
12340000	474610	0.005081	5327300	2	0.005305	295
11368000	541320	0.005795	5134300	1	0.002445	51
6135100	266740	0.002856	2525800	1	0.002012	64
22746000	614770	0.006582	9644600	4	0.004988	65
8079600	115420	0.001236	3551900	1	0.000745	93
5296100	529610	0.00567	2371600	1	0.006579	123
36871000	3687100	0.039474	16139000	3	0.00566	129
9464100	591510	0.006333	3712000	2	0.006192	138
13041000	1086700	0.011634	5359600	4	0.019704	201
1231700	136850	0.001465	1475200	0	0	229
3382300	89008	0.000953	1014400	1	0.001397	250
2865400	136450	0.001461	930260	0	0	281
5772200	274870	0.002943	3548200	1	0.002591	337
829720	41486	0.000444	2953200	1	0.002801	338
3872200	161340	0.001727	1187800	1	0.002105	49
902990	53117	0.000569	1837400	0	0	101
6528200	725360	0.007766	6231600	1	0.005025	114
10041000	251040	0.002688	4244300	3	0.003699	115
39134000	3010300	0.032228	16779000	3	0.010989	160
5200300	346680	0.003712	2331400	1	0.003559	197
4051400	311650	0.003337	1755100	1	0.003802	225
8460100	1208600	0.012939	3350500	1	0.005025	227
4431700	443170	0.004745	1294000	0	0	230
7458300	122270	0.001309	2619900	2	0.001978	233
33234000	1007100	0.010782	14436000	3	0.004386	238
7666400	283940	0.00304	3215900	2	0.003824	252
2920400	97346	0.001042	1265200	3	0.005535	275
518660	57629	0.000617	559500	0	0	278
18965000	1115600	0.011944	6516200	2	0.005102	298
51164000	4263700	0.045647	25668000	1	0.00369	302
1617800	161780	0.001732	700850	0	0	56
1702700	65487	0.000701	1080700	0	0	70
8620500	374800	0.004013	3735700	2	0.006006	72
1773800	80626	0.000863	0	1	0.002976	112
3782800	210150	0.00225	1110200	2	0.00551	124
4839300	254700	0.002727	2261300	2	0.004158	130
981600	44618	0.000478	728860	0	0	133
51993000	3713800	0.03976	22524000	0	0	167
3071200	383900	0.00411	1330500	1	0.004525	168
1078000	76997	0.000824	1540700	0	0	190
7303200	561790	0.006014	4822900	1	0.004425	207
4194300	97542	0.001044	1885600	2	0.002484	211
1942200	80924	0.000866	841380	0	0	63
2022700	69749	0.000747	1024500	1	0.001724	222
2657800	120810	0.001293	2567700	0	0	236
2186800	84106	0.0009	947340	1	0.00202	256

6339300	576300	0.00617	1801500	0	0	270
8469800	352910	0.003778	1930100	1	0.002123	283
2172400	434480	0.004652	941120	0	0	284
8665800	279540	0.002993	1984100	0	0	287
1620500	162050	0.001735	1451400	0	0	291
1125200	112520	0.001205	2206900	0	0	297
1707400	131340	0.001406	1823000	1	0.004149	300
9879800	197600	0.002115	4100800	2	0.001984	332
910000	101110	0.001082	394230	0	0	62
5794300	152480	0.001632	2510200	1	0.001567	76
5609000	64471	0.00069	2260700	0	0	78
1100000	32353	0.000346	476530	0	0	86
1171900	61677	0.00066	299690	0	0	91
5337500	144260	0.001544	2711500	0	0	97
0	NA	NA	0	0	0	109
1762700	146890	0.001573	809090	0	0	139
25142000	1571400	0.016823	10842000	1	0.003077	145
2319400	96642	0.001035	665770	0	0	152
33224000	255570	0.002736	14393000	1	0.000543	154
10085000	373530	0.003999	4369100	1	0.001949	157
3795400	291950	0.003126	1788800	0	0	164
3925700	178440	0.00191	1700700	0	0	178
1606700	160670	0.00172	696060	0	0	181
1220200	135580	0.001452	1693400	0	0	194
1797100	40843	0.000437	778530	1	0.001383	200
1100700	84670	0.000906	476850	0	0	205
1780800	104750	0.001121	771480	0	0	213
10856000	1206200	0.012914	5022500	1	0.005025	217
8148700	370400	0.003965	3530100	1	0.004049	220
1.33E+08	4736100	0.050704	59219000	1	0.002141	221
760240	40013	0.000428	217690	0	0	237
1514200	100950	0.001081	475160	0	0	241
854980	122140	0.001308	503130	0	0	243
347300	12403	0.000133	184870	0	0	245
899690	32132	0.000344	301530	0	0	249
1.25E+09	43235000	0.462871	5.43E+08	1	0.002058	258
952410	56024	0.0006	412600	0	0	259
1758400	135260	0.001448	761750	0	0	262
1974000	61689	0.00066	855190	1	0.001751	265
76331000	3053200	0.032687	33068000	1	0.001852	267
3908700	139600	0.001495	1693300	1	0.001783	271
0	NA	NA	0	1	0.000792	285
2008400	87321	0.000935	578210	0	0	299
2273300	61441	0.000658	1073700	1	0.001799	304
1068300	35611	0.000381	462820	0	0	319
1391600	57985	0.000621	602880	1	0.002488	334
2143500	89312	0.000956	928590	1	0.00241	336
0	NA	NA	0	0	0	94
0	NA	NA	0	0	0	107
0	NA	NA	0	0	0	119
0	NA	NA	0	0	0	141

0	NA	NA	0	0	0	151
0	NA	NA	0	0	0	175
823710	137290	0.00147	356850	0	0	206
0	NA	NA	0	0	0	253
0	NA	NA	0	0	0	286
0	NA	NA	0	0	0	306
