# Supplementary material for: Cryptochrome PtCPF1 regulates high temperature acclimation of marine diatoms through coordination of iron and phosphorus uptake
Source: ISME J. 2024 Jan 10;18(1):wrad019. doi: 10.1093/ismejo/wrad019 (PMC10837835; doi:10.1093/ismejo/wrad019)
Supplement: 20231201_Supplementary_tables_S7_wrad019 [file 20231201_supplementary_tables_s7_wrad019.pdf]

**Table S7**. The three bands were excised from the BN gel with CBB staining (Figure 7) and analyzed by LC-ESI-MS/MS analysis.The LC--ESI-MS/MS results included a lot of proteins based on the *P. tricornutum* database downloaded from Uniprot. See Supplementary Table S4 for details of the LC-ESI-MS/MS analysis. The cut-off values are both peptide number and unique peptide number >1. NCBI were also used to annotate the gene name based on gene ID. See Supplementary Table S4 for details of the LC-ESI-MS/MS analysis.

| Name | ID | Peptides BN1 | Unique peptides BN1 | Peptides BN2 | Unique peptides BN2 | Peptides BN3 | Unique peptides BN3 |
|---|---|---|---|---|---|---|---|
| PtCPF1 | 27429 | 26 | 26 | 22 | 22 | 14 | 14 |
| UBI 1/2 | 22043 | 11 | 11 | 8 | 8 | 9 | 9 |
| Eps15 | 42442 | 13 | 13 | 8 | 8 | 3 | 3 |
| Nucleotide-Binding Domain | bd1628 | 4 | 4 | 8 | 8 | 8 | 8 |
| Hsp70_2 | 55890 | 7 | 5 | 5 | 3 | 9 | 6 |
| Transcription factor IIA | 42776 | 2 | 2 | 1 | 1 | 4 | 4 |
| HSP70A | 54019 | 6 | 6 | 1 | 1 | 4 | 4 |
| BolA | 14849 | 1 | 1 | 1 | 1 | 1 | 1 |
