# Supplementary material for: Cryptochrome PtCPF1 regulates high temperature acclimation of marine diatoms through coordination of iron and phosphorus uptake
Source: ISME J. 2024 Jan 10;18(1):wrad019. doi: 10.1093/ismejo/wrad019 (PMC10837835; doi:10.1093/ismejo/wrad019)
Supplement: 20231201_Supplementary_tables_S8_wrad019 [file 20231201_supplementary_tables_s8_wrad019.pdf]

Table S8 Bioinformatic predication analysis of the target genes of BolA and TF IIA

#pattern	TF	target	start	stop	strand	score	p-value	q-value
MA1020.1	Phatr3_J14849	Phatr3_EG	1534	1541	-	14.0632	1.79E-05	1
MA1020.1	Phatr3_J14849	Phatr3_J4	173	180	-	14.0632	1.79E-05	1
MA1020.1	Phatr3_J14849	Phatr3_J3	1433	1440	+	14.0632	1.79E-05	1
MA1020.1	Phatr3_J14849	Phatr3_J3	1431	1438	-	14.0632	1.79E-05	1
MA1020.1	Phatr3_J14849	Phatr3_J4	408	415	-	14.0632	1.79E-05	1
MA1020.1	Phatr3_J14849	Phatr3_EG	170	177	+	14.0632	1.79E-05	1
MA1020.1	Phatr3_J14849	Phatr3_EG	1111	1118	-	14.0632	1.79E-05	1
MA1020.1	Phatr3_J14849	Phatr3_J5	376	383	+	14.0632	1.79E-05	1
MA1020.1	Phatr3_J14849	Phatr3_J1	1921	1928	+	14.0632	1.79E-05	1
MA1020.1	Phatr3_J14849	Phatr3_J4	872	879	-	14.0632	1.79E-05	1
MA1020.1	Phatr3_J14849	Phatr3_J3	626	633	+	14.0632	1.79E-05	1
MA1020.1	Phatr3_J14849	Phatr3_J3	9	16	-	14.0632	1.79E-05	1
MA1020.1	Phatr3_J14849	Phatr3_EG	919	926	-	14.0632	1.79E-05	1
MA1020.1	Phatr3_J14849	Phatr3_J1	810	817	-	14.0632	1.79E-05	1
MA1020.1	Phatr3_J14849	Phatr3_J2	1717	1724	+	14.0632	1.79E-05	1
MA1020.1	Phatr3_J14849	Phatr3_J5	586	593	-	14.0632	1.79E-05	1
MA1020.1	Phatr3_J14849	Phatr3_J4	1274	1281	-	14.0632	1.79E-05	1
MA1020.1	Phatr3_J14849	Phatr3_J4	1325	1332	-	14.0632	1.79E-05	1
MA1020.1	Phatr3_J14849	Phatr3_EG	886	893	-	14.0632	1.79E-05	1
MA1020.1	Phatr3_J14849	Phatr3_J4	333	340	+	14.0632	1.79E-05	1
MA1020.1	Phatr3_J14849	Phatr3_J4	1076	1083	-	14.0632	1.79E-05	1
MA1020.1	Phatr3_J14849	Phatr3_J3	1853	1860	+	14.0632	1.79E-05	1
MA1020.1	Phatr3_J14849	Phatr3_J2	781	788	+	14.0632	1.79E-05	1
MA1020.1	Phatr3_J14849	Phatr3_J4	712	719	-	14.0632	1.79E-05	1
MA1020.1	Phatr3_J14849	Phatr3_J3	1620	1627	-	14.0632	1.79E-05	1
MA1020.1	Phatr3_J14849	Phatr3_J2	1037	1044	-	14.0632	1.79E-05	1
MA1020.1	Phatr3_J14849	Phatr3_J2	1851	1858	+	14.0632	1.79E-05	1
MA1020.1	Phatr3_J14849	Phatr3_J2	1092	1099	-	14.0632	1.79E-05	1
MA1020.1	Phatr3_J14849	Phatr3_J4	1476	1483	+	14.0632	1.79E-05	1
MA1020.1	Phatr3_J14849	Phatr3_J4	569	576	-	14.0632	1.79E-05	1
MA1020.1	Phatr3_J14849	Phatr3_J3	1471	1478	+	14.0632	1.79E-05	1
MA1020.1	Phatr3_J14849	Phatr3_J2	1635	1642	-	14.0632	1.79E-05	1
MA1020.1	Phatr3_J14849	Phatr3_J4	1353	1360	+	14.0632	1.79E-05	1
MA1020.1	Phatr3_J14849	Phatr3_EG	1457	1464	+	14.0632	1.79E-05	1
MA1020.1	Phatr3_J14849	Phatr3_J4	1124	1131	-	14.0632	1.79E-05	1
MA1020.1	Phatr3_J14849	Phatr3_J4	927	934	-	14.0632	1.79E-05	1
MA1020.1	Phatr3_J14849	Phatr3_EG	1910	1917	+	14.0632	1.79E-05	1
MA1020.1	Phatr3_J14849	Phatr3_EG	162	169	+	14.0632	1.79E-05	1
MA1020.1	Phatr3_J14849	Phatr3_J4	427	434	+	14.0632	1.79E-05	1
MA1020.1	Phatr3_J14849	Phatr3_J2	581	588	-	14.0632	1.79E-05	1
MA1020.1	Phatr3_J14849	Phatr3_EG	279	286	+	14.0632	1.79E-05	1
MA1020.1	Phatr3_J14849	Phatr3_EG	1648	1655	+	14.0632	1.79E-05	1
MA1020.1	Phatr3_J14849	Phatr3_EG	1646	1653	-	14.0632	1.79E-05	1
MA1020.1	Phatr3_J14849	Phatr3_J1	1213	1220	+	14.0632	1.79E-05	1
MA1020.1	Phatr3_J14849	Phatr3_J1	1211	1218	-	14.0632	1.79E-05	1
MA1020.1	Phatr3_J14849	Phatr3_J1	511	518	+	14.0632	1.79E-05	1
MA1020.1	Phatr3_J14849	Phatr3_J4	318	325	+	14.0632	1.79E-05	1
MA1020.1	Phatr3_J14849	Phatr3_J5	4	11	+	14.0632	1.79E-05	1
MA1020.1	Phatr3_J14849	Phatr3_J3	1547	1554	-	14.0632	1.79E-05	1
MA1020.1	Phatr3_J14849	Phatr3_J4	37	44	-	14.0632	1.79E-05	1
MA1020.1	Phatr3_J14849	Phatr3_J1	904	911	-	14.0632	1.79E-05	1
MA1020.1	Phatr3_J14849	Phatr3_J4	494	501	-	14.0632	1.79E-05	1
MA1020.1	Phatr3_J14849	Phatr3_J4	688	695	-	14.0632	1.79E-05	1

MA1020.1	Phatr3_J14849	Phatr3_J4	1346	1353	+	14.0632	1.79E-05	1
MA1020.1	Phatr3_J14849	Phatr3_J1	219	226	-	14.0632	1.79E-05	1
MA1020.1	Phatr3_J14849	Phatr3_J1	376	383	+	14.0632	1.79E-05	1
MA1020.1	Phatr3_J14849	Phatr3_J4	1917	1924	+	14.0632	1.79E-05	1
MA1020.1	Phatr3_J14849	Phatr3_E(	812	819	+	14.0632	1.79E-05	1
MA1020.1	Phatr3_J14849	Phatr3_J4	1056	1063	-	14.0632	1.79E-05	1
MA1020.1	Phatr3_J14849	Phatr3_J4	1453	1460	+	14.0632	1.79E-05	1
MA1020.1	Phatr3_J14849	Phatr3_J4	1451	1458	-	14.0632	1.79E-05	1
MA1020.1	Phatr3_J14849	Phatr3_J2	184	191	-	14.0632	1.79E-05	1
MA1020.1	Phatr3_J14849	Phatr3_E(	1837	1844	-	12.5202	3.26E-05	1
MA1020.1	Phatr3_J14849	Phatr3_J4	868	875	+	12.5202	3.26E-05	1
MA1020.1	Phatr3_J14849	Phatr3_J4	1675	1682	-	12.5202	3.26E-05	1
MA1020.1	Phatr3_J14849	Phatr3_J4	813	820	+	12.5202	3.26E-05	1
MA1020.1	Phatr3_J14849	Phatr3_E(	1871	1878	+	12.5202	3.26E-05	1
MA1020.1	Phatr3_J14849	Phatr3_J1	183	190	-	12.5202	3.26E-05	1
MA1020.1	Phatr3_J14849	Phatr3_J4	1532	1539	-	12.5202	3.26E-05	1
MA1020.1	Phatr3_J14849	Phatr3_J1	377	384	+	12.5202	3.26E-05	1
MA1020.1	Phatr3_J14849	Phatr3_J3	1415	1422	+	12.5202	3.26E-05	1
MA1020.1	Phatr3_J14849	Phatr3_J4	1871	1878	+	12.5202	3.26E-05	1
MA1020.1	Phatr3_J14849	Phatr3_J3	1723	1730	+	12.5202	3.26E-05	1
MA1020.1	Phatr3_J14849	Phatr3_E(	1747	1754	+	12.5202	3.26E-05	1
MA1020.1	Phatr3_J14849	Phatr3_J3	280	287	+	12.5202	3.26E-05	1
MA1020.1	Phatr3_J14849	Phatr3_J1	3	10	-	12.5202	3.26E-05	1
MA1020.1	Phatr3_J14849	Phatr3_J1	995	1002	+	12.5202	3.26E-05	1
MA1020.1	Phatr3_J14849	Phatr3_J3	1592	1599	+	12.5202	3.26E-05	1
MA1020.1	Phatr3_J14849	Phatr3_E(	541	548	-	12.5202	3.26E-05	1
MA1020.1	Phatr3_J14849	Phatr3_J4	1735	1742	-	12.5202	3.26E-05	1
MA1020.1	Phatr3_J14849	Phatr3_J4	467	474	+	12.5202	3.26E-05	1
MA1020.1	Phatr3_J14849	Phatr3_J4	1454	1461	+	12.5202	3.26E-05	1
MA1020.1	Phatr3_J14849	Phatr3_J4	1721	1728	+	12.5202	3.26E-05	1
MA1020.1	Phatr3_J14849	Phatr3_J4	953	960	-	12.5202	3.26E-05	1
MA1020.1	Phatr3_J14849	Phatr3_J4	425	432	-	12.5202	3.26E-05	1
MA1020.1	Phatr3_J14849	Phatr3_J1	436	443	+	12.5202	3.26E-05	1
MA1020.1	Phatr3_J14849	Phatr3_J3	1765	1772	-	12.5202	3.26E-05	1
MA1020.1	Phatr3_J14849	Phatr3_J4	39	46	+	12.5202	3.26E-05	1
MA1020.1	Phatr3_J14849	Phatr3_J5	1730	1737	+	12.5202	3.26E-05	1
MA1020.1	Phatr3_J14849	Phatr3_J4	1967	1974	+	12.5202	3.26E-05	1
MA1020.1	Phatr3_J14849	Phatr3_E(	1735	1742	-	12.5202	3.26E-05	1
MA1020.1	Phatr3_J14849	Phatr3_J4	349	356	+	12.5202	3.26E-05	1
MA1020.1	Phatr3_J14849	Phatr3_J4	496	503	+	12.5202	3.26E-05	1
MA1020.1	Phatr3_J14849	Phatr3_J4	576	583	+	12.5202	3.26E-05	1
MA1020.1	Phatr3_J14849	Phatr3_J4	690	697	+	12.5202	3.26E-05	1
MA1020.1	Phatr3_J14849	Phatr3_J4	1559	1566	+	12.5202	3.26E-05	1
MA1020.1	Phatr3_J14849	Phatr3_J1	1527	1534	+	12.5202	3.26E-05	1
MA1020.1	Phatr3_J14849	Phatr3_J1	909	916	-	12.5202	3.26E-05	1
MA1020.1	Phatr3_J14849	Phatr3_J3	1639	1646	+	12.5202	3.26E-05	1
MA1020.1	Phatr3_J14849	Phatr3_J4	1000	1007	-	12.5202	3.26E-05	1
MA1020.1	Phatr3_J14849	Phatr3_J4	403	410	+	12.5202	3.26E-05	1
MA1020.1	Phatr3_J14849	Phatr3_J5	689	696	+	12.5202	3.26E-05	1
MA1020.1	Phatr3_J14849	Phatr3_J5	1038	1045	+	12.5202	3.26E-05	1
MA1020.1	Phatr3_J14849	Phatr3_J4	1084	1091	-	12.5202	3.26E-05	1
MA1020.1	Phatr3_J14849	Phatr3_J4	1504	1511	-	12.5202	3.26E-05	1
MA1020.1	Phatr3_J14849	Phatr3_J3	675	682	+	12.0573	8.69E-05	1
MA1020.1	Phatr3_J14849	Phatr3_J4	1024	1031	+	12.0573	8.69E-05	1

MA1020.1	Phatr3_J14849	Phatr3_J5	1604	1611	+	12.0573	8.69E-05	1
MA1020.1	Phatr3_J14849	Phatr3_J6	524	531	-	12.0573	8.69E-05	1
MA1020.1	Phatr3_J14849	Phatr3_J3	1615	1622	-	12.0573	8.69E-05	1
MA1020.1	Phatr3_J14849	Phatr3_EG	1263	1270	-	12.0573	8.69E-05	1
MA1020.1	Phatr3_J14849	Phatr3_J1	704	711	+	12.0573	8.69E-05	1
MA1020.1	Phatr3_J14849	Phatr3_J1	913	920	+	12.0573	8.69E-05	1
MA1020.1	Phatr3_J14849	Phatr3_EG	817	824	-	12.0573	8.69E-05	1
MA1020.1	Phatr3_J14849	Phatr3_J4	699	706	+	12.0573	8.69E-05	1
MA1020.1	Phatr3_J14849	Phatr3_J3	511	518	+	12.0573	8.69E-05	1
MA1020.1	Phatr3_J14849	Phatr3_J4	1023	1030	+	12.0573	8.69E-05	1
MA1020.1	Phatr3_J14849	Phatr3_EG	311	318	+	12.0573	8.69E-05	1
MA1020.1	Phatr3_J14849	Phatr3_EG	649	656	-	12.0573	8.69E-05	1
MA1020.1	Phatr3_J14849	Phatr3_J3	385	392	-	12.0573	8.69E-05	1
MA1020.1	Phatr3_J14849	Phatr3_J4	1082	1089	+	12.0573	8.69E-05	1
MA1020.1	Phatr3_J14849	Phatr3_J4	100	107	+	12.0573	8.69E-05	1
MA1020.1	Phatr3_J14849	Phatr3_J4	1497	1504	+	12.0573	8.69E-05	1
MA1020.1	Phatr3_J14849	Phatr3_EG	1714	1721	-	12.0573	8.69E-05	1
MA1020.1	Phatr3_J14849	Phatr3_J4	358	365	+	12.0573	8.69E-05	1
MA1020.1	Phatr3_J14849	Phatr3_J3	413	420	-	12.0573	8.69E-05	1
MA1020.1	Phatr3_J14849	Phatr3_J4	1753	1760	+	12.0573	8.69E-05	1
MA1020.1	Phatr3_J14849	Phatr3_EG	1747	1754	-	12.0573	8.69E-05	1
MA1020.1	Phatr3_J14849	Phatr3_EG	594	601	+	12.0573	8.69E-05	1
MA1020.1	Phatr3_J14849	Phatr3_J3	1694	1701	-	12.0573	8.69E-05	1
MA1020.1	Phatr3_J14849	Phatr3_EG	871	878	+	12.0573	8.69E-05	1
MA1020.1	Phatr3_J14849	Phatr3_J2	528	535	-	12.0573	8.69E-05	1
MA1020.1	Phatr3_J14849	Phatr3_J3	91	98	-	12.0573	8.69E-05	1
MA1020.1	Phatr3_J14849	Phatr3_J4	1415	1422	-	12.0573	8.69E-05	1
MA1020.1	Phatr3_J14849	Phatr3_J1	1117	1124	+	12.0573	8.69E-05	1
MA1020.1	Phatr3_J14849	Phatr3_J3	1468	1475	+	12.0573	8.69E-05	1
MA1020.1	Phatr3_J14849	Phatr3_J4	1636	1643	+	12.0573	8.69E-05	1
MA1020.1	Phatr3_J14849	Phatr3_EG	312	319	-	12.0573	8.69E-05	1
MA1020.1	Phatr3_J14849	Phatr3_EG	560	567	+	12.0573	8.69E-05	1
MA1020.1	Phatr3_J14849	Phatr3_J5	529	536	-	12.0573	8.69E-05	1
MA1020.1	Phatr3_J14849	Phatr3_J4	832	839	+	12.0573	8.69E-05	1
MA1020.1	Phatr3_J14849	Phatr3_J4	103	110	-	12.0573	8.69E-05	1
MA1020.1	Phatr3_J14849	Phatr3_J3	1388	1395	+	12.0573	8.69E-05	1
MA1020.1	Phatr3_J14849	Phatr3_J3	1353	1360	+	12.0573	8.69E-05	1
MA1020.1	Phatr3_J14849	Phatr3_J3	1887	1894	+	12.0573	8.69E-05	1
MA1020.1	Phatr3_J14849	Phatr3_EG	75	82	-	12.0573	8.69E-05	1
MA1020.1	Phatr3_J14849	Phatr3_J4	598	605	-	12.0573	8.69E-05	1
MA1020.1	Phatr3_J14849	Phatr3_J1	995	1002	-	12.0573	8.69E-05	1
MA1020.1	Phatr3_J14849	Phatr3_J4	94	101	+	12.0573	8.69E-05	1
MA1020.1	Phatr3_J14849	Phatr3_J4	66	73	+	12.0573	8.69E-05	1
MA1020.1	Phatr3_J14849	Phatr3_J1	201	208	-	12.0573	8.69E-05	1
MA1020.1	Phatr3_J14849	Phatr3_EG	1247	1254	-	12.0573	8.69E-05	1
MA1020.1	Phatr3_J14849	Phatr3_J4	237	244	+	12.0573	8.69E-05	1
MA1020.1	Phatr3_J14849	Phatr3_EG	151	158	-	12.0573	8.69E-05	1
MA1020.1	Phatr3_J14849	Phatr3_J1	1695	1702	+	12.0573	8.69E-05	1
MA1020.1	Phatr3_J14849	Phatr3_J4	792	799	-	12.0573	8.69E-05	1
MA1020.1	Phatr3_J14849	Phatr3_J3	127	134	+	12.0573	8.69E-05	1
MA1020.1	Phatr3_J14849	Phatr3_J4	1976	1983	+	12.0573	8.69E-05	1
MA1020.1	Phatr3_J14849	Phatr3_J4	1418	1425	+	12.0573	8.69E-05	1
MA1020.1	Phatr3_J14849	Phatr3_J4	1647	1654	+	12.0573	8.69E-05	1
MA1020.1	Phatr3_J14849	Phatr3_J1	1932	1939	+	12.0573	8.69E-05	1

MA1020.1	Phatr3_J14849	Phatr3_J4	351	358	+	12.0573	8.69E-05	1
MA1020.1	Phatr3_J14849	Phatr3_J4	1721	1728	-	12.0573	8.69E-05	1
MA1020.1	Phatr3_J14849	Phatr3_J1	1359	1366	-	12.0573	8.69E-05	1
MA1020.1	Phatr3_J14849	Phatr3_J4	438	445	+	12.0573	8.69E-05	1
MA1020.1	Phatr3_J14849	Phatr3_J4	1704	1711	-	12.0573	8.69E-05	1
MA1020.1	Phatr3_J14849	Phatr3_J5	95	102	+	12.0573	8.69E-05	1
MA1020.1	Phatr3_J14849	Phatr3_E0	1234	1241	+	12.0573	8.69E-05	1
MA1020.1	Phatr3_J14849	Phatr3_E0	441	448	+	12.0573	8.69E-05	1
MA1020.1	Phatr3_J14849	Phatr3_J4	177	184	-	12.0573	8.69E-05	1
MA1020.1	Phatr3_J14849	Phatr3_J4	1952	1959	+	12.0573	8.69E-05	1
MA1020.1	Phatr3_J14849	Phatr3_J1	230	237	+	12.0573	8.69E-05	1
MA1020.1	Phatr3_J14849	Phatr3_J1	326	333	-	12.0573	8.69E-05	1
MA1020.1	Phatr3_J14849	Phatr3_E0	197	204	-	12.0573	8.69E-05	1
MA1020.1	Phatr3_J14849	Phatr3_J4	655	662	+	12.0573	8.69E-05	1
MA1020.1	Phatr3_J14849	Phatr3_J3	918	925	+	12.0573	8.69E-05	1
MA1020.1	Phatr3_J14849	Phatr3_J7	398	405	+	12.0573	8.69E-05	1
MA1020.1	Phatr3_J14849	Phatr3_J4	420	427	-	12.0573	8.69E-05	1
MA1020.1	Phatr3_J14849	Phatr3_J5	901	908	+	12.0573	8.69E-05	1
MA1020.1	Phatr3_J14849	Phatr3_J4	819	826	-	12.0573	8.69E-05	1
MA1020.1	Phatr3_J14849	Phatr3_J1	1357	1364	+	12.0573	8.69E-05	1
MA1020.1	Phatr3_J14849	Phatr3_J2	725	732	+	12.0573	8.69E-05	1
MA1020.1	Phatr3_J14849	Phatr3_E0	798	805	+	12.0573	8.69E-05	1
MA1020.1	Phatr3_J14849	Phatr3_J4	708	715	-	12.0573	8.69E-05	1
MA1020.1	Phatr3_J14849	Phatr3_E0	525	532	-	12.0573	8.69E-05	1
MA1020.1	Phatr3_J14849	Phatr3_E0	11	18	-	12.0573	8.69E-05	1
MA1020.1	Phatr3_J14849	Phatr3_J4	387	394	-	12.0573	8.69E-05	1
MA1020.1	Phatr3_J14849	Phatr3_J4	1195	1202	+	12.0573	8.69E-05	1
MA1020.1	Phatr3_J14849	Phatr3_J3	679	686	+	12.0573	8.69E-05	1
MA1020.1	Phatr3_J14849	Phatr3_J5	1387	1394	+	12.0573	8.69E-05	1
MA1020.1	Phatr3_J14849	Phatr3_E0	1234	1241	-	12.0573	8.69E-05	1
MA1020.1	Phatr3_J14849	Phatr3_J4	1426	1433	-	12.0573	8.69E-05	1
MA1020.1	Phatr3_J14849	Phatr3_J1	682	689	+	12.0573	8.69E-05	1
MA1020.1	Phatr3_J14849	Phatr3_J1	680	687	-	12.0573	8.69E-05	1
MA1020.1	Phatr3_J14849	Phatr3_J5	1906	1913	+	12.0573	8.69E-05	1
MA1020.1	Phatr3_J14849	Phatr3_J4	163	170	+	12.0573	8.69E-05	1
MA1020.1	Phatr3_J14849	Phatr3_J4	829	836	+	12.0573	8.69E-05	1
MA1020.1	Phatr3_J14849	Phatr3_J1	1029	1036	+	12.0573	8.69E-05	1
MA1020.1	Phatr3_J14849	Phatr3_J4	1282	1289	+	12.0573	8.69E-05	1
MA1020.1	Phatr3_J14849	Phatr3_J1	986	993	-	12.0573	8.69E-05	1
MA1020.1	Phatr3_J14849	Phatr3_J4	754	761	+	12.0573	8.69E-05	1
MA1020.1	Phatr3_J14849	Phatr3_J4	1185	1192	+	12.0573	8.69E-05	1
MA1020.1	Phatr3_J14849	Phatr3_J4	841	848	-	12.0573	8.69E-05	1
MA1020.1	Phatr3_J14849	Phatr3_J4	1259	1266	+	12.0573	8.69E-05	1
MA1020.1	Phatr3_J14849	Phatr3_J4	766	773	+	12.0573	8.69E-05	1
MA1020.1	Phatr3_J14849	Phatr3_J3	1054	1061	+	12.0573	8.69E-05	1
MA1020.1	Phatr3_J14849	Phatr3_J4	789	796	+	12.0573	8.69E-05	1
MA1020.1	Phatr3_J14849	Phatr3_E0	1747	1754	-	12.0573	8.69E-05	1
MA1020.1	Phatr3_J14849	Phatr3_J1	186	193	+	12.0573	8.69E-05	1
MA1020.1	Phatr3_J14849	Phatr3_J4	1299	1306	-	12.0573	8.69E-05	1
MA1020.1	Phatr3_J14849	Phatr3_E0	1499	1506	+	12.0573	8.69E-05	1
MA1020.1	Phatr3_J14849	Phatr3_J5	1954	1961	+	12.0573	8.69E-05	1
MA1020.1	Phatr3_J14849	Phatr3_J5	983	990	-	12.0573	8.69E-05	1
MA1020.1	Phatr3_J14849	Phatr3_J5	993	1000	+	12.0573	8.69E-05	1
MA1020.1	Phatr3_J14849	Phatr3_J5	1790	1797	-	12.0573	8.69E-05	1

MA1020.1	Phatr3_J14849	Phatr3_J5	1137	1144	+	12.0573	8.69E-05	1
MA1020.1	Phatr3_J14849	Phatr3_J3	1088	1095	-	12.0573	8.69E-05	1
MA1020.1	Phatr3_J14849	Phatr3_J5	499	506	+	12.0573	8.69E-05	1
MA1020.1	Phatr3_J14849	Phatr3_EC	1747	1754	-	12.0573	8.69E-05	1
MA1020.1	Phatr3_J14849	Phatr3_J1	1014	1021	+	12.0573	8.69E-05	1
MA1020.1	Phatr3_J14849	Phatr3_J4	867	874	-	12.0573	8.69E-05	1
MA1020.1	Phatr3_J14849	Phatr3_EC	1747	1754	-	12.0573	8.69E-05	1
MA1020.1	Phatr3_J14849	Phatr3_J1	711	718	-	12.0573	8.69E-05	1
MA1020.1	Phatr3_J14849	Phatr3_J3	539	546	-	12.0573	8.69E-05	1
MA1020.1	Phatr3_J14849	Phatr3_J7	1682	1689	+	12.0573	8.69E-05	1
MA1020.1	Phatr3_J14849	Phatr3_J5	1654	1661	-	12.0573	8.69E-05	1
MA1020.1	Phatr3_J14849	Phatr3_J5	189	196	-	12.0573	8.69E-05	1
MA1020.1	Phatr3_J14849	Phatr3_J3	881	888	+	12.0573	8.69E-05	1
MA1020.1	Phatr3_J14849	Phatr3_J4	1608	1615	-	12.0573	8.69E-05	1
MA1020.1	Phatr3_J14849	Phatr3_J4	528	535	+	12.0573	8.69E-05	1
MA1020.1	Phatr3_J14849	Phatr3_J3	1284	1291	+	12.0573	8.69E-05	1
MA1020.1	Phatr3_J14849	Phatr3_J5	616	623	+	12.0573	8.69E-05	1
MA1020.1	Phatr3_J14849	Phatr3_J3	356	363	-	12.0573	8.69E-05	1
MA1020.1	Phatr3_J14849	Phatr3_J3	1402	1409	-	12.0573	8.69E-05	1
MA1020.1	Phatr3_J14849	Phatr3_J1	936	943	-	12.0573	8.69E-05	1
MA1020.1	Phatr3_J14849	Phatr3_J4	299	306	+	12.0573	8.69E-05	1
MA1020.1	Phatr3_J14849	Phatr3_J4	1890	1897	+	12.0573	8.69E-05	1
MA1020.1	Phatr3_J14849	Phatr3_J4	1067	1074	-	12.0573	8.69E-05	1
MA1020.1	Phatr3_J14849	Phatr3_J4	1692	1699	+	12.0573	8.69E-05	1
MA1020.1	Phatr3_J14849	Phatr3_J3	805	812	-	12.0573	8.69E-05	1
MA1020.1	Phatr3_J14849	Phatr3_EC	675	682	-	12.0573	8.69E-05	1
MA1020.1	Phatr3_J14849	Phatr3_J1	781	788	-	12.0573	8.69E-05	1
MA1020.1	Phatr3_J14849	Phatr3_J1	715	722	+	12.0573	8.69E-05	1
MA1020.1	Phatr3_J14849	Phatr3_J3	258	265	+	12.0573	8.69E-05	1
MA1020.1	Phatr3_J14849	Phatr3_J3	403	410	-	12.0573	8.69E-05	1
MA1020.1	Phatr3_J14849	Phatr3_J3	172	179	+	12.0573	8.69E-05	1
MA1020.1	Phatr3_J14849	Phatr3_J3	801	808	+	12.0573	8.69E-05	1
MA1020.1	Phatr3_J14849	Phatr3_EC	1215	1222	+	12.0573	8.69E-05	1
MA1020.1	Phatr3_J14849	Phatr3_EC	46	53	-	12.0573	8.69E-05	1
MA1020.1	Phatr3_J14849	Phatr3_J4	184	191	-	12.0573	8.69E-05	1
MA1020.1	Phatr3_J14849	Phatr3_J3	1637	1644	-	12.0573	8.69E-05	1
MA1020.1	Phatr3_J14849	Phatr3_J4	911	918	-	12.0573	8.69E-05	1
MA1020.1	Phatr3_J14849	Phatr3_J5	1993	2000	-	12.0573	8.69E-05	1
MA1020.1	Phatr3_J14849	Phatr3_J3	972	979	+	12.0573	8.69E-05	1
MA1020.1	Phatr3_J14849	Phatr3_J3	1913	1920	-	12.0573	8.69E-05	1
MA1020.1	Phatr3_J14849	Phatr3_J5	1888	1895	-	12.0573	8.69E-05	1
MA1020.1	Phatr3_J14849	Phatr3_EC	1950	1957	-	12.0573	8.69E-05	1
MA1020.1	Phatr3_J14849	Phatr3_J4	836	843	-	12.0573	8.69E-05	1
MA1020.1	Phatr3_J14849	Phatr3_EC	523	530	-	12.0573	8.69E-05	1
MA1020.1	Phatr3_J14849	Phatr3_J2	990	997	+	12.0573	8.69E-05	1
MA1020.1	Phatr3_J14849	Phatr3_J4	1001	1008	+	12.0573	8.69E-05	1
MA1020.1	Phatr3_J14849	Phatr3_J4	71	78	+	12.0573	8.69E-05	1
MA1020.1	Phatr3_J14849	Phatr3_J4	1644	1651	-	12.0573	8.69E-05	1
MA1020.1	Phatr3_J14849	Phatr3_J3	732	739	+	12.0573	8.69E-05	1
MA1020.1	Phatr3_J14849	Phatr3_J7	755	762	+	12.0573	8.69E-05	1
MA1020.1	Phatr3_J14849	Phatr3_EC	521	528	+	12.0573	8.69E-05	1
MA1020.1	Phatr3_J14849	Phatr3_J3	255	262	-	12.0573	8.69E-05	1
MA1020.1	Phatr3_J14849	Phatr3_J5	1184	1191	+	12.0573	8.69E-05	1
MA1020.1	Phatr3_J14849	Phatr3_J1	514	521	-	12.0573	8.69E-05	1

MA1020.1	Phatr3_J14849	Phatr3_J7	591	598	+	14.0632	1.79E-05	1
MA1020.1	Phatr3_J14849	Phatr3_J3	1204	1211	+	14.0632	1.79E-05	1
MA1020.1	Phatr3_J14849	Phatr3_J5	1834	1841	-	14.0632	1.79E-05	1
MA1020.1	Phatr3_J14849	Phatr3_J1	646	653	+	14.0632	1.79E-05	1
MA1020.1	Phatr3_J14849	Phatr3_J8	1510	1517	+	14.0632	1.79E-05	1
MA1020.1	Phatr3_J14849	Phatr3_J3	768	775	+	14.0632	1.79E-05	1
MA1020.1	Phatr3_J14849	PTsRNA00C	950	957	+	14.0632	1.79E-05	1
MA1020.1	Phatr3_J14849	Phatr3_J3	995	1002	+	14.0632	1.79E-05	1
MA1020.1	Phatr3_J14849	Phatr3_J4	1038	1045	-	14.0632	1.79E-05	1
MA1020.1	Phatr3_J14849	Phatr3_EC	809	816	+	14.0632	1.79E-05	1
MA1020.1	Phatr3_J14849	Phatr3_EC	807	814	-	14.0632	1.79E-05	1
MA1020.1	Phatr3_J14849	PTsRNA00C	131	138	+	14.0632	1.79E-05	1
MA1020.1	Phatr3_J14849	PTsRNA00C	185	192	+	14.0632	1.79E-05	1
MA1020.1	Phatr3_J14849	Phatr3_J1	1001	1008	+	14.0632	1.79E-05	1
MA1020.1	Phatr3_J14849	Phatr3_EC	118	125	-	14.0632	1.79E-05	1
MA1020.1	Phatr3_J14849	Phatr3_J4	1680	1687	+	14.0632	1.79E-05	1
MA1020.1	Phatr3_J14849	Phatr3_J8	1915	1922	-	14.0632	1.79E-05	1
MA1020.1	Phatr3_J14849	Phatr3_J5	1237	1244	+	14.0632	1.79E-05	1
MA1020.1	Phatr3_J14849	Phatr3_J3	56	63	-	14.0632	1.79E-05	1
MA1020.1	Phatr3_J14849	Phatr3_J4	1554	1561	-	14.0632	1.79E-05	1
MA1020.1	Phatr3_J14849	Phatr3_J3	540	547	-	14.0632	1.79E-05	1
MA1020.1	Phatr3_J14849	Phatr3_J4	1641	1648	-	14.0632	1.79E-05	1
MA1020.1	Phatr3_J14849	Phatr3_J4	1977	1984	+	14.0632	1.79E-05	1
MA1020.1	Phatr3_J14849	Phatr3_J3	1834	1841	+	14.0632	1.79E-05	1
MA1020.1	Phatr3_J14849	Phatr3_J4	1757	1764	-	14.0632	1.79E-05	1
MA1020.1	Phatr3_J14849	Phatr3_J4	851	858	-	14.0632	1.79E-05	1
MA1020.1	Phatr3_J14849	Phatr3_J4	577	584	+	14.0632	1.79E-05	1
MA1020.1	Phatr3_J14849	Phatr3_J4	817	824	-	14.0632	1.79E-05	1
MA1020.1	Phatr3_J14849	Phatr3_J3	492	499	+	14.0632	1.79E-05	1
MA1020.1	Phatr3_J14849	Phatr3_J1	60	67	+	14.0632	1.79E-05	1
MA1020.1	Phatr3_J14849	Phatr3_EC	17	24	-	14.0632	1.79E-05	1
MA1020.1	Phatr3_J14849	Phatr3_EC	43	50	-	14.0632	1.79E-05	1
MA1020.1	Phatr3_J14849	Phatr3_J2	1743	1750	+	14.0632	1.79E-05	1
MA1020.1	Phatr3_J14849	Phatr3_J3	1040	1047	+	14.0632	1.79E-05	1
MA1020.1	Phatr3_J14849	Phatr3_EC	1226	1233	+	14.0632	1.79E-05	1
MA1020.1	Phatr3_J14849	Phatr3_EC	608	615	+	14.0632	1.79E-05	1
MA1020.1	Phatr3_J14849	Phatr3_J2	303	310	+	14.0632	1.79E-05	1
MA1020.1	Phatr3_J14849	Phatr3_J4	1916	1923	-	14.0632	1.79E-05	1
MA1020.1	Phatr3_J14849	Phatr3_J2	856	863	+	14.0632	1.79E-05	1
MA1020.1	Phatr3_J14849	Phatr3_J3	1904	1911	-	14.0632	1.79E-05	1
MA1020.1	Phatr3_J14849	Phatr3_J4	1043	1050	+	14.0632	1.79E-05	1
MA1020.1	Phatr3_J14849	Phatr3_EC	736	743	-	14.0632	1.79E-05	1
MA1020.1	Phatr3_J14849	Phatr3_J4	1044	1051	-	14.0632	1.79E-05	1
MA1020.1	Phatr3_J14849	Phatr3_J4	915	922	+	14.0632	1.79E-05	1
MA1020.1	Phatr3_J14849	Phatr3_J3	1898	1905	-	14.0632	1.79E-05	1
MA1020.1	Phatr3_J14849	Phatr3_J2	810	817	-	14.0632	1.79E-05	1
MA1020.1	Phatr3_J14849	Phatr3_J4	926	933	+	14.0632	1.79E-05	1
MA1020.1	Phatr3_J14849	Phatr3_J4	1898	1905	-	14.0632	1.79E-05	1
MA1020.1	Phatr3_J14849	Phatr3_J1	276	283	-	14.0632	1.79E-05	1
MA1020.1	Phatr3_J14849	Phatr3_J3	1808	1815	+	14.0632	1.79E-05	1
MA1020.1	Phatr3_J14849	Phatr3_J4	1094	1101	+	14.0632	1.79E-05	1
MA1020.1	Phatr3_J14849	Phatr3_J4	1520	1527	-	14.0632	1.79E-05	1
MA1020.1	Phatr3_J14849	Phatr3_J2	1381	1388	-	14.0632	1.79E-05	1
MA1020.1	Phatr3_J14849	Phatr3_J5	1230	1237	-	14.0632	1.79E-05	1

MA1020.1	Phatr3_J14849	Phatr3_EC	1647	1654	-	14.0632	1.79E-05	1
MA1020.1	Phatr3_J14849	Phatr3_J1	1237	1244	+	14.0632	1.79E-05	1
MA1020.1	Phatr3_J14849	Phatr3_EC	1443	1450	-	14.0632	1.79E-05	1
MA1020.1	Phatr3_J14849	Phatr3_J4	1576	1583	+	14.0632	1.79E-05	1
MA1020.1	Phatr3_J14849	Phatr3_EC	1957	1964	-	14.0632	1.79E-05	1
MA1020.1	Phatr3_J14849	Phatr3_J4	193	200	-	14.0632	1.79E-05	1
MA1020.1	Phatr3_J14849	Phatr3_J4	932	939	+	14.0632	1.79E-05	1
MA1020.1	Phatr3_J14849	Phatr3_J2	209	216	+	14.0632	1.79E-05	1
MA1020.1	Phatr3_J14849	Phatr3_J2	207	214	-	14.0632	1.79E-05	1
MA1020.1	Phatr3_J14849	Phatr3_J2	910	917	+	14.0632	1.79E-05	1
MA1020.1	Phatr3_J14849	Phatr3_J4	581	588	+	14.0632	1.79E-05	1
MA1020.1	Phatr3_J14849	Phatr3_EC	373	380	-	14.0632	1.79E-05	1
MA1020.1	Phatr3_J14849	Phatr3_J4	571	578	-	14.0632	1.79E-05	1
MA1020.1	Phatr3_J14849	Phatr3_J3	1689	1696	+	14.0632	1.79E-05	1
MA1020.1	Phatr3_J14849	Phatr3_J4	1830	1837	+	14.0632	1.79E-05	1
MA1020.1	Phatr3_J14849	Phatr3_J4	1071	1078	-	14.0632	1.79E-05	1
MA1020.1	Phatr3_J14849	Phatr3_J4	1652	1659	-	14.0632	1.79E-05	1
MA1020.1	Phatr3_J14849	Phatr3_J3	1619	1626	+	14.0632	1.79E-05	1
MA1020.1	Phatr3_J14849	Phatr3_J3	1733	1740	+	14.0632	1.79E-05	1
MA1020.1	Phatr3_J14849	Phatr3_J4	971	978	-	14.0632	1.79E-05	1
MA1020.1	Phatr3_J14849	Phatr3_J4	1359	1366	-	14.0632	1.79E-05	1
MA1020.1	Phatr3_J14849	Phatr3_J3	205	212	+	14.0632	1.79E-05	1
MA1020.1	Phatr3_J14849	Phatr3_J3	593	600	+	14.0632	1.79E-05	1
MA1020.1	Phatr3_J14849	Phatr3_J2	1219	1226	+	14.0632	1.79E-05	1
MA1020.1	Phatr3_J14849	Phatr3_EC	1199	1206	+	14.0632	1.79E-05	1
MA1020.1	Phatr3_J14849	Phatr3_EC	907	914	+	14.0632	1.79E-05	1
MA1020.1	Phatr3_J14849	Phatr3_J1	221	228	+	14.0632	1.79E-05	1
MA1020.1	Phatr3_J14849	Phatr3_J5	92	99	+	14.0632	1.79E-05	1
MA1020.1	Phatr3_J14849	Phatr3_EC	809	816	-	14.0632	1.79E-05	1
MA1020.1	Phatr3_J14849	Phatr3_J4	1992	1999	+	14.0632	1.79E-05	1
MA1020.1	Phatr3_J14849	Phatr3_J4	63	70	+	14.0632	1.79E-05	1
MA1020.1	Phatr3_J14849	Phatr3_J4	542	549	+	14.0632	1.79E-05	1
MA1020.1	Phatr3_J14849	Phatr3_EC	1260	1267	+	14.0632	1.79E-05	1
MA1020.1	Phatr3_J14849	Phatr3_J4	1068	1075	+	14.0632	1.79E-05	1
MA1020.1	Phatr3_J14849	Phatr3_J4	463	470	+	14.0632	1.79E-05	1
MA1020.1	Phatr3_J14849	Phatr3_J4	304	311	+	14.0632	1.79E-05	1
MA1020.1	Phatr3_J14849	Phatr3_J3	1704	1711	+	14.0632	1.79E-05	1
MA1020.1	Phatr3_J14849	Phatr3_J4	5	12	+	14.0632	1.79E-05	1
MA1020.1	Phatr3_J14849	Phatr3_J2	554	561	+	14.0632	1.79E-05	1
MA1020.1	Phatr3_J14849	Phatr3_EC	1638	1645	-	14.0632	1.79E-05	1
MA1020.1	Phatr3_J14849	Phatr3_J3	1718	1725	-	14.0632	1.79E-05	1
MA1020.1	Phatr3_J14849	Phatr3_J3	341	348	+	14.0632	1.79E-05	1
MA1020.1	Phatr3_J14849	Phatr3_J3	1814	1821	+	14.0632	1.79E-05	1
MA1020.1	Phatr3_J14849	Phatr3_J4	1658	1665	+	14.0632	1.79E-05	1
MA1020.1	Phatr3_J14849	Phatr3_EC	1401	1408	+	14.0632	1.79E-05	1
MA1020.1	Phatr3_J14849	Phatr3_J1	1962	1969	+	14.0632	1.79E-05	1
MA1020.1	Phatr3_J14849	Phatr3_J4	1391	1398	+	14.0632	1.79E-05	1
MA1020.1	Phatr3_J14849	Phatr3_J4	1389	1396	-	14.0632	1.79E-05	1
MA1020.1	Phatr3_J14849	Phatr3_J5	86	93	+	14.0632	1.79E-05	1
MA1020.1	Phatr3_J14849	Phatr3_J5	84	91	-	14.0632	1.79E-05	1
MA1020.1	Phatr3_J14849	Phatr3_J3	923	930	-	14.0632	1.79E-05	1
MA1020.1	Phatr3_J14849	Phatr3_J4	152	159	+	14.0632	1.79E-05	1
MA1020.1	Phatr3_J14849	Phatr3_J4	810	817	+	14.0632	1.79E-05	1
MA1020.1	Phatr3_J14849	Phatr3_J3	1697	1704	-	14.0632	1.79E-05	1

MA1020.1	Phatr3_J14849	Phatr3_J1	1925	1932	-	14.0632	1.79E-05	1
MA1020.1	Phatr3_J14849	Phatr3_J4	1473	1480	+	14.0632	1.79E-05	1
MA1020.1	Phatr3_J14849	EPrPhatr3	964	971	+	14.0632	1.79E-05	1
MA1020.1	Phatr3_J14849	Phatr3_J4	1845	1852	+	14.0632	1.79E-05	1
MA1020.1	Phatr3_J14849	Phatr3_J4	1843	1850	-	14.0632	1.79E-05	1
MA1020.1	Phatr3_J14849	Phatr3_J4	1343	1350	+	14.0632	1.79E-05	1
MA1020.1	Phatr3_J14849	Phatr3_J4	1341	1348	-	14.0632	1.79E-05	1
MA1020.1	Phatr3_J14849	Phatr3_J1	1296	1303	-	14.0632	1.79E-05	1
MA1020.1	Phatr3_J14849	Phatr3_EC	1234	1241	-	14.0632	1.79E-05	1
MA1020.1	Phatr3_J14849	Phatr3_J4	688	695	+	14.0632	1.79E-05	1
MA1020.1	Phatr3_J14849	Phatr3_J1	1730	1737	-	14.0632	1.79E-05	1
MA1020.1	Phatr3_J14849	Phatr3_J4	728	735	-	14.0632	1.79E-05	1
MA1020.1	Phatr3_J14849	Phatr3_EC	119	126	-	14.0632	1.79E-05	1
MA1020.1	Phatr3_J14849	Phatr3_J4	1536	1543	-	14.0632	1.79E-05	1
MA1020.1	Phatr3_J14849	Phatr3_J5	1859	1866	+	14.0632	1.79E-05	1
MA1020.1	Phatr3_J14849	Phatr3_J1	1428	1435	+	14.0632	1.79E-05	1
MA1020.1	Phatr3_J14849	Phatr3_EC	1973	1980	+	14.0632	1.79E-05	1
MA1020.1	Phatr3_J14849	Phatr3_J4	1443	1450	+	14.0632	1.79E-05	1
MA1020.1	Phatr3_J14849	Phatr3_EC	770	777	-	14.0632	1.79E-05	1
MA1020.1	Phatr3_J14849	Phatr3_J4	428	435	+	14.0632	1.79E-05	1
MA1020.1	Phatr3_J14849	Phatr3_J4	1382	1389	-	14.0632	1.79E-05	1
MA1020.1	Phatr3_J14849	Phatr3_EC	857	864	-	14.0632	1.79E-05	1
MA1020.1	Phatr3_J14849	Phatr3_J2	1819	1826	+	14.0632	1.79E-05	1
MA1020.1	Phatr3_J14849	Phatr3_J4	1460	1467	+	14.0632	1.79E-05	1
MA1020.1	Phatr3_J14849	Phatr3_J4	1012	1019	-	14.0632	1.79E-05	1
MA1020.1	Phatr3_J14849	Phatr3_J4	1429	1436	+	14.0632	1.79E-05	1
MA1020.1	Phatr3_J14849	Phatr3_J5	409	416	-	14.0632	1.79E-05	1
MA1020.1	Phatr3_J14849	Phatr3_EC	34	41	+	14.0632	1.79E-05	1
MA1020.1	Phatr3_J14849	Phatr3_EC	1125	1132	+	14.0632	1.79E-05	1
MA1020.1	Phatr3_J14849	Phatr3_J5	625	632	-	14.0632	1.79E-05	1
MA1020.1	Phatr3_J14849	Phatr3_J1	556	563	+	14.0632	1.79E-05	1
MA1020.1	Phatr3_J14849	Phatr3_J5	1643	1650	+	14.0632	1.79E-05	1
MA1020.1	Phatr3_J14849	Phatr3_J5	1641	1648	-	14.0632	1.79E-05	1
MA1020.1	Phatr3_J14849	Phatr3_J5	342	349	+	14.0632	1.79E-05	1
MA1020.1	Phatr3_J14849	Phatr3_J5	340	347	-	14.0632	1.79E-05	1
MA1020.1	Phatr3_J14849	Phatr3_EC	1906	1913	-	14.0632	1.79E-05	1
MA1020.1	Phatr3_J14849	Phatr3_EC	10	17	+	14.0632	1.79E-05	1
MA1020.1	Phatr3_J14849	Phatr3_J5	698	705	+	14.0632	1.79E-05	1
MA1020.1	Phatr3_J14849	Phatr3_J5	507	514	-	14.0632	1.79E-05	1
MA1020.1	Phatr3_J14849	Phatr3_J2	437	444	+	14.0632	1.79E-05	1
MA1020.1	Phatr3_J14849	Phatr3_EC	348	355	-	14.0632	1.79E-05	1
MA1020.1	Phatr3_J14849	Phatr3_J4	426	433	+	14.0632	1.79E-05	1
MA1020.1	Phatr3_J14849	Phatr3_EC	447	454	-	14.0632	1.79E-05	1
MA1020.1	Phatr3_J14849	EPrPhatr3	893	900	+	14.0632	1.79E-05	1
MA1020.1	Phatr3_J14849	PTsRNA00C	840	847	+	14.0632	1.79E-05	1
MA1020.1	Phatr3_J14849	Phatr3_EC	1602	1609	+	14.0632	1.79E-05	1
MA1020.1	Phatr3_J14849	Phatr3_J8	335	342	+	14.0632	1.79E-05	1
MA1020.1	Phatr3_J14849	Phatr3_J4	1423	1430	-	14.0632	1.79E-05	1
MA1020.1	Phatr3_J14849	Phatr3_J5	1350	1357	+	14.0632	1.79E-05	1
MA1020.1	Phatr3_J14849	Phatr3_EC	253	260	-	14.0632	1.79E-05	1
MA1020.1	Phatr3_J14849	Phatr3_EC	1301	1308	-	14.0632	1.79E-05	1
MA1020.1	Phatr3_J14849	Phatr3_J5	1589	1596	+	14.0632	1.79E-05	1
MA1020.1	Phatr3_J14849	Phatr3_EC	1928	1935	-	14.0632	1.79E-05	1
MA1020.1	Phatr3_J14849	Phatr3_J4	1487	1494	+	14.0632	1.79E-05	1

MA1020.1	Phatr3_J14849	Phatr3_EC	339	346	-	14.0632	1.79E-05	1
MA1020.1	Phatr3_J14849	Phatr3_J4	990	997	-	14.0632	1.79E-05	1
MA1020.1	Phatr3_J14849	Phatr3_J5	1710	1717	+	14.0632	1.79E-05	1
MA1020.1	Phatr3_J14849	Phatr3_J3	825	832	+	14.0632	1.79E-05	1
MA1020.1	Phatr3_J14849	Phatr3_J3	1019	1026	+	14.0632	1.79E-05	1
MA1020.1	Phatr3_J14849	Phatr3_J4	632	639	-	14.0632	1.79E-05	1
MA1020.1	Phatr3_J14849	Phatr3_J3	1393	1400	+	14.0632	1.79E-05	1
MA1020.1	Phatr3_J14849	Phatr3_J1	1779	1786	-	14.0632	1.79E-05	1
MA1020.1	Phatr3_J14849	Phatr3_EC	1378	1385	-	14.0632	1.79E-05	1
MA1020.1	Phatr3_J14849	Phatr3_EC	1974	1981	+	14.0632	1.79E-05	1
MA1020.1	Phatr3_J14849	Phatr3_J3	1745	1752	+	14.0632	1.79E-05	1
MA1020.1	Phatr3_J14849	Phatr3_J4	1390	1397	-	14.0632	1.79E-05	1
MA1020.1	Phatr3_J14849	Phatr3_J4	1522	1529	+	14.0632	1.79E-05	1
MA1020.1	Phatr3_J14849	Phatr3_EC	1607	1614	-	14.0632	1.79E-05	1
MA1020.1	Phatr3_J14849	Phatr3_EC	1508	1515	+	14.0632	1.79E-05	1
MA1020.1	Phatr3_J14849	Phatr3_J4	111	118	-	14.0632	1.79E-05	1
MA1020.1	Phatr3_J14849	Phatr3_J3	7	14	-	14.0632	1.79E-05	1
MA1020.1	Phatr3_J14849	Phatr3_EC	324	331	+	14.0632	1.79E-05	1
MA1020.1	Phatr3_J14849	Phatr3_EC	10	17	+	14.0632	1.79E-05	1
MA1020.1	Phatr3_J14849	Phatr3_EC	1906	1913	-	14.0632	1.79E-05	1
MA1020.1	Phatr3_J14849	Phatr3_EC	398	405	-	14.0632	1.79E-05	1
MA1020.1	Phatr3_J14849	Phatr3_J4	505	512	-	14.0632	1.79E-05	1
MA1020.1	Phatr3_J14849	Phatr3_J5	1211	1218	-	14.0632	1.79E-05	1
MA1020.1	Phatr3_J14849	Phatr3_EC	793	800	+	14.0632	1.79E-05	1
MA1020.1	Phatr3_J14849	Phatr3_EC	791	798	-	14.0632	1.79E-05	1
MA1020.1	Phatr3_J14849	Phatr3_EC	1853	1860	+	14.0632	1.79E-05	1
MA1020.1	Phatr3_J14849	Phatr3_EC	1851	1858	-	14.0632	1.79E-05	1
MA1020.1	Phatr3_J14849	Phatr3_J4	301	308	+	14.0632	1.79E-05	1
MA1020.1	Phatr3_J14849	Phatr3_J3	801	808	-	14.0632	1.79E-05	1
MA1020.1	Phatr3_J14849	Phatr3_J4	1123	1130	-	14.0632	1.79E-05	1
MA1020.1	Phatr3_J14849	Phatr3_J4	708	715	-	14.0632	1.79E-05	1
MA1020.1	Phatr3_J14849	Phatr3_J4	1236	1243	+	14.0632	1.79E-05	1
MA1020.1	Phatr3_J14849	Phatr3_J4	307	314	+	14.0632	1.79E-05	1
MA1020.1	Phatr3_J14849	Phatr3_J4	214	221	+	14.0632	1.79E-05	1
MA1020.1	Phatr3_J14849	Phatr3_J2	635	642	-	14.0632	1.79E-05	1
MA1020.1	Phatr3_J14849	Phatr3_J4	372	379	-	14.0632	1.79E-05	1
MA1020.1	Phatr3_J14849	Phatr3_J4	1748	1755	+	14.0632	1.79E-05	1
MA1020.1	Phatr3_J14849	Phatr3_J4	1632	1639	-	14.0632	1.79E-05	1
MA1020.1	Phatr3_J14849	Phatr3_J3	749	756	-	12.5202	3.26E-05	1
MA1020.1	Phatr3_J14849	Phatr3_J1	101	108	-	12.5202	3.26E-05	1
MA1020.1	Phatr3_J14849	Phatr3_J4	1269	1276	-	12.5202	3.26E-05	1
MA1020.1	Phatr3_J14849	Phatr3_J2	705	712	+	12.5202	3.26E-05	1
MA1020.1	Phatr3_J14849	Phatr3_J4	1590	1597	-	12.5202	3.26E-05	1
MA1020.1	Phatr3_J14849	Phatr3_J9	1848	1855	+	12.5202	3.26E-05	1
MA1020.1	Phatr3_J14849	Phatr3_EC	663	670	+	12.5202	3.26E-05	1
MA1020.1	Phatr3_J14849	Phatr3_J4	1565	1572	-	12.5202	3.26E-05	1
MA1020.1	Phatr3_J14849	Phatr3_J5	1988	1995	+	12.5202	3.26E-05	1
MA1020.1	Phatr3_J14849	Phatr3_EC	1111	1118	-	12.5202	3.26E-05	1
MA1020.1	Phatr3_J14849	Phatr3_EC	544	551	-	12.5202	3.26E-05	1
MA1020.1	Phatr3_J14849	Phatr3_J9	1643	1650	-	12.5202	3.26E-05	1
MA1020.1	Phatr3_J14849	Phatr3_J3	738	745	+	12.5202	3.26E-05	1
MA1020.1	Phatr3_J14849	Phatr3_J4	1951	1958	+	12.5202	3.26E-05	1
MA1020.1	Phatr3_J14849	Phatr3_J4	1183	1190	-	12.5202	3.26E-05	1
MA1020.1	Phatr3_J14849	Phatr3_J1	1321	1328	-	12.5202	3.26E-05	1

MA1020.1	Phatr3_J14849	PTsRNA00(	1948	1955	-	12.5202	3.26E-05	1
MA1020.1	Phatr3_J14849	PTsRNA00(	1913	1920	-	12.5202	3.26E-05	1
MA1020.1	Phatr3_J14849	EPrPhatr3	1974	1981	-	12.5202	3.26E-05	1
MA1020.1	Phatr3_J14849	Phatr3_J3	428	435	+	12.5202	3.26E-05	1
MA1020.1	Phatr3_J14849	Phatr3_J3	542	549	+	12.5202	3.26E-05	1
MA1020.1	Phatr3_J14849	Phatr3_J3	1245	1252	-	12.5202	3.26E-05	1
MA1020.1	Phatr3_J14849	Phatr3_J4	20	27	+	12.5202	3.26E-05	1
MA1020.1	Phatr3_J14849	Phatr3_J2	1567	1574	-	12.5202	3.26E-05	1
MA1020.1	Phatr3_J14849	Phatr3_J3	1755	1762	+	12.5202	3.26E-05	1
MA1020.1	Phatr3_J14849	Phatr3_E(	1240	1247	+	12.5202	3.26E-05	1
MA1020.1	Phatr3_J14849	Phatr3_J4	1439	1446	-	12.5202	3.26E-05	1
MA1020.1	Phatr3_J14849	Phatr3_J4	1651	1658	-	12.5202	3.26E-05	1
MA1020.1	Phatr3_J14849	Phatr3_E(	217	224	+	12.5202	3.26E-05	1
MA1020.1	Phatr3_J14849	Phatr3_J3	1489	1496	+	12.5202	3.26E-05	1
MA1020.1	Phatr3_J14849	Phatr3_J2	1806	1813	-	12.5202	3.26E-05	1
MA1020.1	Phatr3_J14849	Phatr3_J4	97	104	-	12.5202	3.26E-05	1
MA1020.1	Phatr3_J14849	Phatr3_J4	888	895	-	12.5202	3.26E-05	1
MA1020.1	Phatr3_J14849	Phatr3_J3	873	880	-	12.5202	3.26E-05	1
MA1020.1	Phatr3_J14849	Phatr3_J2	1759	1766	-	12.5202	3.26E-05	1
MA1020.1	Phatr3_J14849	Phatr3_J4	1053	1060	-	12.5202	3.26E-05	1
MA1020.1	Phatr3_J14849	Phatr3_J3	1806	1813	+	12.5202	3.26E-05	1
MA1020.1	Phatr3_J14849	Phatr3_J3	1953	1960	-	12.5202	3.26E-05	1
MA1020.1	Phatr3_J14849	Phatr3_J2	1255	1262	+	12.5202	3.26E-05	1
MA1020.1	Phatr3_J14849	Phatr3_J4	1449	1456	-	12.5202	3.26E-05	1
MA1020.1	Phatr3_J14849	Phatr3_J5	1782	1789	+	12.5202	3.26E-05	1
MA1020.1	Phatr3_J14849	Phatr3_E(	1118	1125	+	12.5202	3.26E-05	1
MA1020.1	Phatr3_J14849	Phatr3_E(	1814	1821	+	12.5202	3.26E-05	1
MA1020.1	Phatr3_J14849	Phatr3_J4	1432	1439	-	12.5202	3.26E-05	1
MA1020.1	Phatr3_J14849	Phatr3_J1	1537	1544	-	12.5202	3.26E-05	1
MA1020.1	Phatr3_J14849	Phatr3_J4	1961	1968	+	12.5202	3.26E-05	1
MA1020.1	Phatr3_J14849	Phatr3_J3	1610	1617	-	12.5202	3.26E-05	1
MA1020.1	Phatr3_J14849	Phatr3_J3	1358	1365	+	12.5202	3.26E-05	1
MA1020.1	Phatr3_J14849	Phatr3_J4	576	583	-	12.5202	3.26E-05	1
MA1020.1	Phatr3_J14849	Phatr3_J4	1116	1123	-	12.5202	3.26E-05	1
MA1020.1	Phatr3_J14849	Phatr3_J4	1846	1853	+	12.5202	3.26E-05	1
MA1020.1	Phatr3_J14849	Phatr3_J1	1321	1328	-	12.5202	3.26E-05	1
MA1020.1	Phatr3_J14849	Phatr3_J3	1904	1911	-	12.5202	3.26E-05	1
MA1020.1	Phatr3_J14849	Phatr3_J1	1425	1432	-	12.5202	3.26E-05	1
MA1020.1	Phatr3_J14849	Phatr3_J3	1821	1828	-	12.5202	3.26E-05	1
MA1020.1	Phatr3_J14849	Phatr3_J4	1132	1139	+	12.5202	3.26E-05	1
MA1020.1	Phatr3_J14849	Phatr3_E(	484	491	-	12.5202	3.26E-05	1
MA1020.1	Phatr3_J14849	Phatr3_J2	558	565	-	12.5202	3.26E-05	1
MA1020.1	Phatr3_J14849	Phatr3_J1	219	226	-	12.5202	3.26E-05	1
MA1020.1	Phatr3_J14849	Phatr3_J4	1889	1896	+	12.5202	3.26E-05	1
MA1020.1	Phatr3_J14849	Phatr3_E(	306	313	-	12.5202	3.26E-05	1
MA1020.1	Phatr3_J14849	Phatr3_E(	664	671	+	12.5202	3.26E-05	1
MA1020.1	Phatr3_J14849	Phatr3_E(	811	818	+	12.5202	3.26E-05	1
MA1020.1	Phatr3_J14849	Phatr3_J3	38	45	-	12.5202	3.26E-05	1
MA1020.1	Phatr3_J14849	Phatr3_E(	1005	1012	-	12.5202	3.26E-05	1
MA1020.1	Phatr3_J14849	Phatr3_J3	1578	1585	+	12.5202	3.26E-05	1
MA1020.1	Phatr3_J14849	Phatr3_J4	1291	1298	-	12.5202	3.26E-05	1
MA1020.1	Phatr3_J14849	Phatr3_J4	68	75	-	12.5202	3.26E-05	1
MA1020.1	Phatr3_J14849	Phatr3_J4	1761	1768	+	12.5202	3.26E-05	1
MA1020.1	Phatr3_J14849	Phatr3_J4	390	397	+	12.5202	3.26E-05	1

MA1020.1	Phatr3_J14849	Phatr3_J1	373	380	-	12.5202	3.26E-05	1
MA1020.1	Phatr3_J14849	Phatr3_J4	858	865	-	12.5202	3.26E-05	1
MA1020.1	Phatr3_J14849	Phatr3_E(	1877	1884	-	12.5202	3.26E-05	1
MA1020.1	Phatr3_J14849	Phatr3_J4	62	69	+	12.5202	3.26E-05	1
MA1020.1	Phatr3_J14849	Phatr3_J5	1857	1864	-	12.5202	3.26E-05	1
MA1020.1	Phatr3_J14849	Phatr3_J3	1778	1785	-	12.5202	3.26E-05	1
MA1020.1	Phatr3_J14849	Phatr3_E(	371	378	+	12.5202	3.26E-05	1
MA1020.1	Phatr3_J14849	Phatr3_E(	211	218	-	12.5202	3.26E-05	1
MA1020.1	Phatr3_J14849	Phatr3_J4	1574	1581	-	12.5202	3.26E-05	1
MA1020.1	Phatr3_J14849	Phatr3_J4	1408	1415	+	12.5202	3.26E-05	1
MA1020.1	Phatr3_J14849	Phatr3_J4	1825	1832	-	12.5202	3.26E-05	1
MA1020.1	Phatr3_J14849	Phatr3_J1	848	855	-	12.5202	3.26E-05	1
MA1020.1	Phatr3_J14849	Phatr3_E(	32	39	-	12.5202	3.26E-05	1
MA1020.1	Phatr3_J14849	Phatr3_E(	179	186	-	12.5202	3.26E-05	1
MA1020.1	Phatr3_J14849	Phatr3_E(	1123	1130	-	12.5202	3.26E-05	1
MA1020.1	Phatr3_J14849	Phatr3_E(	1270	1277	-	12.5202	3.26E-05	1
MA1020.1	Phatr3_J14849	Phatr3_E(	1789	1796	+	12.5202	3.26E-05	1
MA1020.1	Phatr3_J14849	Phatr3_E(	279	286	+	12.5202	3.26E-05	1
MA1020.1	Phatr3_J14849	Phatr3_J3	1263	1270	+	12.5202	3.26E-05	1
MA1020.1	Phatr3_J14849	Phatr3_E(	600	607	-	12.5202	3.26E-05	1
MA1020.1	Phatr3_J14849	Phatr3_J4	1604	1611	+	12.5202	3.26E-05	1
MA1020.1	Phatr3_J14849	Phatr3_J5	1060	1067	+	12.5202	3.26E-05	1
MA1020.1	Phatr3_J14849	Phatr3_J4	91	98	-	12.5202	3.26E-05	1
MA1020.1	Phatr3_J14849	Phatr3_J2	1448	1455	-	12.5202	3.26E-05	1
MA1020.1	Phatr3_J14849	Phatr3_J4	833	840	-	12.5202	3.26E-05	1
MA1020.1	Phatr3_J14849	Phatr3_J4	1104	1111	+	12.5202	3.26E-05	1
MA1020.1	Phatr3_J14849	Phatr3_J3	658	665	-	12.5202	3.26E-05	1
MA1020.1	Phatr3_J14849	Phatr3_E(	1111	1118	-	12.5202	3.26E-05	1
MA1020.1	Phatr3_J14849	Phatr3_J8	333	340	-	12.5202	3.26E-05	1
MA1020.1	Phatr3_J14849	Phatr3_J4	1425	1432	+	12.5202	3.26E-05	1
MA1020.1	Phatr3_J14849	Phatr3_E(	255	262	+	12.5202	3.26E-05	1
MA1020.1	Phatr3_J14849	Phatr3_E(	1303	1310	+	12.5202	3.26E-05	1
MA1020.1	Phatr3_J14849	Phatr3_J5	1548	1555	+	12.5202	3.26E-05	1
MA1020.1	Phatr3_J14849	Phatr3_J4	1721	1728	-	12.5202	3.26E-05	1
MA1020.1	Phatr3_J14849	Phatr3_E(	1128	1135	+	12.5202	3.26E-05	1
MA1020.1	Phatr3_J14849	Phatr3_J3	1570	1577	-	12.5202	3.26E-05	1
MA1020.1	Phatr3_J14849	Phatr3_J1	61	68	-	12.5202	3.26E-05	1
MA1020.1	Phatr3_J14849	Phatr3_J4	1762	1769	-	12.5202	3.26E-05	1
MA1020.1	Phatr3_J14849	Phatr3_J3	1619	1626	+	12.5202	3.26E-05	1
MA1020.1	Phatr3_J14849	Phatr3_J4	801	808	+	12.5202	3.26E-05	1
MA1020.1	Phatr3_J14849	Phatr3_J2	1769	1776	+	12.5202	3.26E-05	1
MA1020.1	Phatr3_J14849	Phatr3_J4	1828	1835	-	12.5202	3.26E-05	1
MA1020.1	Phatr3_J14849	Phatr3_J3	823	830	-	12.5202	3.26E-05	1
MA1020.1	Phatr3_J14849	Phatr3_J3	937	944	-	12.5202	3.26E-05	1
MA1020.1	Phatr3_J14849	Phatr3_J3	1017	1024	-	12.5202	3.26E-05	1
MA1020.1	Phatr3_J14849	Phatr3_J3	1164	1171	-	12.5202	3.26E-05	1
MA1020.1	Phatr3_J14849	Phatr3_J3	1012	1019	-	12.5202	3.26E-05	1
MA1020.1	Phatr3_J14849	Phatr3_J4	1992	1999	-	12.5202	3.26E-05	1
MA1020.1	Phatr3_J14849	Phatr3_J4	1841	1848	-	12.5202	3.26E-05	1
MA1020.1	Phatr3_J14849	Phatr3_J4	46	53	+	12.5202	3.26E-05	1
MA1020.1	Phatr3_J14849	Phatr3_J4	1672	1679	+	12.5202	3.26E-05	1
MA1020.1	Phatr3_J14849	Phatr3_E(	132	139	+	12.5202	3.26E-05	1
MA1020.1	Phatr3_J14849	Phatr3_J4	364	371	-	12.5202	3.26E-05	1
MA1020.1	Phatr3_J14849	Phatr3_E(	1035	1042	-	12.5202	3.26E-05	1

MA1020.1	Phatr3_J14849	Phatr3_J3	1035	1042	-	12.5202	3.26E-05	1
MA1020.1	Phatr3_J14849	Phatr3_J1	1658	1665	+	12.5202	3.26E-05	1
MA1020.1	Phatr3_J14849	Phatr3_EG	264	271	-	12.5202	3.26E-05	1
MA1020.1	Phatr3_J14849	Phatr3_J3	1685	1692	+	12.5202	3.26E-05	1
MA1020.1	Phatr3_J14849	Phatr3_J4	1730	1737	-	12.5202	3.26E-05	1
MA1020.1	Phatr3_J14849	Phatr3_J4	462	469	-	12.5202	3.26E-05	1
MA1020.1	Phatr3_J14849	Phatr3_EG	400	407	+	12.5202	3.26E-05	1
MA1020.1	Phatr3_J14849	Phatr3_EG	1970	1977	-	12.5202	3.26E-05	1
MA1020.1	Phatr3_J14849	Phatr3_EG	1141	1148	-	12.5202	3.26E-05	1
MA1020.1	Phatr3_J14849	Phatr3_J2	1772	1779	-	12.5202	3.26E-05	1
MA1020.1	Phatr3_J14849	Phatr3_J4	1767	1774	+	12.5202	3.26E-05	1
MA1020.1	Phatr3_J14849	Phatr3_J1	1796	1803	+	12.5202	3.26E-05	1
MA1020.1	Phatr3_J14849	Phatr3_J3	803	810	+	12.5202	3.26E-05	1
MA1020.1	Phatr3_J14849	Phatr3_J4	979	986	-	12.5202	3.26E-05	1
MA1020.1	Phatr3_J14849	Phatr3_J4	981	988	+	12.5202	3.26E-05	1
MA1020.1	Phatr3_J14849	Phatr3_EG	431	438	+	12.5202	3.26E-05	1
MA1020.1	Phatr3_J14849	Phatr3_J4	1259	1266	-	12.5202	3.26E-05	1
MA1020.1	Phatr3_J14849	Phatr3_J1	132	139	-	12.5202	3.26E-05	1
MA1020.1	Phatr3_J14849	Phatr3_J4	1404	1411	+	12.5202	3.26E-05	1
MA1020.1	Phatr3_J14849	Phatr3_J3	1368	1375	-	12.5202	3.26E-05	1
MA1020.1	Phatr3_J14849	Phatr3_J1	481	488	-	12.5202	3.26E-05	1
MA1020.1	Phatr3_J14849	Phatr3_J4	1406	1413	+	12.5202	3.26E-05	1
MA1020.1	Phatr3_J14849	Phatr3_EG	1379	1386	-	12.5202	3.26E-05	1
MA1020.1	Phatr3_J14849	Phatr3_J4	676	683	-	12.5202	3.26E-05	1
MA1020.1	Phatr3_J14849	Phatr3_EG	747	754	-	12.5202	3.26E-05	1
MA1020.1	Phatr3_J14849	Phatr3_J3	377	384	+	12.5202	3.26E-05	1
MA1020.1	Phatr3_J14849	Phatr3_J4	842	849	+	12.5202	3.26E-05	1
MA1020.1	Phatr3_J14849	Phatr3_EG	242	249	-	12.5202	3.26E-05	1
MA1020.1	Phatr3_J14849	Phatr3_EG	330	337	+	12.5202	3.26E-05	1
MA1020.1	Phatr3_J14849	EPrPhatr3	884	891	+	12.5202	3.26E-05	1
MA1020.1	Phatr3_J14849	Phatr3_EG	1114	1121	+	12.0573	8.69E-05	1
MA1020.1	Phatr3_J14849	Phatr3_J3	105	112	-	12.0573	8.69E-05	1
MA1020.1	Phatr3_J14849	Phatr3_J4	1912	1919	-	12.0573	8.69E-05	1
MA1020.1	Phatr3_J14849	Phatr3_J5	511	518	-	12.0573	8.69E-05	1
MA1020.1	Phatr3_J14849	Phatr3_J3	327	334	-	12.0573	8.69E-05	1
MA1020.1	Phatr3_J14849	Phatr3_J4	137	144	+	12.0573	8.69E-05	1
MA1020.1	Phatr3_J14849	Phatr3_J3	658	665	-	12.0573	8.69E-05	1
MA1020.1	Phatr3_J14849	Phatr3_J2	658	665	+	12.0573	8.69E-05	1
MA1020.1	Phatr3_J14849	Phatr3_J4	159	166	-	12.0573	8.69E-05	1
MA1020.1	Phatr3_J14849	Phatr3_J4	387	394	+	12.0573	8.69E-05	1
MA1020.1	Phatr3_J14849	Phatr3_J9	85	92	-	12.0573	8.69E-05	1
MA1020.1	Phatr3_J14849	Phatr3_J9	304	311	-	12.0573	8.69E-05	1
MA1020.1	Phatr3_J14849	Phatr3_J4	1016	1023	+	12.0573	8.69E-05	1
MA1020.1	Phatr3_J14849	Phatr3_J4	1235	1242	+	12.0573	8.69E-05	1
MA1020.1	Phatr3_J14849	Phatr3_J9	420	427	+	12.0573	8.69E-05	1
MA1020.1	Phatr3_J14849	Phatr3_J3	148	155	-	12.0573	8.69E-05	1
MA1020.1	Phatr3_J14849	Phatr3_J4	1763	1770	+	12.0573	8.69E-05	1
MA1020.1	Phatr3_J14849	Phatr3_J4	1545	1552	-	12.0573	8.69E-05	1
MA1020.1	Phatr3_J14849	Phatr3_J4	630	637	+	12.0573	8.69E-05	1
MA1020.1	Phatr3_J14849	Phatr3_EG	597	604	+	12.0573	8.69E-05	1
MA1020.1	Phatr3_J14849	Phatr3_EG	1857	1864	+	12.0573	8.69E-05	1
MA1020.1	Phatr3_J14849	Phatr3_J1	756	763	-	12.0573	8.69E-05	1
MA1020.1	Phatr3_J14849	Phatr3_EG	325	332	-	12.0573	8.69E-05	1
MA1020.1	Phatr3_J14849	Phatr3_J8	1089	1096	-	12.0573	8.69E-05	1

MA1020.1	Phatr3_J14849	Phatr3_J3	1081	1088	-	12.0573	8.69E-05	1
MA1020.1	Phatr3_J14849	Phatr3_J3	1329	1336	-	12.0573	8.69E-05	1
MA1020.1	Phatr3_J14849	Phatr3_EC	1430	1437	+	12.0573	8.69E-05	1
MA1020.1	Phatr3_J14849	Phatr3_EC	1678	1685	+	12.0573	8.69E-05	1
MA1020.1	Phatr3_J14849	Phatr3_EC	510	517	+	12.0573	8.69E-05	1
MA1020.1	Phatr3_J14849	Phatr3_EC	1009	1016	+	12.0573	8.69E-05	1
MA1020.1	Phatr3_J14849	Phatr3_J4	1745	1752	-	12.0573	8.69E-05	1
MA1020.1	Phatr3_J14849	Phatr3_J4	1287	1294	-	12.0573	8.69E-05	1
MA1020.1	Phatr3_J14849	Phatr3_EC	319	326	-	12.0573	8.69E-05	1
MA1020.1	Phatr3_J14849	Phatr3_J4	1695	1702	+	12.0573	8.69E-05	1
MA1020.1	Phatr3_J14849	Phatr3_J8	581	588	-	12.0573	8.69E-05	1
MA1020.1	Phatr3_J14849	Phatr3_J4	928	935	-	12.0573	8.69E-05	1
MA1020.1	Phatr3_J14849	Phatr3_EC	1908	1915	-	12.0573	8.69E-05	1
MA1020.1	Phatr3_J14849	Phatr3_EC	591	598	-	12.0573	8.69E-05	1
MA1020.1	Phatr3_J14849	Phatr3_J3	1089	1096	-	12.0573	8.69E-05	1
MA1020.1	Phatr3_J14849	Phatr3_J1	999	1006	-	12.0573	8.69E-05	1
MA1020.1	Phatr3_J14849	Phatr3_EC	120	127	+	12.0573	8.69E-05	1
MA1020.1	Phatr3_J14849	Phatr3_J3	1766	1773	-	12.0573	8.69E-05	1
MA1020.1	Phatr3_J14849	Phatr3_EC	1471	1478	+	12.0573	8.69E-05	1
MA1020.1	Phatr3_J14849	Phatr3_J1	1104	1111	+	12.0573	8.69E-05	1
MA1020.1	Phatr3_J14849	Phatr3_EC	138	145	+	12.0573	8.69E-05	1
MA1020.1	Phatr3_J14849	Phatr3_J1	1239	1246	-	12.0573	8.69E-05	1
MA1020.1	Phatr3_J14849	Phatr3_J4	71	78	-	12.0573	8.69E-05	1
MA1020.1	Phatr3_J14849	Phatr3_J4	1511	1518	-	12.0573	8.69E-05	1
MA1020.1	Phatr3_J14849	Phatr3_J9	458	465	+	12.0573	8.69E-05	1
MA1020.1	Phatr3_J14849	Phatr3_J4	1252	1259	-	12.0573	8.69E-05	1
MA1020.1	Phatr3_J14849	Phatr3_J4	934	941	-	12.0573	8.69E-05	1
MA1020.1	Phatr3_J14849	Phatr3_J3	1888	1895	+	12.0573	8.69E-05	1
MA1020.1	Phatr3_J14849	Phatr3_J4	1727	1734	-	12.0573	8.69E-05	1
MA1020.1	Phatr3_J14849	Phatr3_J3	976	983	+	12.0573	8.69E-05	1
MA1020.1	Phatr3_J14849	Phatr3_J4	996	1003	+	12.0573	8.69E-05	1
MA1020.1	Phatr3_J14849	Phatr3_J4	594	601	+	12.0573	8.69E-05	1
MA1020.1	Phatr3_J14849	Phatr3_J4	932	939	-	12.0573	8.69E-05	1
MA1020.1	Phatr3_J14849	Phatr3_J3	1212	1219	+	12.0573	8.69E-05	1
MA1020.1	Phatr3_J14849	Phatr3_J8	1500	1507	+	12.0573	8.69E-05	1
MA1020.1	Phatr3_J14849	Phatr3_J4	565	572	-	12.0573	8.69E-05	1
MA1020.1	Phatr3_J14849	Phatr3_J9	1333	1340	+	12.0573	8.69E-05	1
MA1020.1	Phatr3_J14849	Phatr3_J4	1073	1080	-	12.0573	8.69E-05	1
MA1020.1	Phatr3_J14849	Phatr3_EC	3	10	+	12.0573	8.69E-05	1
MA1020.1	Phatr3_J14849	Phatr3_J9	1418	1425	+	12.0573	8.69E-05	1
MA1020.1	Phatr3_J14849	Phatr3_J5	935	942	+	12.0573	8.69E-05	1
MA1020.1	Phatr3_J14849	Phatr3_J1	1323	1330	+	12.0573	8.69E-05	1
MA1020.1	Phatr3_J14849	PTsRNA00C	1950	1957	+	12.0573	8.69E-05	1
MA1020.1	Phatr3_J14849	Phatr3_J4	205	212	-	12.0573	8.69E-05	1
MA1020.1	Phatr3_J14849	Phatr3_EC	1968	1975	+	12.0573	8.69E-05	1
MA1020.1	Phatr3_J14849	Phatr3_J4	346	353	-	12.0573	8.69E-05	1
MA1020.1	Phatr3_J14849	Phatr3_J3	1394	1401	-	12.0573	8.69E-05	1
MA1020.1	Phatr3_J14849	Phatr3_J1	1108	1115	-	12.0573	8.69E-05	1
MA1020.1	Phatr3_J14849	Phatr3_J4	1759	1766	+	12.0573	8.69E-05	1
MA1020.1	Phatr3_J14849	Phatr3_J4	1720	1727	-	12.0573	8.69E-05	1
MA1020.1	Phatr3_J14849	Phatr3_J4	1780	1787	+	12.0573	8.69E-05	1
MA1020.1	Phatr3_J14849	Phatr3_J1	1455	1462	-	12.0573	8.69E-05	1
MA1020.1	Phatr3_J14849	Phatr3_EC	630	637	+	12.0573	8.69E-05	1
MA1020.1	Phatr3_J14849	Phatr3_EC	724	731	+	12.0573	8.69E-05	1

MA1020.1	Phatr3_J14849	Phatr3_J3	738	745	-	12.0573	8.69E-05	1
MA1020.1	Phatr3_J14849	Phatr3_J1	479	486	+	12.0573	8.69E-05	1
MA1020.1	Phatr3_J14849	Phatr3_EC	1446	1453	-	12.0573	8.69E-05	1
MA1020.1	Phatr3_J14849	Phatr3_EC	523	530	-	12.0573	8.69E-05	1
MA1020.1	Phatr3_J14849	Phatr3_EC	1747	1754	-	12.0573	8.69E-05	1
MA1020.1	Phatr3_J14849	Phatr3_EC	1651	1658	-	12.0573	8.69E-05	1
MA1020.1	Phatr3_J14849	Phatr3_J4	686	693	-	12.0573	8.69E-05	1
MA1020.1	Phatr3_J14849	Phatr3_J4	423	430	-	12.0573	8.69E-05	1
MA1020.1	Phatr3_J14849	Phatr3_J4	1821	1828	+	12.0573	8.69E-05	1
MA1020.1	Phatr3_J14849	Phatr3_J4	511	518	-	12.0573	8.69E-05	1
MA1020.1	Phatr3_J14849	Phatr3_J4	529	536	-	12.0573	8.69E-05	1
MA1020.1	Phatr3_J14849	Phatr3_J1	453	460	-	12.0573	8.69E-05	1
MA1020.1	Phatr3_J14849	Phatr3_J1	742	749	+	12.0573	8.69E-05	1
MA1020.1	Phatr3_J14849	Phatr3_EC	1167	1174	-	12.0573	8.69E-05	1
MA1020.1	Phatr3_J14849	Phatr3_EC	1456	1463	+	12.0573	8.69E-05	1
MA1020.1	Phatr3_J14849	Phatr3_J3	1264	1271	+	12.0573	8.69E-05	1
MA1020.1	Phatr3_J14849	Phatr3_J3	1512	1519	+	12.0573	8.69E-05	1
MA1020.1	Phatr3_J14849	Phatr3_EC	954	961	+	12.0573	8.69E-05	1
MA1020.1	Phatr3_J14849	Phatr3_EC	1202	1209	+	12.0573	8.69E-05	1
MA1020.1	Phatr3_J14849	Phatr3_J3	897	904	-	12.0573	8.69E-05	1
MA1020.1	Phatr3_J14849	Phatr3_EC	1667	1674	-	12.0573	8.69E-05	1
MA1020.1	Phatr3_J14849	Phatr3_EC	1391	1398	-	12.0573	8.69E-05	1
MA1020.1	Phatr3_J14849	Phatr3_EC	902	909	-	12.0573	8.69E-05	1
MA1020.1	Phatr3_J14849	Phatr3_J4	1697	1704	-	12.0573	8.69E-05	1
MA1020.1	Phatr3_J14849	Phatr3_J1	1632	1639	+	12.0573	8.69E-05	1
MA1020.1	Phatr3_J14849	Phatr3_J3	334	341	+	12.0573	8.69E-05	1
MA1020.1	Phatr3_J14849	Phatr3_J3	1205	1212	+	12.0573	8.69E-05	1
MA1020.1	Phatr3_J14849	Phatr3_J1	371	378	-	12.0573	8.69E-05	1
MA1020.1	Phatr3_J14849	Phatr3_J5	666	673	+	12.0573	8.69E-05	1
MA1020.1	Phatr3_J14849	Phatr3_J4	1950	1957	+	12.0573	8.69E-05	1
MA1020.1	Phatr3_J14849	Phatr3_EC	451	458	-	12.0573	8.69E-05	1
MA1020.1	Phatr3_J14849	Phatr3_J4	682	689	+	12.0573	8.69E-05	1
MA1020.1	Phatr3_J14849	Phatr3_J4	1474	1481	-	12.0573	8.69E-05	1
MA1020.1	Phatr3_J14849	Phatr3_J4	823	830	+	12.0573	8.69E-05	1
MA1020.1	Phatr3_J14849	Phatr3_J4	1615	1622	-	12.0573	8.69E-05	1
MA1020.1	Phatr3_J14849	Phatr3_EC	1569	1576	+	12.0573	8.69E-05	1
MA1020.1	Phatr3_J14849	Phatr3_J3	257	264	+	12.0573	8.69E-05	1
MA1020.1	Phatr3_J14849	Phatr3_J4	1162	1169	-	12.0573	8.69E-05	1
MA1020.1	Phatr3_J14849	Phatr3_J4	1253	1260	+	12.0573	8.69E-05	1
MA1020.1	Phatr3_J14849	Phatr3_J4	380	387	+	12.0573	8.69E-05	1
MA1020.1	Phatr3_J14849	Phatr3_EC	777	784	+	12.0573	8.69E-05	1
MA1020.1	Phatr3_J14849	Phatr3_EC	1010	1017	+	12.0573	8.69E-05	1
MA1020.1	Phatr3_J14849	Phatr3_J1	948	955	+	12.0573	8.69E-05	1
MA1020.1	Phatr3_J14849	Phatr3_J1	1178	1185	+	12.0573	8.69E-05	1
MA1020.1	Phatr3_J14849	Phatr3_J4	274	281	+	12.0573	8.69E-05	1
MA1020.1	Phatr3_J14849	Phatr3_J3	208	215	-	12.0573	8.69E-05	1
MA1020.1	Phatr3_J14849	Phatr3_EC	888	895	-	12.0573	8.69E-05	1
MA1020.1	Phatr3_J14849	Phatr3_J4	1259	1266	+	12.0573	8.69E-05	1
MA1020.1	Phatr3_J14849	Phatr3_EC	1572	1579	+	12.0573	8.69E-05	1
MA1020.1	Phatr3_J14849	Phatr3_EC	58	65	+	12.0573	8.69E-05	1
MA1020.1	Phatr3_J14849	Phatr3_EC	303	310	+	12.0573	8.69E-05	1
MA1020.1	Phatr3_J14849	Phatr3_EC	757	764	+	12.0573	8.69E-05	1
MA1020.1	Phatr3_J14849	Phatr3_EC	1002	1009	+	12.0573	8.69E-05	1
MA1020.1	Phatr3_J14849	Phatr3_EC	1291	1298	+	12.0573	8.69E-05	1

MA1020.1	Phatr3_J14849	Phatr3_EC	1536	1543	+	12.0573	8.69E-05	1
MA1020.1	Phatr3_J14849	Phatr3_J4	703	710	-	12.0573	8.69E-05	1
MA1020.1	Phatr3_J14849	Phatr3_J2	1628	1635	-	12.0573	8.69E-05	1
MA1020.1	Phatr3_J14849	Phatr3_J4	1726	1733	+	12.0573	8.69E-05	1
MA1020.1	Phatr3_J14849	Phatr3_EC	1076	1083	+	12.0573	8.69E-05	1
MA1020.1	Phatr3_J14849	EPrPhatr3	918	925	+	12.0573	8.69E-05	1
MA1020.1	Phatr3_J14849	Phatr3_J4	1367	1374	+	12.0573	8.69E-05	1
MA1020.1	Phatr3_J14849	Phatr3_J4	1615	1622	+	12.0573	8.69E-05	1
MA1020.1	Phatr3_J14849	Phatr3_EC	1081	1088	-	12.0573	8.69E-05	1
MA1020.1	Phatr3_J14849	Phatr3_EC	1329	1336	-	12.0573	8.69E-05	1
MA1020.1	Phatr3_J14849	Phatr3_EC	1676	1683	-	12.0573	8.69E-05	1
MA1020.1	Phatr3_J14849	Phatr3_EC	1924	1931	-	12.0573	8.69E-05	1
MA1020.1	Phatr3_J14849	Phatr3_J4	222	229	+	12.0573	8.69E-05	1
MA1020.1	Phatr3_J14849	Phatr3_EC	790	797	-	12.0573	8.69E-05	1
MA1020.1	Phatr3_J14849	Phatr3_J4	1020	1027	+	12.0573	8.69E-05	1
MA1020.1	Phatr3_J14849	Phatr3_EC	648	655	+	12.0573	8.69E-05	1
MA1020.1	Phatr3_J14849	Phatr3_J4	1567	1574	-	12.0573	8.69E-05	1
MA1020.1	Phatr3_J14849	Phatr3_J4	515	522	-	12.0573	8.69E-05	1
MA1020.1	Phatr3_J14849	Phatr3_J3	668	675	-	12.0573	8.69E-05	1
MA1020.1	Phatr3_J14849	Phatr3_J4	834	841	-	12.0573	8.69E-05	1
MA1020.1	Phatr3_J14849	Phatr3_J3	1461	1468	+	12.0573	8.69E-05	1
MA1020.1	Phatr3_J14849	Phatr3_J4	104	111	+	12.0573	8.69E-05	1
MA1020.1	Phatr3_J14849	Phatr3_EC	1747	1754	-	12.0573	8.69E-05	1
MA1020.1	Phatr3_J14849	Phatr3_J3	1213	1220	+	12.0573	8.69E-05	1
MA1020.1	Phatr3_J14849	Phatr3_J6	1112	1119	+	12.0573	8.69E-05	1
MA1020.1	Phatr3_J14849	Phatr3_J4	473	480	+	12.0573	8.69E-05	1
MA1020.1	Phatr3_J14849	Phatr3_J4	885	892	-	12.0573	8.69E-05	1
MA1020.1	Phatr3_J14849	Phatr3_J1	308	315	+	12.0573	8.69E-05	1
MA1020.1	Phatr3_J14849	Phatr3_J3	1971	1978	+	12.0573	8.69E-05	1
MA1020.1	Phatr3_J14849	Phatr3_J4	473	480	+	12.0573	8.69E-05	1
MA1020.1	Phatr3_J14849	Phatr3_J4	1707	1714	-	12.0573	8.69E-05	1
MA1020.1	Phatr3_J14849	Phatr3_J1	1639	1646	-	12.0573	8.69E-05	1
MA1020.1	Phatr3_J14849	Phatr3_EC	1023	1030	-	12.0573	8.69E-05	1
MA1020.1	Phatr3_J14849	Phatr3_J4	1054	1061	+	12.0573	8.69E-05	1
MA1020.1	Phatr3_J14849	Phatr3_J4	730	737	+	12.0573	8.69E-05	1
MA1020.1	Phatr3_J14849	Phatr3_J2	995	1002	-	12.0573	8.69E-05	1
MA1020.1	Phatr3_J14849	Phatr3_J4	741	748	+	12.0573	8.69E-05	1
MA1020.1	Phatr3_J14849	Phatr3_J5	240	247	-	12.0573	8.69E-05	1
MA1020.1	Phatr3_J14849	Phatr3_J1	1394	1401	-	12.0573	8.69E-05	1
MA1020.1	Phatr3_J14849	Phatr3_J4	570	577	+	12.0573	8.69E-05	1
MA1020.1	Phatr3_J14849	Phatr3_J4	1899	1906	-	12.0573	8.69E-05	1
MA1020.1	Phatr3_J14849	Phatr3_J4	1716	1723	+	12.0573	8.69E-05	1
MA1020.1	Phatr3_J14849	Phatr3_J4	1828	1835	+	12.0573	8.69E-05	1
MA1020.1	Phatr3_J14849	Phatr3_J1	1245	1252	-	12.0573	8.69E-05	1
MA1020.1	Phatr3_J14849	Phatr3_J4	229	236	+	12.0573	8.69E-05	1
MA1020.1	Phatr3_J14849	Phatr3_EC	498	505	-	12.0573	8.69E-05	1
MA1020.1	Phatr3_J14849	Phatr3_J4	1410	1417	+	12.0573	8.69E-05	1
MA1020.1	Phatr3_J14849	Phatr3_EC	1312	1319	-	12.0573	8.69E-05	1
MA1020.1	Phatr3_J14849	Phatr3_J4	462	469	+	12.0573	8.69E-05	1
MA1020.1	Phatr3_J14849	Phatr3_J4	533	540	-	12.0573	8.69E-05	1
MA1020.1	Phatr3_J14849	Phatr3_J4	1779	1786	-	12.0573	8.69E-05	1
MA1020.1	Phatr3_J14849	Phatr3_J4	121	128	-	12.0573	8.69E-05	1
MA1020.1	Phatr3_J14849	Phatr3_EC	589	596	+	12.0573	8.69E-05	1
MA1020.1	Phatr3_J14849	Phatr3_J4	1136	1143	+	12.0573	8.69E-05	1

MA1020.1	Phatr3_J14849	Phatr3_J3	1416	1423	+	12.0573	8.69E-05	1
MA1020.1	Phatr3_J14849	Phatr3_J4	1465	1472	-	12.0573	8.69E-05	1
MA1020.1	Phatr3_J14849	Phatr3_EC	76	83	+	12.0573	8.69E-05	1
MA1020.1	Phatr3_J14849	Phatr3_EC	1502	1509	-	12.0573	8.69E-05	1
MA1020.1	Phatr3_J14849	Phatr3_EC	734	741	+	12.0573	8.69E-05	1
MA1020.1	Phatr3_J14849	Phatr3_EC	1435	1442	+	12.0573	8.69E-05	1
MA1020.1	Phatr3_J14849	Phatr3_J4	140	147	+	12.0573	8.69E-05	1
MA1020.1	Phatr3_J14849	Phatr3_EC	236	243	+	12.0573	8.69E-05	1
MA1020.1	Phatr3_J14849	Phatr3_J4	164	171	+	12.0573	8.69E-05	1
MA1020.1	Phatr3_J14849	Phatr3_J4	1347	1354	-	12.0573	8.69E-05	1
MA1020.1	Phatr3_J14849	Phatr3_J4	1984	1991	+	12.0573	8.69E-05	1
MA1020.1	Phatr3_J14849	Phatr3_J2	285	292	-	12.0573	8.69E-05	1
MA1020.1	Phatr3_J14849	Phatr3_J2	922	929	+	12.0573	8.69E-05	1
MA1020.1	Phatr3_J14849	Phatr3_J3	1472	1479	-	12.0573	8.69E-05	1
MA1020.1	Phatr3_J14849	Phatr3_J5	285	292	+	12.0573	8.69E-05	1
MA1020.1	Phatr3_J14849	Phatr3_EC	787	794	+	12.0573	8.69E-05	1
MA1020.1	Phatr3_J14849	Phatr3_J3	206	213	-	12.0573	8.69E-05	1
MA1020.1	Phatr3_J14849	Phatr3_J2	1779	1786	+	12.0573	8.69E-05	1
MA1020.1	Phatr3_J14849	Phatr3_J1	1740	1747	-	12.0573	8.69E-05	1
MA1020.1	Phatr3_J14849	Phatr3_EC	1527	1534	+	12.0573	8.69E-05	1
MA1020.1	Phatr3_J14849	Phatr3_J4	159	166	-	12.0573	8.69E-05	1
MA1020.1	Phatr3_J14849	Phatr3_EC	482	489	+	12.0573	8.69E-05	1
MA1020.1	Phatr3_J14849	Phatr3_J2	467	474	+	12.0573	8.69E-05	1
MA1020.1	Phatr3_J14849	Phatr3_EC	324	331	-	12.0573	8.69E-05	1
MA1020.1	Phatr3_J14849	Phatr3_EC	572	579	-	12.0573	8.69E-05	1
MA1020.1	Phatr3_J14849	Phatr3_J4	1213	1220	+	12.0573	8.69E-05	1
MA1020.1	Phatr3_J14849	Phatr3_J4	1461	1468	+	12.0573	8.69E-05	1
MA1020.1	Phatr3_J14849	Phatr3_J3	1240	1247	-	12.0573	8.69E-05	1
MA1020.1	Phatr3_J14849	Phatr3_J3	1488	1495	-	12.0573	8.69E-05	1
MA1020.1	Phatr3_J14849	Phatr3_J4	610	617	+	12.0573	8.69E-05	1
MA1020.1	Phatr3_J14849	Phatr3_EC	307	314	-	12.0573	8.69E-05	1
MA1020.1	Phatr3_J14849	Phatr3_J3	1355	1362	+	12.0573	8.69E-05	1
MA1020.1	Phatr3_J14849	Phatr3_J5	1077	1084	-	12.0573	8.69E-05	1
MA1020.1	Phatr3_J14849	Phatr3_J4	547	554	-	12.0573	8.69E-05	1
MA1020.1	Phatr3_J14849	Phatr3_J5	1507	1514	+	12.0573	8.69E-05	1
MA1020.1	Phatr3_J14849	Phatr3_J5	533	540	-	12.0573	8.69E-05	1
MA1020.1	Phatr3_J14849	Phatr3_J3	1273	1280	+	12.0573	8.69E-05	1
MA1020.1	Phatr3_J14849	Phatr3_J4	1047	1054	-	12.0573	8.69E-05	1
MA1020.1	Phatr3_J14849	Phatr3_EC	202	209	-	12.0573	8.69E-05	1
MA1020.1	Phatr3_J14849	Phatr3_J3	1948	1955	-	12.0573	8.69E-05	1
MA1020.1	Phatr3_J14849	Phatr3_EC	1604	1611	+	12.0573	8.69E-05	1
MA1020.1	Phatr3_J14849	Phatr3_J4	1641	1648	+	12.0573	8.69E-05	1
MA1020.1	Phatr3_J14849	Phatr3_EC	1720	1727	+	12.0573	8.69E-05	1
MA1020.1	Phatr3_J14849	Phatr3_J4	346	353	+	12.0573	8.69E-05	1
MA1020.1	Phatr3_J14849	Phatr3_J2	1775	1782	-	12.0573	8.69E-05	1
MA1020.1	Phatr3_J14849	Phatr3_J4	1838	1845	+	12.0573	8.69E-05	1
MA1020.1	Phatr3_J14849	Phatr3_J2	772	779	+	12.0573	8.69E-05	1
MA1020.1	Phatr3_J14849	Phatr3_J4	1952	1959	+	12.0573	8.69E-05	1
MA1020.1	Phatr3_J14849	Phatr3_J4	384	391	-	12.0573	8.69E-05	1
MA1020.1	Phatr3_J14849	Phatr3_J4	1778	1785	-	12.0573	8.69E-05	1
MA1020.1	Phatr3_J14849	Phatr3_J1	1807	1814	+	12.0573	8.69E-05	1
MA1020.1	Phatr3_J14849	Phatr3_J1	1821	1828	-	12.0573	8.69E-05	1
MA1020.1	Phatr3_J14849	Phatr3_J4	1194	1201	-	12.0573	8.69E-05	1
MA1020.1	Phatr3_J14849	Phatr3_EC	979	986	-	12.0573	8.69E-05	1

MA1020.1	Phatr3_J14849	Phatr3_E(	1747	1754	-	12.0573	8.69E-05	1
MA1020.1	Phatr3_J14849	Phatr3_J3	799	806	+	12.0573	8.69E-05	1
MA1020.1	Phatr3_J14849	Phatr3_J3	1991	1998	+	12.0573	8.69E-05	1
MA1020.1	Phatr3_J14849	Phatr3_J4	1724	1731	-	12.0573	8.69E-05	1
MA1020.1	Phatr3_J14849	Phatr3_J4	965	972	-	12.0573	8.69E-05	1
MA1020.1	Phatr3_J14849	Phatr3_E(	615	622	-	12.0573	8.69E-05	1
MA1020.1	Phatr3_J14849	Phatr3_J4	719	726	+	12.0573	8.69E-05	1
MA1020.1	Phatr3_J14849	Phatr3_E(	475	482	-	12.0573	8.69E-05	1
MA1020.1	Phatr3_J14849	Phatr3_J2	773	780	+	12.0573	8.69E-05	1
MA1020.1	Phatr3_J14849	Phatr3_J4	1510	1517	+	12.0573	8.69E-05	1
MA1020.1	Phatr3_J14849	Phatr3_J3	1464	1471	-	12.0573	8.69E-05	1
MA1020.1	Phatr3_J14849	Phatr3_J4	909	916	+	12.0573	8.69E-05	1
MA1020.1	Phatr3_J14849	Phatr3_J4	57	64	-	12.0573	8.69E-05	1
MA1020.1	Phatr3_J14849	Phatr3_E(	15	22	+	12.0573	8.69E-05	1
MA1020.1	Phatr3_J14849	Phatr3_E(	140	147	+	12.0573	8.69E-05	1
MA1020.1	Phatr3_J14849	Phatr3_E(	236	243	-	12.0573	8.69E-05	1
MA1020.1	Phatr3_J14849	Phatr3_E(	883	890	+	12.0573	8.69E-05	1
MA1020.1	Phatr3_J14849	Phatr3_E(	979	986	-	12.0573	8.69E-05	1
MA1020.1	Phatr3_J14849	Phatr3_E(	1651	1658	+	12.0573	8.69E-05	1
MA1020.1	Phatr3_J14849	Phatr3_E(	1747	1754	-	12.0573	8.69E-05	1
MA1020.1	Phatr3_J14849	Phatr3_E(	1956	1963	-	12.0573	8.69E-05	1
MA1020.1	Phatr3_J14849	Phatr3_J4	166	173	-	12.0573	8.69E-05	1
MA1020.1	Phatr3_J14849	Phatr3_J4	1517	1524	-	12.0573	8.69E-05	1
MA1020.1	Phatr3_J14849	Phatr3_J4	1650	1657	+	12.0573	8.69E-05	1
MA1020.1	Phatr3_J14849	Phatr3_J4	1115	1122	-	12.0573	8.69E-05	1
MA1020.1	Phatr3_J14849	Phatr3_E(	325	332	-	12.0573	8.69E-05	1
MA1020.1	Phatr3_J14849	Phatr3_E(	941	948	-	12.0573	8.69E-05	1
MA1020.1	Phatr3_J14849	Phatr3_J4	11	18	-	12.0573	8.69E-05	1
MA1020.1	Phatr3_J14849	Phatr3_J3	1830	1837	-	12.0573	8.69E-05	1
MA1020.1	Phatr3_J14849	Phatr3_J4	1796	1803	+	12.0573	8.69E-05	1
MA1020.1	Phatr3_J14849	Phatr3_E(	453	460	-	12.0573	8.69E-05	1
MA1020.1	Phatr3_J14849	Phatr3_E(	1408	1415	+	12.0573	8.69E-05	1
MA1020.1	Phatr3_J14849	Phatr3_J4	1654	1661	+	12.0573	8.69E-05	1
MA1020.1	Phatr3_J14849	Phatr3_J1	601	608	-	12.0573	8.69E-05	1
MA1020.1	Phatr3_J14849	Phatr3_J1	1123	1130	+	12.0573	8.69E-05	1
MA1020.1	Phatr3_J14849	Phatr3_J3	1351	1358	+	12.0573	8.69E-05	1
MA1020.1	Phatr3_J14849	Phatr3_J5	288	295	-	12.0573	8.69E-05	1
MA1020.1	Phatr3_J14849	Phatr3_E(	781	788	-	12.0573	8.69E-05	1
MA1020.1	Phatr3_J14849	Phatr3_J4	1859	1866	-	12.0573	8.69E-05	1
MA1020.1	Phatr3_J14849	Phatr3_J3	1592	1599	+	12.0573	8.69E-05	1
MA1020.1	Phatr3_J14849	Phatr3_J4	1795	1802	+	12.0573	8.69E-05	1
MA1020.1	Phatr3_J14849	Phatr3_J4	1553	1560	-	12.0573	8.69E-05	1
MA1020.1	Phatr3_J14849	Phatr3_J4	36	43	-	12.0573	8.69E-05	1
MA1020.1	Phatr3_J14849	Phatr3_E(	122	129	+	12.0573	8.69E-05	1
MA1020.1	Phatr3_J14849	Phatr3_J4	316	323	-	12.0573	8.69E-05	1
MA1020.1	Phatr3_J14849	Phatr3_J4	540	547	-	12.0573	8.69E-05	1
MA1020.1	Phatr3_J14849	Phatr3_E(	1258	1265	-	12.0573	8.69E-05	1
MA1020.1	Phatr3_J14849	Phatr3_J9	1614	1621	-	12.0573	8.69E-05	1
MA1020.1	Phatr3_J14849	Phatr3_J4	322	329	+	12.0573	8.69E-05	1
MA1020.1	Phatr3_J14849	Phatr3_E(	694	701	+	12.0573	8.69E-05	1
MA1020.1	Phatr3_J14849	Phatr3_J4	1605	1612	-	12.0573	8.69E-05	1
MA1020.1	Phatr3_J14849	Phatr3_J4	1849	1856	+	12.0573	8.69E-05	1
MA1020.1	Phatr3_J14849	Phatr3_J1	148	155	-	12.0573	8.69E-05	1
MA1020.1	Phatr3_J14849	Phatr3_J2	976	983	+	12.0573	8.69E-05	1

MA1020.1	Phatr3_J14849	Phatr3_J4	1109	1116	-	12.0573	8.69E-05	1
MA1020.1	Phatr3_J14849	Phatr3_J4	1156	1163	+	12.0573	8.69E-05	1
MA1020.1	Phatr3_J14849	Phatr3_J7	754	761	+	12.0573	8.69E-05	1
MA1020.1	Phatr3_J14849	Phatr3_J2	1222	1229	-	12.0573	8.69E-05	1
MA1020.1	Phatr3_J14849	Phatr3_J4	1669	1676	-	12.0573	8.69E-05	1
MA1020.1	Phatr3_J14849	PTsRNA000	1953	1960	-	12.0573	8.69E-05	1
MA1020.1	Phatr3_J14849	Phatr3_EC	1768	1775	-	12.0573	8.69E-05	1
MA1020.1	Phatr3_J14849	Phatr3_J3	398	405	+	12.0573	8.69E-05	1
MA1020.1	Phatr3_J14849	Phatr3_EC	1904	1911	-	12.0573	8.69E-05	1
MA1020.1	Phatr3_J14849	Phatr3_EC	332	339	-	12.0573	8.69E-05	1
MA1020.1	Phatr3_J14849	Phatr3_J4	1740	1747	+	12.0573	8.69E-05	1
MA1020.1	Phatr3_J14849	Phatr3_J5	147	154	-	12.0573	8.69E-05	1
MA1020.1	Phatr3_J14849	Phatr3_J1	955	962	+	12.0573	8.69E-05	1
MA1020.1	Phatr3_J14849	Phatr3_J1	671	678	-	12.0573	8.69E-05	1
MA1020.1	Phatr3_J14849	Phatr3_EC	1732	1739	+	12.0573	8.69E-05	1
MA1020.1	Phatr3_J14849	Phatr3_J1	1162	1169	-	12.0573	8.69E-05	1
MA1020.1	Phatr3_J14849	Phatr3_EC	1084	1091	+	12.0573	8.69E-05	1
MA1020.1	Phatr3_J14849	Phatr3_EC	1069	1076	-	12.0573	8.69E-05	1
MA1020.1	Phatr3_J14849	Phatr3_J4	698	705	+	12.0573	8.69E-05	1
MA1020.1	Phatr3_J14849	Phatr3_J5	654	661	+	12.0573	8.69E-05	1
MA1020.1	Phatr3_J14849	Phatr3_J4	752	759	-	12.0573	8.69E-05	1
MA1020.1	Phatr3_J14849	Phatr3_J4	1792	1799	-	12.0573	8.69E-05	1
MA1020.1	Phatr3_J14849	Phatr3_EC	127	134	+	12.0573	8.69E-05	1
MA1020.1	Phatr3_J14849	Phatr3_EC	920	927	+	12.0573	8.69E-05	1
MA1020.1	Phatr3_J14849	Phatr3_J1	1210	1217	-	12.0573	8.69E-05	1
MA1020.1	Phatr3_J14849	Phatr3_J4	1832	1839	+	12.0573	8.69E-05	1
MA1020.1	Phatr3_J14849	Phatr3_J4	295	302	+	12.0573	8.69E-05	1
MA1020.1	Phatr3_J14849	Phatr3_J4	1020	1027	-	12.0573	8.69E-05	1
MA1020.1	Phatr3_J14849	Phatr3_J4	1465	1472	+	12.0573	8.69E-05	1
MA1020.1	Phatr3_J14849	Phatr3_J4	220	227	+	12.0573	8.69E-05	1
MA1020.1	Phatr3_J14849	Phatr3_J4	201	208	+	12.0573	8.69E-05	1
MA1020.1	Phatr3_J14849	Phatr3_J3	251	258	-	12.0573	8.69E-05	1
MA1020.1	Phatr3_J14849	Phatr3_J4	334	341	+	12.0573	8.69E-05	1
MA1020.1	Phatr3_J14849	Phatr3_J3	1616	1623	-	12.0573	8.69E-05	1
MA1020.1	Phatr3_J14849	Phatr3_J4	34	41	-	12.0573	8.69E-05	1
MA1020.1	Phatr3_J14849	Phatr3_J4	1849	1856	-	12.0573	8.69E-05	1
MA1020.1	Phatr3_J14849	Phatr3_J4	1261	1268	+	12.0573	8.69E-05	1
MA1020.1	Phatr3_J14849	PTsRNA000	1411	1418	-	12.0573	8.69E-05	1
MA1020.1	Phatr3_J14849	Phatr3_EC	594	601	-	12.0573	8.69E-05	1
MA1020.1	Phatr3_J14849	Phatr3_J3	29	36	-	12.0573	8.69E-05	1
MA1020.1	Phatr3_J14849	Phatr3_J3	1943	1950	+	12.0573	8.69E-05	1
MA1020.1	Phatr3_J14849	Phatr3_J4	810	817	-	12.0573	8.69E-05	1
MA1020.1	Phatr3_J14849	Phatr3_J3	539	546	-	12.0573	8.69E-05	1
MA1020.1	Phatr3_J14849	Phatr3_J4	1706	1713	+	12.0573	8.69E-05	1
MA1020.1	Phatr3_J14849	Phatr3_EC	1198	1205	-	12.0573	8.69E-05	1
MA1020.1	Phatr3_J14849	Phatr3_J4	986	993	+	12.0573	8.69E-05	1
MA1020.1	Phatr3_J14849	Phatr3_J4	52	59	+	12.0573	8.69E-05	1
MA1020.1	Phatr3_J14849	Phatr3_J4	1205	1212	-	12.0573	8.69E-05	1
MA1020.1	Phatr3_J14849	Phatr3_J7	528	535	-	12.0573	8.69E-05	1
MA1020.1	Phatr3_J14849	Phatr3_J4	82	89	-	12.0573	8.69E-05	1
MA1020.1	Phatr3_J14849	Phatr3_J4	854	861	+	12.0573	8.69E-05	1
MA1020.1	Phatr3_J14849	Phatr3_J4	1036	1043	-	12.0573	8.69E-05	1
MA1020.1	Phatr3_J14849	Phatr3_J4	948	955	+	12.0573	8.69E-05	1
MA1020.1	Phatr3_J14849	Phatr3_J4	1241	1248	+	12.0573	8.69E-05	1

MA1020.1	Phatr3_J14849	Phatr3_EC	346	353	-	12.0573	8.69E-05	1
MA1020.1	Phatr3_J14849	Phatr3_J6	853	860	-	12.0573	8.69E-05	1
MA1020.1	Phatr3_J14849	Phatr3_J4	1357	1364	-	12.0573	8.69E-05	1
MA1020.1	Phatr3_J14849	Phatr3_J5	1523	1530	+	12.0573	8.69E-05	1
MA1020.1	Phatr3_J14849	Phatr3_EC	1631	1638	-	12.0573	8.69E-05	1
MA1020.1	Phatr3_J14849	Phatr3_EC	1118	1125	+	12.0573	8.69E-05	1
MA1020.1	Phatr3_J14849	Phatr3_J4	106	113	-	12.0573	8.69E-05	1
MA1020.1	Phatr3_J14849	Phatr3_J4	1613	1620	+	12.0573	8.69E-05	1
MA1020.1	Phatr3_J14849	Phatr3_J1	1820	1827	+	12.0573	8.69E-05	1
MA1020.1	Phatr3_J14849	Phatr3_J4	1313	1320	+	12.0573	8.69E-05	1
MA1020.1	Phatr3_J14849	Phatr3_J4	1927	1934	+	12.0573	8.69E-05	1
MA1020.1	Phatr3_J14849	Phatr3_J4	162	169	+	12.0573	8.69E-05	1
MA1020.1	Phatr3_J14849	Phatr3_J4	1165	1172	-	12.0573	8.69E-05	1
MA1020.1	Phatr3_J14849	Phatr3_EC	369	376	-	12.0573	8.69E-05	1
MA1020.1	Phatr3_J14849	Phatr3_EC	1109	1116	+	12.0573	8.69E-05	1
MA1020.1	Phatr3_J14849	Phatr3_J4	1475	1482	-	12.0573	8.69E-05	1
MA1020.1	Phatr3_J14849	Phatr3_EC	1973	1980	+	12.0573	8.69E-05	1
MA1020.1	Phatr3_J14849	Phatr3_EC	905	912	-	12.0573	8.69E-05	1
MA1020.1	Phatr3_J14849	Phatr3_J4	832	839	+	12.0573	8.69E-05	1
MA1020.1	Phatr3_J14849	PTsRNA00C	896	903	-	12.0573	8.69E-05	1
MA1020.1	Phatr3_J14849	Phatr3_J2	562	569	-	12.0573	8.69E-05	1
MA1020.1	Phatr3_J14849	Phatr3_J2	980	987	+	12.0573	8.69E-05	1
MA1020.1	Phatr3_J14849	Phatr3_J4	917	924	+	12.0573	8.69E-05	1
MA1020.1	Phatr3_J14849	Phatr3_EC	450	457	-	12.0573	8.69E-05	1
MA1020.1	Phatr3_J14849	Phatr3_EC	1229	1236	-	12.0573	8.69E-05	1
MA1020.1	Phatr3_J14849	Phatr3_EC	1741	1748	-	12.0573	8.69E-05	1
MA1020.1	Phatr3_J14849	Phatr3_J4	1701	1708	+	12.0573	8.69E-05	1
MA1020.1	Phatr3_J14849	Phatr3_J4	1792	1799	-	12.0573	8.69E-05	1
MA1020.1	Phatr3_J14849	Phatr3_J4	1271	1278	+	12.0573	8.69E-05	1
MA1020.1	Phatr3_J14849	Phatr3_J4	1114	1121	-	12.0573	8.69E-05	1
MA1020.1	Phatr3_J14849	Phatr3_J1	559	566	-	12.0573	8.69E-05	1
MA1020.1	Phatr3_J14849	Phatr3_J4	1406	1413	-	12.0573	8.69E-05	1
MA1020.1	Phatr3_J14849	Phatr3_J4	1827	1834	+	12.0573	8.69E-05	1
MA1020.1	Phatr3_J14849	Phatr3_EC	1748	1755	-	12.0573	8.69E-05	1
MA1020.1	Phatr3_J14849	Phatr3_EC	1175	1182	-	12.0573	8.69E-05	1
MA1020.1	Phatr3_J14849	Phatr3_J1	1853	1860	+	12.0573	8.69E-05	1
MA1020.1	Phatr3_J14849	Phatr3_EC	1312	1319	-	12.0573	8.69E-05	1
MA1020.1	Phatr3_J14849	Phatr3_J4	632	639	-	12.0573	8.69E-05	1
MA1020.1	Phatr3_J14849	Phatr3_J5	199	206	-	12.0573	8.69E-05	1
MA1020.1	Phatr3_J14849	Phatr3_EC	205	212	-	12.0573	8.69E-05	1
MA1020.1	Phatr3_J14849	Phatr3_J2	1731	1738	-	12.0573	8.69E-05	1
MA1020.1	Phatr3_J14849	Phatr3_EC	686	693	+	12.0573	8.69E-05	1
MA1020.1	Phatr3_J14849	Phatr3_J1	199	206	-	12.0573	8.69E-05	1
MA1020.1	Phatr3_J14849	Phatr3_J5	306	313	+	12.0573	8.69E-05	1
MA1020.1	Phatr3_J14849	Phatr3_J4	1511	1518	-	12.0573	8.69E-05	1
MA1020.1	Phatr3_J14849	Phatr3_J5	773	780	-	12.0573	8.69E-05	1
MA1020.1	Phatr3_J14849	Phatr3_EC	775	782	-	12.0573	8.69E-05	1
MA1020.1	Phatr3_J14849	Phatr3_J5	755	762	+	12.0573	8.69E-05	1
MA1020.1	Phatr3_J14849	Phatr3_EC	108	115	+	12.0573	8.69E-05	1
MA1020.1	Phatr3_J14849	Phatr3_EC	249	256	-	12.0573	8.69E-05	1
MA1020.1	Phatr3_J14849	Phatr3_J5	1729	1736	+	12.0573	8.69E-05	1
MA1020.1	Phatr3_J14849	Phatr3_J6	1768	1775	-	12.0573	8.69E-05	1
MA1020.1	Phatr3_J14849	Phatr3_J5	1834	1841	+	12.0573	8.69E-05	1
MA1020.1	Phatr3_J14849	Phatr3_J4	81	88	-	12.0573	8.69E-05	1

MA1020.1	Phatr3_J14849	Phatr3_E(	941	948	+	12.0573	8.69E-05	1
MA1020.1	Phatr3_J14849	Phatr3_J5	1052	1059	-	12.0573	8.69E-05	1
MA1020.1	Phatr3_J14849	Phatr3_J5	1013	1020	+	12.0573	8.69E-05	1
MA1020.1	Phatr3_J14849	Phatr3_J1	1038	1045	-	12.0573	8.69E-05	1
MA1020.1	Phatr3_J14849	Phatr3_J5	1136	1143	-	12.0573	8.69E-05	1
MA1020.1	Phatr3_J14849	Phatr3_E(	1718	1725	-	12.0573	8.69E-05	1
MA1020.1	Phatr3_J14849	Phatr3_E(	293	300	+	12.0573	8.69E-05	1
MA1020.1	Phatr3_J14849	Phatr3_E(	690	697	-	12.0573	8.69E-05	1
MA1020.1	Phatr3_J14849	Phatr3_E(	137	144	-	12.0573	8.69E-05	1
MA1020.1	Phatr3_J14849	Phatr3_J8	1890	1897	+	12.0573	8.69E-05	1
MA1020.1	Phatr3_J14849	Phatr3_J5	336	343	-	12.0573	8.69E-05	1
MA1020.1	Phatr3_J14849	Phatr3_J5	61	68	-	12.0573	8.69E-05	1
MA1020.1	Phatr3_J14849	Phatr3_J5	1694	1701	-	12.0573	8.69E-05	1
MA1020.1	Phatr3_J14849	Phatr3_J5	155	162	-	12.0573	8.69E-05	1
MA1020.1	Phatr3_J14849	Phatr3_E(	1402	1409	-	12.0573	8.69E-05	1
MA1020.1	Phatr3_J14849	Phatr3_E(	586	593	+	12.0573	8.69E-05	1
MA1020.1	Phatr3_J14849	Phatr3_J2	1327	1334	-	12.0573	8.69E-05	1
MA1020.1	Phatr3_J14849	Phatr3_J3	1665	1672	+	12.0573	8.69E-05	1
MA1020.1	Phatr3_J14849	Phatr3_E(	1530	1537	+	12.0573	8.69E-05	1
MA1020.1	Phatr3_J14849	Phatr3_J1	934	941	+	12.0573	8.69E-05	1
MA1020.1	Phatr3_J14849	Phatr3_J3	241	248	-	12.0573	8.69E-05	1
MA1020.1	Phatr3_J14849	Phatr3_E(	1521	1528	+	12.0573	8.69E-05	1
MA1020.1	Phatr3_J14849	Phatr3_J4	412	419	+	12.0573	8.69E-05	1
MA1020.1	Phatr3_J14849	Phatr3_E(	1697	1704	-	12.0573	8.69E-05	1
MA1020.1	Phatr3_J14849	Phatr3_J3	1809	1816	+	12.0573	8.69E-05	1
MA1020.1	Phatr3_J14849	Phatr3_J4	1686	1693	+	12.0573	8.69E-05	1
MA1020.1	Phatr3_J14849	Phatr3_J4	1273	1280	-	12.0573	8.69E-05	1
MA1020.1	Phatr3_J14849	Phatr3_J1	1189	1196	+	12.0573	8.69E-05	1
MA1020.1	Phatr3_J14849	Phatr3_J1	1149	1156	+	12.0573	8.69E-05	1
MA1020.1	Phatr3_J14849	Phatr3_J1	1147	1154	-	12.0573	8.69E-05	1
MA1020.1	Phatr3_J14849	Phatr3_J5	1631	1638	-	12.0573	8.69E-05	1
MA1020.1	Phatr3_J14849	Phatr3_J4	876	883	-	12.0573	8.69E-05	1
MA1020.1	Phatr3_J14849	Phatr3_J4	1120	1127	-	12.0573	8.69E-05	1
MA1020.1	Phatr3_J14849	Phatr3_J4	277	284	+	12.0573	8.69E-05	1
MA1020.1	Phatr3_J14849	Phatr3_J4	400	407	-	12.0573	8.69E-05	1
MA1020.1	Phatr3_J14849	Phatr3_J4	342	349	-	12.0573	8.69E-05	1
MA1020.1	Phatr3_J14849	Phatr3_J1	967	974	-	12.0573	8.69E-05	1
MA1020.1	Phatr3_J14849	Phatr3_J4	1357	1364	-	12.0573	8.69E-05	1
MA1020.1	Phatr3_J14849	EPrPhatr3	355	362	+	12.0573	8.69E-05	1
MA1020.1	Phatr3_J14849	PTsRNA00(	292	299	+	12.0573	8.69E-05	1
MA1020.1	Phatr3_J14849	Phatr3_E(	817	824	-	12.0573	8.69E-05	1
MA1020.1	Phatr3_J14849	Phatr3_E(	1851	1858	+	12.0573	8.69E-05	1
MA1020.1	Phatr3_J14849	Phatr3_E(	281	288	+	12.0573	8.69E-05	1
MA1020.1	Phatr3_J14849	Phatr3_E(	1786	1793	+	12.0573	8.69E-05	1
MA1020.1	Phatr3_J14849	Phatr3_J4	62	69	-	12.0573	8.69E-05	1
MA1020.1	Phatr3_J14849	Phatr3_J1	474	481	+	12.0573	8.69E-05	1
MA1020.1	Phatr3_J14849	Phatr3_E(	947	954	-	12.0573	8.69E-05	1
MA1020.1	Phatr3_J14849	Phatr3_J4	629	636	-	12.0573	8.69E-05	1
MA1020.1	Phatr3_J14849	Phatr3_J4	455	462	-	12.0573	8.69E-05	1
MA1020.1	Phatr3_J14849	Phatr3_J4	1673	1680	+	12.0573	8.69E-05	1
MA1020.1	Phatr3_J14849	Phatr3_J4	1161	1168	+	12.0573	8.69E-05	1
MA1020.1	Phatr3_J14849	Phatr3_J4	1213	1220	-	12.0573	8.69E-05	1
MA1020.1	Phatr3_J14849	Phatr3_J3	950	957	+	12.0573	8.69E-05	1
MA1020.1	Phatr3_J14849	Phatr3_E(	875	882	+	12.0573	8.69E-05	1

MA1020.1	Phatr3_J14849	Phatr3_J4	754	761	-	12.0573	8.69E-05	1
MA1020.1	Phatr3_J14849	Phatr3_J3	1451	1458	-	12.0573	8.69E-05	1
MA1020.1	Phatr3_J14849	Phatr3_J4	1801	1808	-	12.0573	8.69E-05	1
MA1020.1	Phatr3_J14849	Phatr3_J4	157	164	-	12.0573	8.69E-05	1
MA1020.1	Phatr3_J14849	Phatr3_J3	660	667	+	12.0573	8.69E-05	1
MA1020.1	Phatr3_J14849	Phatr3_J5	1942	1949	+	12.0573	8.69E-05	1
MA1020.1	Phatr3_J14849	Phatr3_J6	1771	1778	-	12.0573	8.69E-05	1
MA1020.1	Phatr3_J14849	Phatr3_J5	222	229	+	12.0573	8.69E-05	1
MA1020.1	Phatr3_J14849	Phatr3_J5	788	795	+	12.0573	8.69E-05	1
MA1020.1	Phatr3_J14849	Phatr3_J5	469	476	-	12.0573	8.69E-05	1
MA1020.1	Phatr3_J14849	Phatr3_E0	1258	1265	-	12.0573	8.69E-05	1
MA1020.1	Phatr3_J14849	Phatr3_E0	1950	1957	-	12.0573	8.69E-05	1
MA1020.1	Phatr3_J14849	Phatr3_J7	1918	1925	-	12.0573	8.69E-05	1
MA1020.1	Phatr3_J14849	Phatr3_J5	393	400	-	12.0573	8.69E-05	1
MA1020.1	Phatr3_J14849	Phatr3_E0	597	604	-	12.0573	8.69E-05	1
MA1020.1	Phatr3_J14849	Phatr3_J5	428	435	+	12.0573	8.69E-05	1
MA1020.1	Phatr3_J14849	Phatr3_J5	1923	1930	+	12.0573	8.69E-05	1
MA1020.1	Phatr3_J14849	Phatr3_E0	474	481	+	12.0573	8.69E-05	1
MA1020.1	Phatr3_J14849	Phatr3_J5	1326	1333	-	12.0573	8.69E-05	1
MA1020.1	Phatr3_J14849	Phatr3_J5	988	995	+	12.0573	8.69E-05	1
MA1020.1	Phatr3_J14849	Phatr3_E0	1242	1249	-	12.0573	8.69E-05	1
MA1020.1	Phatr3_J14849	Phatr3_J3	504	511	+	12.0573	8.69E-05	1
MA1020.1	Phatr3_J14849	Phatr3_J1	58	65	-	12.0573	8.69E-05	1
MA1020.1	Phatr3_J14849	Phatr3_J4	420	427	+	12.0573	8.69E-05	1
MA1020.1	Phatr3_J14849	Phatr3_J3	1487	1494	+	12.0573	8.69E-05	1
MA1020.1	Phatr3_J14849	Phatr3_J4	1880	1887	+	12.0573	8.69E-05	1
MA1020.1	Phatr3_J14849	Phatr3_J3	806	813	-	12.0573	8.69E-05	1
MA1020.1	Phatr3_J14849	Phatr3_J3	809	816	-	12.0573	8.69E-05	1
MA1020.1	Phatr3_J14849	Phatr3_J4	194	201	-	12.0573	8.69E-05	1
MA1020.1	Phatr3_J14849	Phatr3_J4	135	142	+	12.0573	8.69E-05	1
MA1020.1	Phatr3_J14849	Phatr3_E0	723	730	+	12.0573	8.69E-05	1
MA1020.1	Phatr3_J14849	Phatr3_E0	1248	1255	+	12.0573	8.69E-05	1
MA1020.1	Phatr3_J14849	Phatr3_J2	660	667	-	12.0573	8.69E-05	1
MA1020.1	Phatr3_J14849	Phatr3_J4	1200	1207	-	12.0573	8.69E-05	1
MA1020.1	Phatr3_J14849	Phatr3_E0	603	610	+	12.0573	8.69E-05	1
MA1020.1	Phatr3_J14849	Phatr3_J4	1553	1560	-	12.0573	8.69E-05	1
MA1020.1	Phatr3_J14849	Phatr3_J3	1872	1879	-	12.0573	8.69E-05	1
MA1020.1	Phatr3_J14849	Phatr3_J4	1663	1670	+	12.0573	8.69E-05	1
MA1020.1	Phatr3_J14849	Phatr3_J4	1509	1516	-	12.0573	8.69E-05	1
MA1020.1	Phatr3_J14849	Phatr3_J3	446	453	+	12.0573	8.69E-05	1
MA1020.1	Phatr3_J14849	Phatr3_J3	1680	1687	-	12.0573	8.69E-05	1
MA1020.1	Phatr3_J14849	Phatr3_J2	742	749	+	12.0573	8.69E-05	1
MA1020.1	Phatr3_J14849	Phatr3_J1	1724	1731	-	12.0573	8.69E-05	1
MA1020.1	Phatr3_J14849	Phatr3_J3	1488	1495	-	12.0573	8.69E-05	1
MA1020.1	Phatr3_J14849	Phatr3_J8	132	139	+	12.0573	8.69E-05	1
MA1020.1	Phatr3_J14849	Phatr3_J4	552	559	+	12.0573	8.69E-05	1
MA1020.1	Phatr3_J14849	Phatr3_J3	1195	1202	-	12.0573	8.69E-05	1
MA1020.1	Phatr3_J14849	Phatr3_J3	438	445	+	12.0573	8.69E-05	1
MA1020.1	Phatr3_J14849	Phatr3_J1	809	816	+	12.0573	8.69E-05	1
MA1020.1	Phatr3_J14849	Phatr3_J4	1372	1379	-	12.0573	8.69E-05	1
MA1020.1	Phatr3_J14849	Phatr3_J1	1723	1730	+	12.0573	8.69E-05	1
MA1020.1	Phatr3_J14849	Phatr3_E0	972	979	-	12.0573	8.69E-05	1
MA1020.1	Phatr3_J14849	Phatr3_E0	1384	1391	-	12.0573	8.69E-05	1
MA1020.1	Phatr3_J14849	Phatr3_J4	1386	1393	-	12.0573	8.69E-05	1

MA1020.1	Phatr3_J14849	Phatr3_EC	743	750	+	12.0573	8.69E-05	1
MA1020.1	Phatr3_J14849	Phatr3_EC	903	910	+	12.0573	8.69E-05	1
MA1020.1	Phatr3_J14849	Phatr3_J3	125	132	-	12.0573	8.69E-05	1
MA1020.1	Phatr3_J14849	Phatr3_J5	720	727	-	12.0573	8.69E-05	1
MA1020.1	Phatr3_J14849	Phatr3_J5	939	946	-	12.0573	8.69E-05	1
MA1020.1	Phatr3_J14849	Phatr3_J1	51	58	-	12.0573	8.69E-05	1
MA1020.1	Phatr3_J14849	Phatr3_J4	189	196	+	12.0573	8.69E-05	1
MA1020.1	Phatr3_J14849	Phatr3_J3	995	1002	+	12.0573	8.69E-05	1
MA1020.1	Phatr3_J14849	Phatr3_J4	1023	1030	+	12.0573	8.69E-05	1
MA1020.1	Phatr3_J14849	Phatr3_EC	1345	1352	-	12.0573	8.69E-05	1
MA1020.1	Phatr3_J14849	Phatr3_J4	800	807	-	12.0573	8.69E-05	1
MA1020.1	Phatr3_J14849	Phatr3_EC	1130	1137	+	12.0573	8.69E-05	1
MA1020.1	Phatr3_J14849	Phatr3_J2	1137	1144	-	12.0573	8.69E-05	1
MA1020.1	Phatr3_J14849	Phatr3_J4	343	350	+	12.0573	8.69E-05	1
MA1020.1	Phatr3_J14849	Phatr3_J4	412	419	-	12.0573	8.69E-05	1
MA1020.1	Phatr3_J14849	Phatr3_J4	1100	1107	-	12.0573	8.69E-05	1
MA1020.1	Phatr3_J14849	Phatr3_EC	881	888	-	12.0573	8.69E-05	1
MA1020.1	Phatr3_J14849	Phatr3_EC	804	811	-	12.0573	8.69E-05	1
MA1020.1	Phatr3_J14849	Phatr3_EC	1919	1926	+	12.0573	8.69E-05	1
MA1020.1	Phatr3_J14849	Phatr3_J4	810	817	+	12.0573	8.69E-05	1
MA1020.1	Phatr3_J14849	Phatr3_J4	1708	1715	+	12.0573	8.69E-05	1
MA1020.1	Phatr3_J14849	Phatr3_J4	602	609	-	12.0573	8.69E-05	1
MA1020.1	Phatr3_J14849	Phatr3_J4	1500	1507	-	12.0573	8.69E-05	1
MA1020.1	Phatr3_J14849	Phatr3_EC	514	521	+	12.0573	8.69E-05	1
MA1020.1	Phatr3_J14849	Phatr3_EC	792	799	+	12.0573	8.69E-05	1
MA1020.1	Phatr3_J14849	Phatr3_J4	353	360	-	12.0573	8.69E-05	1
MA1020.1	Phatr3_J14849	Phatr3_J3	570	577	+	12.0573	8.69E-05	1
MA1020.1	Phatr3_J14849	Phatr3_J3	752	759	-	12.0573	8.69E-05	1
MA1020.1	Phatr3_J14849	Phatr3_EC	50	57	+	12.0573	8.69E-05	1
MA1020.1	Phatr3_J14849	Phatr3_J3	595	602	+	12.0573	8.69E-05	1
MA1020.1	Phatr3_J14849	Phatr3_EC	139	146	-	12.0573	8.69E-05	1
MA1020.1	Phatr3_J14849	Phatr3_J4	1093	1100	+	12.0573	8.69E-05	1
MA1020.1	Phatr3_J14849	Phatr3_EC	1747	1754	-	12.0573	8.69E-05	1
MA1020.1	Phatr3_J14849	Phatr3_J4	152	159	+	12.0573	8.69E-05	1
MA1020.1	Phatr3_J14849	Phatr3_J3	526	533	-	12.0573	8.69E-05	1
MA1020.1	Phatr3_J14849	Phatr3_J3	1989	1996	+	12.0573	8.69E-05	1
MA1020.1	Phatr3_J14849	Phatr3_J4	1706	1713	-	12.0573	8.69E-05	1
MA1020.1	Phatr3_J14849	Phatr3_EC	130	137	-	12.0573	8.69E-05	1
MA1020.1	Phatr3_J14849	Phatr3_J4	366	373	+	12.0573	8.69E-05	1
MA1020.1	Phatr3_J14849	Phatr3_EC	1237	1244	-	12.0573	8.69E-05	1
MA1020.1	Phatr3_J14849	Phatr3_J3	565	572	-	12.0573	8.69E-05	1
MA1020.1	Phatr3_J14849	Phatr3_J4	1780	1787	-	12.0573	8.69E-05	1
MA1020.1	Phatr3_J14849	Phatr3_J2	1415	1422	+	12.0573	8.69E-05	1
MA1020.1	Phatr3_J14849	Phatr3_J4	574	581	+	12.0573	8.69E-05	1
MA1020.1	Phatr3_J14849	Phatr3_J3	602	609	-	12.0573	8.69E-05	1
MA1020.1	Phatr3_J14849	Phatr3_EC	1179	1186	-	12.0573	8.69E-05	1
MA1020.1	Phatr3_J14849	Phatr3_J4	643	650	+	12.0573	8.69E-05	1
MA1020.1	Phatr3_J14849	Phatr3_J4	220	227	-	12.0573	8.69E-05	1
MA1020.1	Phatr3_J14849	Phatr3_EC	441	448	-	12.0573	8.69E-05	1
MA1020.1	Phatr3_J14849	Phatr3_J4	1759	1766	-	12.0573	8.69E-05	1
MA1020.1	Phatr3_J14849	Phatr3_J4	486	493	-	12.0573	8.69E-05	1
MA1020.1	Phatr3_J14849	Phatr3_J3	1916	1923	-	12.0573	8.69E-05	1
MA1020.1	Phatr3_J14849	Phatr3_J4	1150	1157	+	12.0573	8.69E-05	1
MA1020.1	Phatr3_J14849	Phatr3_J1	1627	1634	+	12.0573	8.69E-05	1

MA1020.1	Phatr3_J14849	Phatr3_J4	255	262	-	12.0573	8.69E-05	1
MA1020.1	Phatr3_J14849	Phatr3_EC	1115	1122	+	12.0573	8.69E-05	1
MA1020.1	Phatr3_J14849	Phatr3_J6	1547	1554	-	12.0573	8.69E-05	1
MA1020.1	Phatr3_J14849	Phatr3_J1	320	327	-	12.0573	8.69E-05	1
MA1020.1	Phatr3_J14849	Phatr3_EC	288	295	-	12.0573	8.69E-05	1
MA1020.1	Phatr3_J14849	Phatr3_EC	1936	1943	-	12.0573	8.69E-05	1
MA1020.1	Phatr3_J14849	Phatr3_J4	1537	1544	+	12.0573	8.69E-05	1
MA1020.1	Phatr3_J14849	Phatr3_EC	913	920	+	12.0573	8.69E-05	1
MA1020.1	Phatr3_J14849	Phatr3_J3	1384	1391	-	12.0573	8.69E-05	1
MA1020.1	Phatr3_J14849	Phatr3_EC	218	225	-	12.0573	8.69E-05	1
MA1020.1	Phatr3_J14849	Phatr3_EC	1747	1754	-	12.0573	8.69E-05	1
MA1020.1	Phatr3_J14849	Phatr3_J1	1304	1311	-	12.0573	8.69E-05	1
MA1020.1	Phatr3_J14849	Phatr3_J3	627	634	+	12.0573	8.69E-05	1
MA1020.1	Phatr3_J14849	Phatr3_J3	730	737	-	12.0573	8.69E-05	1
MA1020.1	Phatr3_J14849	Phatr3_J3	1759	1766	-	12.0573	8.69E-05	1
MA1020.1	Phatr3_J14849	Phatr3_J3	1254	1261	+	12.0573	8.69E-05	1
MA1020.1	Phatr3_J14849	Phatr3_J4	719	726	-	12.0573	8.69E-05	1
MA1020.1	Phatr3_J14849	Phatr3_J3	1944	1951	+	12.0573	8.69E-05	1
MA1020.1	Phatr3_J14849	Phatr3_J4	125	132	-	12.0573	8.69E-05	1
MA1020.1	Phatr3_J14849	Phatr3_J4	1599	1606	-	12.0573	8.69E-05	1
MA1020.1	Phatr3_J14849	Phatr3_J3	978	985	-	12.0573	8.69E-05	1
MA1020.1	Phatr3_J14849	Phatr3_J4	254	261	-	12.0573	8.69E-05	1
MA1020.1	Phatr3_J14849	Phatr3_J4	1659	1666	+	12.0573	8.69E-05	1
MA1020.1	Phatr3_J14849	Phatr3_J4	618	625	-	12.0573	8.69E-05	1
MA1020.1	Phatr3_J14849	Phatr3_J4	710	717	+	12.0573	8.69E-05	1
MA1020.1	Phatr3_J14849	Phatr3_J4	1234	1241	-	12.0573	8.69E-05	1
MA1020.1	Phatr3_J14849	Phatr3_J4	1071	1078	-	12.0573	8.69E-05	1
MA1020.1	Phatr3_J14849	Phatr3_J2	1009	1016	-	12.0573	8.69E-05	1
MA1020.1	Phatr3_J14849	Phatr3_J4	328	335	-	12.0573	8.69E-05	1
MA1020.1	Phatr3_J14849	Phatr3_J5	1767	1774	-	12.0573	8.69E-05	1
MA1020.1	Phatr3_J14849	Phatr3_J4	1162	1169	+	12.0573	8.69E-05	1
MA1020.1	Phatr3_J14849	Phatr3_EC	325	332	-	12.0573	8.69E-05	1
MA1020.1	Phatr3_J14849	Phatr3_J4	1968	1975	-	12.0573	8.69E-05	1
MA1020.1	Phatr3_J14849	Phatr3_J3	1570	1577	+	12.0573	8.69E-05	1
MA1020.1	Phatr3_J14849	Phatr3_J4	798	805	-	12.0573	8.69E-05	1
MA1020.1	Phatr3_J14849	Phatr3_J3	1314	1321	-	12.0573	8.69E-05	1
MA1020.1	Phatr3_J14849	Phatr3_J3	783	790	-	12.0573	8.69E-05	1
MA1020.1	Phatr3_J14849	Phatr3_J4	915	922	-	12.0573	8.69E-05	1
MA1020.1	Phatr3_J14849	Phatr3_J4	642	649	-	12.0573	8.69E-05	1
MA1020.1	Phatr3_J14849	Phatr3_J4	1396	1403	-	12.0573	8.69E-05	1
MA1020.1	Phatr3_J14849	Phatr3_J7	12	19	+	12.0573	8.69E-05	1
MA1020.1	Phatr3_J14849	Phatr3_J4	1667	1674	+	12.0573	8.69E-05	1
MA1020.1	Phatr3_J14849	Phatr3_J4	531	538	-	12.0573	8.69E-05	1
MA1020.1	Phatr3_J14849	Phatr3_EC	1606	1613	+	12.0573	8.69E-05	1
MA1020.1	Phatr3_J14849	Phatr3_EC	735	742	+	12.0573	8.69E-05	1
MA1020.1	Phatr3_J14849	Phatr3_EC	313	320	+	12.0573	8.69E-05	1
MA1020.1	Phatr3_J14849	Phatr3_J3	325	332	+	12.0573	8.69E-05	1
MA1020.1	Phatr3_J14849	EPrPhatr3	974	981	+	12.0573	8.69E-05	1
MA1020.1	Phatr3_J14849	EPrPhatr3	372	379	+	12.0573	8.69E-05	1
MA1020.1	Phatr3_J14849	EPrPhatr3	1544	1551	-	12.0573	8.69E-05	1
MA1020.1	Phatr3_J14849	EPrPhatr3	1413	1420	-	12.0573	8.69E-05	1
MA1020.1	Phatr3_J14849	EPrPhatr3	1240	1247	-	12.0573	8.69E-05	1
MA1020.1	Phatr3_J14849	EPrPhatr3	1750	1757	+	12.0573	8.69E-05	1
MA1020.1	Phatr3_J14849	EPrPhatr3	1829	1836	+	12.0573	8.69E-05	1

matched sequence
GTTAACCA
GTTAACCA
GTTAACCA
GTTAACCA
GTTAACCA
GTTAACCA
GTTAACCA
GTTAACCA
GTTAACCA
GTTAACCA
GTTAACCA
GTTAACCA
GTTAACCA
GTTAACCA
GTTAACCA
GTTAACCA
GTTAACCA
GTTAACCA
GTTAACCA
GTTAACCA
GTTAACCA
GTTAACCA
GTTAACCA
GTTAACCA
GTTAACCA
GTTAACCA
GTTAACCA
GTTAACCA
GTTAACCA
GTTAACCA
GTTAACCA
GTTAACCA
GTTAACCA
GTTAACCA
GTTAACCA
GTTAACCA
GTTAACCA
GTTAACCA
GTTAACCA
GTTAACCA
GTTAACCA
GTTAACCA
GTTAACCA
GTTAACCA
GTTAACCA
GTTAACCA
GTTAACCA
GTTAACCA
GTTAACCA
GTTAACCA
GTTAACCA
GTTAACCA
GTTAACCA

GTTAACCA
GTTAACCA
GTTAACCA
GTTAACCA
GTTAACCA
GTTAACCA
GTTAACCA
GTTAACCA
GTTAACCA
GTTAACCC
GTTAACCC
GTTAACCC
GTTAACCC
GTTAACCC
GTTAACCC
GTTAACCC
GTTAACCC
GTTAACCC
GTTAACCC
GTTAACCC
GTTAACCC
GTTAACCC
GTTAACCC
GTTAACCC
GTTAACCC
GTTAACCC
GTTAACCC
GTTAACCC
GTTAACCC
GTTAACCC
GTTAACCC
GTTAACCC
GTTAACCC
GTTAACCC
GTTAACCC
GTTAACCC
GTTAACCC
GTTAACCC
GTTAACCC
GTTAACCC
GTTAACCC
GTTAACCC
GTTAACCC
GTTAACCC
GTTAACCC
GTTAACCC
GTTAACCC
GTTAACCC
GTTAACCC
GTTAACCC
GTTAACCC
GTTAACCC
CTTAACCA
GTTAACCG

TTTAACCA
TTTAACCA
CTTAACCA
TTTAACCA
CTTAACCA
CTTAACCA
CTTAACCA
CTTAACCA
TTTAACCA
TTTAACCA
TTTAACCA
TTTAACCA
CTTAACCA
CTTAACCA
GTTAACCG
TTTAACCA
CTTAACCA
TTTAACCA
CTTAACCA
TTTAACCA
TTTAACCA
TTTAACCA
GTTAACCG
CTTAACCA
CTTAACCA
TTTAACCA
GTTAACCG
CTTAACCA
TTTAACCA
TTTAACCA
CTTAACCA
CTTAACCA
CTTAACCA
CTTAACCA
TTTAACCA
TTTAACCA
TTTAACCA
CTTAACCA
CTTAACCA
TTTAACCA
CTTAACCA
CTTAACCA
GTTAACCG
GTTAACCG
CTTAACCA
CTTAACCA
CTTAACCA
TTTAACCA
GTTAACCG
CTTAACCA
CTTAACCA
GTTAACCG
TTTAACCA
GTTAACCG

GTTAACCG
GTTAACCG
GTTAACCG
TTTAACCA
TTTAACCA
TTTAACCA
TTTAACCA
TTTAACCA
CTTAACCA
TTTAACCA
TTTAACCA
CTTAACCA
TTTAACCA
TTTAACCA
TTTAACCA
TTTAACCA
CTTAACCA
CTTAACCA
TTTAACCA
TTTAACCA
TTTAACCA
CTTAACCA
CTTAACCA
TTTAACCA
CTTAACCA
TTTAACCA
TTTAACCA
CTTAACCA
GTTAACCG
TTTAACCA
TTTAACCA
GTTAACCG
GTTAACCG
CTTAACCA
GTTAACCG
CTTAACCA
TTTAACCA
GTTAACCG
GTTAACCG
CTTAACCA
GTTAACCG
CTTAACCA
GTTAACCG
CTTAACCA
GTTAACCG
TTTAACCA
TTTAACCA
TTTAACCA
TTTAACCA
TTTAACCA
TTTAACCA
TTTAACCA
TTTAACCA
TTTAACCA

TTTAACCA
TTTAACCA
TTTAACCA
TTTAACCA
TTTAACCA
GTTAACCG
TTTAACCA
CTTAACCA
GTTAACCG
TTTAACCA
TTTAACCA
CTTAACCA
CTTAACCA
CTTAACCA
GTTAACCG
TTTAACCA
TTTAACCA
TTTAACCA
CTTAACCA
TTTAACCA
GTTAACCG
GTTAACCG
TTTAACCA
GTTAACCG
TTTAACCA
CTTAACCA
TTTAACCA
TTTAACCA
TTTAACCA
GTTAACCG
GTTAACCG
GTTAACCG
CTTAACCA
TTTAACCA
CTTAACCA
GTTAACCG
TTTAACCA
GTTAACCG
TTTAACCA
TTTAACCA
GTTAACCG
TTTAACCA
TTTAACCA
TTTAACCA
TTTAACCA
TTTAACCA
TTTAACCA
GTTAACCG
GTTAACCG
TTTAACCA
TTTAACCA
TTTAACCA
CTTAACCA
TTTAACCA

GTTAACCA
GTTAACCA
GTTAACCA
GTTAACCA
GTTAACCA
GTTAACCA
GTTAACCA
GTTAACCA
GTTAACCA
GTTAACCA
GTTAACCA
GTTAACCA
GTTAACCA
GTTAACCA
GTTAACCA
GTTAACCA
GTTAACCA
GTTAACCA
GTTAACCA
GTTAACCA
GTTAACCA
GTTAACCA
GTTAACCA
GTTAACCA
GTTAACCA
GTTAACCA
GTTAACCA
GTTAACCA
GTTAACCA
GTTAACCA
GTTAACCA
GTTAACCA
GTTAACCA
GTTAACCA
GTTAACCA
GTTAACCA
GTTAACCA
GTTAACCA
GTTAACCA
GTTAACCA
GTTAACCA
GTTAACCA
GTTAACCA
GTTAACCA
GTTAACCA
GTTAACCA
GTTAACCA
GTTAACCA
GTTAACCA
GTTAACCA
GTTAACCA
GTTAACCA
GTTAACCA
GTTAACCA

GTTAACCA
GTTAACCA
GTTAACCA
GTTAACCA
GTTAACCA
GTTAACCA
GTTAACCA
GTTAACCA
GTTAACCA
GTTAACCA
GTTAACCA
GTTAACCA
GTTAACCA
GTTAACCA
GTTAACCA
GTTAACCA
GTTAACCA
GTTAACCA
GTTAACCA
GTTAACCA
GTTAACCA
GTTAACCA
GTTAACCA
GTTAACCA
GTTAACCA
GTTAACCA
GTTAACCA
GTTAACCA
GTTAACCA
GTTAACCA
GTTAACCA
GTTAACCA
GTTAACCA
GTTAACCA
GTTAACCA
GTTAACCA
GTTAACCA
GTTAACCA
GTTAACCA
GTTAACCA
GTTAACCA
GTTAACCA
GTTAACCA
GTTAACCA
GTTAACCA
GTTAACCA
GTTAACCA
GTTAACCA
GTTAACCA
GTTAACCA
GTTAACCA
GTTAACCA
GTTAACCA
GTTAACCA

GTTAACCA
GTTAACCA
GTTAACCA
GTTAACCA
GTTAACCA
GTTAACCA
GTTAACCA
GTTAACCA
GTTAACCA
GTTAACCA
GTTAACCA
GTTAACCA
GTTAACCA
GTTAACCA
GTTAACCA
GTTAACCA
GTTAACCA
GTTAACCA
GTTAACCA
GTTAACCA
GTTAACCA
GTTAACCA
GTTAACCA
GTTAACCA
GTTAACCA
GTTAACCA
GTTAACCA
GTTAACCA
GTTAACCA
GTTAACCA
GTTAACCA
GTTAACCA
GTTAACCA
GTTAACCA
GTTAACCA
GTTAACCA
GTTAACCA
GTTAACCA
GTTAACCA
GTTAACCA
GTTAACCA
GTTAACCA
GTTAACCA
GTTAACCA
GTTAACCA
GTTAACCA
GTTAACCA
GTTAACCA
GTTAACCA
GTTAACCA
GTTAACCA
GTTAACCA
GTTAACCA
GTTAACCA

GTTAACCA
GTTAACCA
GTTAACCA
GTTAACCA
GTTAACCA
GTTAACCA
GTTAACCA
GTTAACCA
GTTAACCA
GTTAACCA
GTTAACCA
GTTAACCA
GTTAACCA
GTTAACCA
GTTAACCA
GTTAACCA
GTTAACCA
GTTAACCA
GTTAACCA
GTTAACCA
GTTAACCA
GTTAACCA
GTTAACCA
GTTAACCA
GTTAACCA
GTTAACCA
GTTAACCA
GTTAACCA
GTTAACCA
GTTAACCA
GTTAACCA
GTTAACCA
GTTAACCA
GTTAACCA
GTTAACCA
GTTAACCA
GTTAACCA
GTTAACCA
GTTAACCC
GTTAACCC
GTTAACCC
GTTAACCC
GTTAACCC
GTTAACCC
GTTAACCC
GTTAACCC
GTTAACCC
GTTAACCC
GTTAACCC
GTTAACCC
GTTAACCC
GTTAACCC
GTTAACCC
GTTAACCC

GTTAACCC
GTTAACCC
GTTAACCC
GTTAACCC
GTTAACCC
GTTAACCC
GTTAACCC
GTTAACCC
GTTAACCC
GTTAACCC
GTTAACCC
GTTAACCC
GTTAACCC
GTTAACCC
GTTAACCC
GTTAACCC
GTTAACCC
GTTAACCC
GTTAACCC
GTTAACCC
GTTAACCC
GTTAACCC
GTTAACCC
GTTAACCC
GTTAACCC
GTTAACCC
GTTAACCC
GTTAACCC
GTTAACCC
GTTAACCC
GTTAACCC
GTTAACCC
GTTAACCC
GTTAACCC
GTTAACCC
GTTAACCC
GTTAACCC
GTTAACCC
GTTAACCC
GTTAACCC
GTTAACCC
GTTAACCC
GTTAACCC
GTTAACCC
GTTAACCC
GTTAACCC
GTTAACCC
GTTAACCC
GTTAACCC
GTTAACCC
GTTAACCC
GTTAACCC
GTTAACCC
GTTAACCC

GTTAACCC
GTTAACCC
GTTAACCC
GTTAACCC
GTTAACCC
GTTAACCC
GTTAACCC
GTTAACCC
GTTAACCC
GTTAACCC
GTTAACCC
GTTAACCC
GTTAACCC
GTTAACCC
GTTAACCC
GTTAACCC
GTTAACCC
GTTAACCC
GTTAACCC
GTTAACCC
GTTAACCC
GTTAACCC
GTTAACCC
GTTAACCC
GTTAACCC
GTTAACCC
GTTAACCC
GTTAACCC
GTTAACCC
GTTAACCC
GTTAACCC
GTTAACCC
GTTAACCC
GTTAACCC
GTTAACCC
GTTAACCC
GTTAACCC
GTTAACCC
GTTAACCC
GTTAACCC
GTTAACCC
GTTAACCC
GTTAACCC
GTTAACCC
GTTAACCC
GTTAACCC
GTTAACCC
GTTAACCC
GTTAACCC
GTTAACCC
GTTAACCC
GTTAACCC
GTTAACCC
GTTAACCC

GTTAACCC
GTTAACCC
GTTAACCC
GTTAACCC
GTTAACCC
GTTAACCC
GTTAACCC
GTTAACCC
GTTAACCC
GTTAACCC
GTTAACCC
GTTAACCC
GTTAACCC
GTTAACCC
GTTAACCC
GTTAACCC
GTTAACCC
GTTAACCC
GTTAACCC
GTTAACCC
GTTAACCC
GTTAACCC
GTTAACCC
GTTAACCC
GTTAACCC
GTTAACCC
GTTAACCC
GTTAACCC
GTTAACCC
GTTAACCC
TTTAACCA
GTTAACCG
CTTAACCA
CTTAACCA
TTTAACCA
TTTAACCA
TTTAACCA
TTTAACCA
TTTAACCA
GTTAACCG
TTTAACCA
TTTAACCA
TTTAACCA
TTTAACCA
GTTAACCG
GTTAACCG
CTTAACCA
CTTAACCA
GTTAACCG
CTTAACCA
CTTAACCA
TTTAACCA
TTTAACCA
CTTAACCA

CTTAACCA
CTTAACCA
CTTAACCA
CTTAACCA
TTTAACCA
GTTAACCG
TTTAACCA
TTTAACCA
CTTAACCA
GTTAACCG
GTTAACCG
CTTAACCA
TTTAACCA
TTTAACCA
CTTAACCA
GTTAACCG
GTTAACCG
CTTAACCA
TTTAACCA
TTTAACCA
CTTAACCA
TTTAACCA
TTTAACCA
TTTAACCA
TTTAACCA
TTTAACCA
CTTAACCA
CTTAACCA
CTTAACCA
CTTAACCA
TTTAACCA
TTTAACCA
TTTAACCA
CTTAACCA
TTTAACCA
TTTAACCA
GTTAACCG
TTTAACCA
CTTAACCA
CTTAACCA
TTTAACCA
GTTAACCG
GTTAACCG
GTTAACCG
CTTAACCA
CTTAACCA
TTTAACCA
TTTAACCA
TTTAACCA
TTTAACCA
TTTAACCA
TTTAACCA
TTTAACCA
TTTAACCA

TTTAACCA
TTTAACCA
CTTAACCA
TTTAACCA
TTTAACCA
TTTAACCA
CTTAACCA
TTTAACCA
GTTAACCG
GTTAACCG
TTTAACCA
CTTAACCA
TTTAACCA
TTTAACCA
CTTAACCA
CTTAACCA
CTTAACCA
CTTAACCA
CTTAACCA
CTTAACCA
CTTAACCA
CTTAACCA
CTTAACCA
GTTAACCG
GTTAACCG
TTTAACCA
TTTAACCA
TTTAACCA
TTTAACCA
GTTAACCG
GTTAACCG
GTTAACCG
TTTAACCA
TTTAACCA
GTTAACCG
CTTAACCA
TTTAACCA
TTTAACCA
TTTAACCA
CTTAACCA
CTTAACCA
CTTAACCA
CTTAACCA
CTTAACCA
TTTAACCA
CTTAACCA
CTTAACCA
CTTAACCA
CTTAACCA
CTTAACCA
CTTAACCA
CTTAACCA
CTTAACCA
CTTAACCA

CTTAACCA
CTTAACCA
GTTAACCG
GTTAACCG
CTTAACCA
CTTAACCA
CTTAACCA
CTTAACCA
CTTAACCA
CTTAACCA
CTTAACCA
CTTAACCA
CTTAACCA
CTTAACCA
GTTAACCG
CTTAACCA
CTTAACCA
TTTAACCA
TTTAACCA
CTTAACCA
TTTAACCA
TTTAACCA
TTTAACCA
TTTAACCA
GTTAACCG
TTTAACCA
TTTAACCA
CTTAACCA
CTTAACCA
GTTAACCG
CTTAACCA
CTTAACCA
CTTAACCA
TTTAACCA
CTTAACCA
CTTAACCA
CTTAACCA
TTTAACCA
GTTAACCG
GTTAACCG
CTTAACCA
CTTAACCA
TTTAACCA
TTTAACCA
TTTAACCA
CTTAACCA
TTTAACCA
TTTAACCA
CTTAACCA
TTTAACCA
CTTAACCA
GTTAACCG
GTTAACCG
TTTAACCA

TTTAACCA
GTTAACCG
CTTAACCA
CTTAACCA
CTTAACCA
CTTAACCA
TTTAACCA
CTTAACCA
TTTAACCA
GTTAACCG
TTTAACCA
TTTAACCA
GTTAACCG
TTTAACCA
TTTAACCA
TTTAACCA
GTTAACCG
GTTAACCG
GTTAACCG
GTTAACCG
GTTAACCG
TTTAACCA
CTTAACCA
CTTAACCA
CTTAACCA
CTTAACCA
CTTAACCA
CTTAACCA
CTTAACCA
GTTAACCG
TTTAACCA
CTTAACCA
CTTAACCA
GTTAACCG
GTTAACCG
TTTAACCA
GTTAACCG
CTTAACCA
CTTAACCA
GTTAACCG
TTTAACCA
TTTAACCA
TTTAACCA
GTTAACCG
TTTAACCA
TTTAACCA
GTTAACCG
TTTAACCA
TTTAACCA
TTTAACCA
TTTAACCA
TTTAACCA
GTTAACCG
TTTAACCA

TTTAACCA
CTTAACCA
GTTAACCG
GTTAACCG
GTTAACCG
TTTAACCA
TTTAACCA
TTTAACCA
GTTAACCG
CTTAACCA
TTTAACCA
GTTAACCG
GTTAACCG
CTTAACCA
CTTAACCA
TTTAACCA
CTTAACCA
TTTAACCA
CTTAACCA
TTTAACCA
GTTAACCG
GTTAACCG
TTTAACCA
TTTAACCA
TTTAACCA
TTTAACCA
TTTAACCA
TTTAACCA
TTTAACCA
GTTAACCG
TTTAACCA
TTTAACCA
TTTAACCA
GTTAACCG
TTTAACCA
CTTAACCA
TTTAACCA
TTTAACCA
GTTAACCG
GTTAACCG
TTTAACCA
TTTAACCA
GTTAACCG
CTTAACCA
TTTAACCA
GTTAACCG
GTTAACCG
GTTAACCG
GTTAACCG
TTTAACCA
TTTAACCA
TTTAACCA
CTTAACCA
TTTAACCA

CTTAACCA
CTTAACCA
GTTAACCG
GTTAACCG
CTTAACCA
CTTAACCA
TTTAACCA
TTTAACCA
GTTAACCG
GTTAACCG
GTTAACCG
GTTAACCG
GTTAACCG
GTTAACCG
GTTAACCG
CTTAACCA
CTTAACCA
TTTAACCA
TTTAACCA
CTTAACCA
CTTAACCA
GTTAACCG
TTTAACCA
TTTAACCA
TTTAACCA
TTTAACCA
CTTAACCA
CTTAACCA
TTTAACCA
GTTAACCG
GTTAACCG
GTTAACCG
GTTAACCG
GTTAACCG
TTTAACCA
TTTAACCA
GTTAACCG
CTTAACCA
CTTAACCA
CTTAACCA
CTTAACCA
CTTAACCA
GTTAACCG
TTTAACCA
TTTAACCA
CTTAACCA
TTTAACCA
CTTAACCA
TTTAACCA
CTTAACCA
TTTAACCA
CTTAACCA
CTTAACCA
CTTAACCA

TTTAACCA
CTTAACCA
CTTAACCA
TTTAACCA
TTTAACCA
GTTAACCG
CTTAACCA
TTTAACCA
TTTAACCA
TTTAACCA
TTTAACCA
GTTAACCG
TTTAACCA
GTTAACCG
GTTAACCG
CTTAACCA
CTTAACCA
CTTAACCA
CTTAACCA
CTTAACCA
GTTAACCG
CTTAACCA
GTTAACCG
CTTAACCA
CTTAACCA
CTTAACCA
CTTAACCA
TTTAACCA
TTTAACCA
CTTAACCA
TTTAACCA
GTTAACCG
GTTAACCG
TTTAACCA
TTTAACCA
TTTAACCA
CTTAACCA
GTTAACCG
TTTAACCA
GTTAACCG
TTTAACCA
TTTAACCA
CTTAACCA
CTTAACCA
CTTAACCA
TTTAACCA
TTTAACCA
TTTAACCA
CTTAACCA
TTTAACCA
TTTAACCA
CTTAACCA
CTTAACCA
CTTAACCA

TTTAACCA
CTTAACCA
CTTAACCA
CTTAACCA
CTTAACCA
TTTAACCA
TTTAACCA
TTTAACCA
TTTAACCA
GTTAACCG
GTTAACCG
GTTAACCG
GTTAACCG
TTTAACCA
TTTAACCA
TTTAACCA
GTTAACCG
GTTAACCG
TTTAACCA
TTTAACCA
CTTAACCA
TTTAACCA
TTTAACCA
CTTAACCA
CTTAACCA
GTTAACCG
TTTAACCA
TTTAACCA
GTTAACCG
GTTAACCG
TTTAACCA
TTTAACCA
CTTAACCA
TTTAACCA
TTTAACCA
TTTAACCA
CTTAACCA
GTTAACCG
GTTAACCG
GTTAACCG
TTTAACCA
TTTAACCA
TTTAACCA
TTTAACCA
GTTAACCG
CTTAACCA
CTTAACCA
CTTAACCA
TTTAACCA
TTTAACCA
CTTAACCA
CTTAACCA
CTTAACCA
GTTAACCG

GTTAACCG
TTTAACCA
TTTAACCA
TTTAACCA
GTTAACCG
CTTAACCA
TTTAACCA
TTTAACCA
TTTAACCA
TTTAACCA
TTTAACCA
TTTAACCA
CTTAACCA
TTTAACCA
TTTAACCA
TTTAACCA
CTTAACCA
CTTAACCA
TTTAACCA
TTTAACCA
GTTAACCG
CTTAACCA
CTTAACCA
TTTAACCA
CTTAACCA
CTTAACCA
TTTAACCA
CTTAACCA
TTTAACCA
CTTAACCA
TTTAACCA
TTTAACCA
CTTAACCA
GTTAACCG
CTTAACCA
CTTAACCA
CTTAACCA
TTTAACCA
TTTAACCA
TTTAACCA
TTTAACCA
TTTAACCA
TTTAACCA
CTTAACCA
CTTAACCA
TTTAACCA
GTTAACCG
GTTAACCG
TTTAACCA
TTTAACCA
TTTAACCA
CTTAACCA
TTTAACCA
TTTAACCA

TTTAACCA
CTTAACCA
CTTAACCA
TTTAACCA
TTTAACCA
TTTAACCA
TTTAACCA
CTTAACCA
CTTAACCA
TTTAACCA
GTTAACCG
GTTAACCG
TTTAACCA
CTTAACCA
TTTAACCA
GTTAACCG
GTTAACCG
TTTAACCA
TTTAACCA
TTTAACCA
GTTAACCG
GTTAACCG
TTTAACCA
TTTAACCA
TTTAACCA
TTTAACCA
GTTAACCG
GTTAACCG
CTTAACCA
CTTAACCA
CTTAACCA
TTTAACCA
TTTAACCA
TTTAACCA
TTTAACCA
TTTAACCA
TTTAACCA
GTTAACCG
GTTAACCG
CTTAACCA
GTTAACCG
TTTAACCA
TTTAACCA
TTTAACCA
GTTAACCG
CTTAACCA
GTTAACCG
GTTAACCG
GTTAACCG
TTTAACCA
CTTAACCA
TTTAACCA
TTTAACCA
GTTAACCG

TTTAACCA
TTTAACCA
TTTAACCA
TTTAACCA
GTTAACCG
TTTAACCA
TTTAACCA
TTTAACCA
TTTAACCA
TTTAACCA
TTTAACCA
GTTAACCG
TTTAACCA
TTTAACCA
TTTAACCA
TTTAACCA
TTTAACCA
TTTAACCA
TTTAACCA
CTTAACCA
TTTAACCA
GTTAACCG
GTTAACCG
CTTAACCA
GTTAACCG
GTTAACCG
GTTAACCG
GTTAACCG
GTTAACCG
TTTAACCA
TTTAACCA
TTTAACCA
TTTAACCA
TTTAACCA
GTTAACCG
CTTAACCA
TTTAACCA
CTTAACCA
TTTAACCA
TTTAACCA
TTTAACCA
TTTAACCA
TTTAACCA
TTTAACCA
TTTAACCA
TTTAACCA
CTTAACCA
TTTAACCA
TTTAACCA
TTTAACCA
TTTAACCA
TTTAACCA
TTTAACCA
TTTAACCA
