# Supplementary material for: Cryptochrome PtCPF1 regulates high temperature acclimation of marine diatoms through coordination of iron and phosphorus uptake
Source: ISME J. 2024 Jan 10;18(1):wrad019. doi: 10.1093/ismejo/wrad019 (PMC10837835; doi:10.1093/ismejo/wrad019)
Supplement: 20231201_Supplementary_tables_S9_wrad019 [file 20231201_supplementary_tables_s9_wrad019.pdf]

Table S9 Source data of PtCPF1 homologs transcripts abundance at temperature, salinity, PAR, and NO3- from Tara Ocean.

Temperature	Salinity	PAR	NO3	Abundance
16.86368	35.68623	23.35792	1.10387	0.00125
18.82554	36.41274	21.53351	0.80803	0.00181
22.9393	36.39483	14.27785	1.03724	0.00311
13.99372	35.12152	1.09716	5.01314	0.00384
19.31278	36.51314	12.1814	1.27972	0.00507
20.4812	36.59675	21.50016	1.00992	0.00526
19.31278	36.51314	12.1814	1.27972	0.00539
15.01336	35.3233	9.59824	2.42629	0.0063
17.62714	36.28529	16.15969	0.12085	0.00665
20.4812	36.59675	21.74523	1.00992	0.00706
13.99372	35.12152	1.09716	5.01314	0.00745
27.48838	34.93996	25.38722	0.53247	1.00869
24.81217	36.21913	7.54851	0.4923	1.00903
22.9393	36.39483	13.27219	1.03724	0.01013
13.99372	35.12152	1.09716	5.01314	0.01048
20.15073	36.57475	20.97785	0.67688	0.01163
16.86368	35.68623	23.35792	1.10387	0.01345
23.37483	37.07658	35.22916	0.18597	0.01353
26.21844	35.12903	29.35013	2.96409	1.01572
13.04322	34.87074	0.06885	1.26461	0.01582
19.37309	36.54056	12.41192	0.86194	0.01651
14.31675	35.99167	9.22902	3.20922	0.0197
27.95648	34.54545	30.18933	0.53247	1.02065
18.82554	36.41274	21.07271	0.80803	0.02197
19.77001	36.35839	22.20997	1.76308	0.02252
20.07775	36.32553	40.40034	0.02533	0.0288
17.30301	36.22961	25.01212	0.29602	0.0307
25.06443	36.41678	33.58652	0.4168	0.03086
17.62714	36.28529	16.15969	0.12085	0.03683
20.4812	36.59675	21.48562	1.00992	0.04939
23.37483	37.07658	30.71043	0.18597	0.05517
19.77001	36.35839	22.20997	1.76308	0.06202
17.30301	36.22961	25.60687	0.29602	0.07372
25.1646	36.32553	40.40034	0.02533	0.07518
14.31675	35.99167	10.05958	3.20922	0.07618
26.47861	33.84798	22.24619	3.74079	0.07753
13.6805	34.79393	19.43916	2.43963	0.09185
18.82554	36.41274	21.07271	0.80803	0.093
26.47861	33.84798	21.24968	3.74079	0.10779
27.95648	34.54545	29.63899	0.53247	1.11075
27.48838	34.93996	25.55437	1.6736	1.13226
20.07775	36.32553	40.40034	1.76308	0.13859
19.77001	36.35839	22.20997	13.01071	0.1401
24.95889	34.76454	24.43905	1.54529	0.14746
25.68137	36.52596	17.26571	3.74079	0.15547
26.47861	33.84798	22.24619	0.43699	0.15689
25.68137	36.52596	17.26571	13.59252	0.19022
24.95889	34.76454	24.43905	3.20922	0.25566
14.31675	35.99167	9.22902	1.54529	0.25986
25.68137	36.52596	17.26571	2.96409	0.27725
17.30301	36.22961	25.01212	1.26461	0.39685
13.04322	34.87074	0.05459	0.63268	0.45395
26.3136	36.61804	23.00318	24.90319	1.48264
20.15073	36.57475	21.23694	3.20922	0.60412
14.31675	35.99167	9.22902	0.12085	0.61591
17.62714	36.28529	16.22539	2.43963	0.63434
13.6805	34.79393	16.7857	5.01314	0.68068
13.99372	35.12152	1.09716	1.6736	0.73318

15.01336	35.3233	9.43583	18.43419	0.80297
13.04322	34.87074	0.06885	1.27972	0.86762
26.21844	35.12903	29.35013	0.53247	1.8755
27.95648	34.54545	30.18933	3.74079	1.8807
26.47861	33.84798	22.24619	1.10387	1.00698
16.8632	35.68659	24.30143	18.43419	1.052
19.77001	36.35839	22.20997	13.01071	0.24834
24.95889	34.76454	24.43905	18.43419	1.27784
24.95889	34.76454	24.43905	24.90319	2.58289
